# Solving the taxonomic identity of *Pseudotomentellatristis* s.l. (Thelephorales, Basidiomycota) – a multi-gene phylogeny and taxonomic review, integrating ecological and geographical data

**DOI:** 10.3897/mycokeys.50.32432

**Published:** 2019-04-04

**Authors:** Sten Svantesson, Karl-Henrik Larsson, Urmas Kõljalg, Tom W. May, Patrik Cangren, R. Henrik Nilsson, Ellen Larsson

**Affiliations:** 1 Department of Biological and Environmental Sciences, University of Gothenburg, Box 463, 405 30 Göteborg, Sweden; 2 Gothenburg Global Biodiversity Centre, Box 461, 405 30 Göteborg, Sweden; 3 Royal Botanic Gardens Victoria, Birdwood Ave, Melbourne, Victoria 3004, Australia; 4 The Mycological Herbarium, Natural History Museum, University of Oslo, P.O. Box 1172, Blindern, 0318 Oslo, Norway; 5 Natural History Museum, University of Tartu, 14a Ravila, 50411 Tartu, Estonia; 6 Institute of Ecology and Earth Sciences, University of Tartu, 14a Ravila, 50411 Tartu, Estonia

**Keywords:** Corticioid fungi, ectomycorrhiza, taxonomy, species complex, molecular systematics, species tree, STACEY, UNITE database

## Abstract

*P.tristis* is an ectomycorrhizal, corticioid fungus whose name is frequently assigned to collections of basidiomata as well as root tip and soil samples from a wide range of habitats and hosts across the northern hemisphere. Despite this, its identity is unclear; eight heterotypic taxa have in major reviews of the species been considered synonymous with or morphologically similar to *P.tristis*, but no sequence data from type specimens have been available.

With the aim to clarify the taxonomy, systematics, morphology, ecology and geographical distribution of *P.tristis* and its morphologically similar species, we studied their type specimens as well as 147 basidiomata collections of mostly North European material.

We used gene trees generated in BEAST 2 and PhyML and species trees estimated in STACEY and ASTRAL to delimit species based on the ITS, LSU, Tef1α and mtSSU regions. We enriched our sampling with environmental ITS sequences from the UNITE database.

We found the *P.tristis* group to contain 13 molecularly and morphologically distinct species. Three of these, *P.tristis*, *P.umbrina* and *P.atrofusca*, are already known to science, while ten species are here described as new: *P.sciastra***sp. nov.**, *P.tristoides***sp. nov.**, *P.umbrinascens***sp. nov.**, *P.pinophila***sp. nov.**, *P.alnophila***sp. nov.**, *P.alobata***sp. nov.**, *P.pluriloba***sp. nov.**, *P.abundiloba***sp. nov.**, *P.rotundispora***sp. nov.** and *P.media***sp. nov**.

We discovered *P.rhizopunctata* and *P.atrofusca* to form a sister clade to all other species in *P.tristis* s.l. These two species, unlike all other species in the *P.tristis* complex, are dimitic.

In this study, we designate epitypes for *P.tristis*, *P.umbrina* and *Hypochnopsisfuscata* and lectotypes for *Auriculariaphylacteris* and *Thelephorabiennis*. We show that the holotype of *Hypochnussitnensis* and the lectotype of *Hypochnopsisfuscata* are conspecific with *P.tristis*, but in the absence of molecular information we regard *Pseudotomentellalongisterigmata* and *Hypochnusrhacodium* as doubtful taxa due to their aberrant morphology. We confirm *A.phylacteris*, *Tomentellabiennis* and *Septobasidiumarachnoideum* as excluded taxa, since their morphology clearly show that they belong to other genera. A key to the species of the *P.tristis* group is provided.

We found *P.umbrina* to be a common species with a wide, Holarctic distribution, forming ectomycorrhiza with a large number of host species in habitats ranging from tropical forests to the Arctic tundra. The other species in the *P.tristis* group were found to be less common and have narrower ecological niches.

## Introduction

Species of the genus *Pseudotomentella* Svrček are recognised by their smooth, corticioid, membranaceous basidiomata, bi- or trifurcately echinulate basidiospores and their lack of cystidia (Larsen 1971a, Stalpers 1993, Kõljalg 1996). All species, for which a life strategy has been confirmed, are ectomycorrhizal (Agerer 1994, Kõljalg et al. 2000, Cline et al. 2005, Di Marino et al. 2007, Trocha et al. 2012, Branco et al. 2013, Malysheva et al. 2016) and the genus is widely distributed throughout the northern hemisphere (Larsen 1971a, Kõljalg 1996). Basidiomata are formed on the underside of dead wood, turf and stones, where their spores are probably dispersed by insects, as found by a study on a species in the closely related genus *Tomentella* Pers. ex Pat. (Lilleskov and Bruns 2005).

*Pseudotomentellatristis* (P.Karst.) M.J.Larsen is characterised by its brown to bluish-grey, sometimes green-tinged basidiomata, simple septate, monomitic hyphal system, wide subicular hyphae and large, yellow to brown basidiospores (Larsen 1971a, Stalpers 1993, Kõljalg 1996). In this current, morphological delimitation of the species, it is probably the most commonly collected *Pseudotomentella* species in the world: out of 1038 herbarium specimens registered in GBIF (13–08–2018), 497 are attributed to *P.tristis* – the second most common species being *Pseudotomentellamucidula* (P.Karst.) Svrček with a total of 230 specimens. Even though there is no taxonomic study currently linking the type of *P.tristis* to molecular information, it is also a name frequently assigned to sequences recovered in molecular ecology studies of ectomycorrhizal communities in soil and on root tip samples (e.g. Kõljalg et al. 2000, Cline et al. 2005, Hrynkiewicz et al. 2009, Walbert et al. 2010, Izumi and Finlay 2011, Argüelles-Moyao et al. 2017), some of which even report it to constitute one of the most common species found (Kõljalg et al. 2000, Izumi and Finlay 2011). Entries in international sequence and specimen databases from 15 countries in the northern hemisphere indicate that it is a very widespread species (Kõljalg et al. 2005, Clark et al. 2015, GBIF 13–08–2018, Nilsson et al. 2019). Concordantly, it also has a very large ecological amplitude: sequences attributed to *P.tristis* have been encountered in habitats and with hosts ranging from the Swedish tundra with *Salixpolaris* Wahlenb. (Hrynkiewicz et al. 2009) to the neotropics of Mexico with *Abiesreligiosa* (Kunth) Schltdl. & Cham. (Argüelles-Moyao et al. 2017).

Taxonomically and nomenclaturally, *P.tristis* is a species with a long history. Based on French material, Bulliard (1790) described *Auriculariaphylacteris* Bull. – a fungus with a large corticioid basidiome, a plicated base and an initially pale, but with maturity darkening hymenium. Fries (1821) described *Thelephorabiennis* Fr. with reference to *A.phylacteris*.

In 1828, Fries introduced the name *Thelephoraumbrina* Fr. to describe a soft, brown, effused basidiome, which he stated that he had seen alive (Fries 1828).

Karsten (1889) raised a subspecies, Hypochnussubfuscusssp.tristis P.Karst., that he had previously described, based on Finnish material (Karsten 1883), to the level of species, giving it the name *Hypochnustristis* (P.Karst.) P.Karst. In his protologue, he wrote of it as having a wool or felt-like basidiome and a thick, blackish hymenium, with hues of olive brown or green, coloured brown by the detaching spores. He noted the spores to be roundedly angular, aculeate, yellow or brown and 8–12 µm in diameter. In 1889, Karsten also described *Hypochnopsisfuscata* P.Karst. – a second species from Finland, whose description is similar to that of *H.tristis*, except that, according to the author, the colour of the hymenium is bluish-black and the spores are smooth, bluish with a dark wall and 3–4 µm in diameter (Karsten 1889).

Bresadola (1897) described the species *Hypochnussitnensis* Bres. from a Hungarian basidiome with a soft, chestnut brown colour, a smoke-coloured hymenium and spores similar in size to those of *H.tristis*.

Berkeley and Broome (1873) created the new name *Thelephoraarachnoidea* Berk. & Broome for their sparse description of a basidiome with a powdery, grey hymenium and a soft, black subiculum, based on a collection made in Sri Lanka.

Burt (1926) described *Hypochnusrhacodium* Berk. & M.A. Curtis ex Burt from a US specimen. He wrote of it as having a crust-like and brittle texture, fuscous to dusky drab appearance and spores measuring 6–7 µm in diameter.

Following the original descriptions of *A.phylacteris*, *T.biennis*, *T.umbrina*, *H.tristis*, *H.fuscata*, *H.sitnensis*, *T.arachnoidea* and *H.rhacodium*, a large number of publications were made, proposing new combinations and synonymisations (Gmelin 1792, de Candolle 1815, Fries 1821, 1849, 1874, Quélet 1888, Saccardo 1888, Bresadola 1903, 1916, von Höhnel and Litschauer 1906, 1908, Burt 1916, Donk 1933, Litschauer 1933, Rogers 1948, Parker-Rhodes 1956, Svrček 1958).

No new, morphologically similar species were published until 1967, when Larsen, based on a basidiome he collected in USA, described *Pseudotomentellalongisterigmata* M.J.Larsen – a fungus with a greyish-green hymenium and unusually long sterigmata. In his description of *P.longisterigmata*, Larsen also combined *Thelephoraumbrina* to *Pseudotomentella*. He then proceeded to synonymise all other species similar to *P.umbrina* described thus far (Larsen 1968). Hjortstam (1969), however, argued that *Thelephoraumbrina* and *Hypochnustristis* represented different species that could be separated mainly based on the colour and texture of basidiomata: *T.umbrina* could be recognised by its pale to dark chocolate brown hymenium and softer texture and *H.tristis* could be distinguished by its dark greenish-blue, sometimes brownish-tinted hymenium and firmer texture. Larsen (1971a) disagreed: writing that he had observed a continuum of variation between the distinct character states defined by Hjortstam as characteristic of the two taxa, he considered them as one. Larsen (1971a) further proposed that the taxon in question should have the species epithet *tristis* instead of *umbrina*, with reference to his interpretation of Fries’s (1828) original description and to Rogers and Jackson (1943), who claimed *Thelephoratristis* to be a synonym of *Coniophoraolivacea* (Fr.) P.Karst. He consequently made the combination *Pseudotomentellatristis* M.J.Larsen (1971a) and urged that the use of *Thelephoraumbrina* and all its homotypic synonyms be discontinued.

Larsen (1971b) described one additional species, *Pseudotomentellaatrofusca* M.J.Larsen, based on a blackish-brown, soft basidiome with spores 5.5–6.6 µm in diameter, from the US. Kõljalg (1996) considered *P.longisterigmata* to be a synonym of *P.atrofusca*. Beside *P.tristis*, *P.atrofusca* is the only name left in common use today (GBIF 13–08–2018). It is employed for small-spored specimens in both North America and Europe, but is considerably less frequently collected (GBIF 13–08–2018).

Thus, in conclusion, ten names have so far been associated with the *P.tristis* group, as here defined. Of these, only two – *P.tristis* and *P.atrofusca* – remain in use today. *Pseudotomentellatristis* is under the currently employed, morphological delimitation regarded as a common species with a very wide geographic distribution and ecological amplitude. The purpose of the present study is to molecularly delimit species within the *P.tristis* group, describe their morphology and present knowledge on their ecology and geographical distribution – describing new species and designating types as needed.

## Methods

### Taxon sampling and information

We collected specimens of basidiomata extensively throughout Sweden, Norway and Estonia in the period 2010–2017. For the majority of the Swedish and Norwegian specimens, we recorded the vegetation type of each locality, following Fremstad (1997) and Pålsson (1998). We then sorted this information into the habitats “tundra”, “coniferous forest”, “deciduous forest” and “mixed forest” and the soil pH types “low”, “intermediate” and “high”, following Pålsson (1998) and Halvorsen (2015). We also recorded potential hosts of each specimen, as indicated by nearby ectomycorrhiza-forming plants. The Swedish specimens were photographed, weather permitting. We complemented the fresh material by examining all collections identified as *P.tristis*, *P.atrofusca* and *Pseudotomentella* sp. in GB and TU, along with collections identified as *P.tristis* in TUR and H, and relevant type specimens in S, H, BPI and ARIZ. Permission to extract DNA was granted. In addition, we studied Fries’ collection of *Thelephoraumbrina* in situ at UPS. Taxonomic author abbreviations follow IPNI (26–11–2018) and herbarium codes follow Index Herbariorum (Thiers 2018). Abbreviations of journal titles follow BPH Online (26–11–2018) and abbreviations of book titles follow Stafleu and Cowan (1976–1988).

### Morphological data

We studied all specimens macroscopically and at 20× magnification under a dissecting microscope. Photos of micromorphological characters and measurements were made using an Axioskop 2 microscope (Zeiss, Oberkochen, Germany), equipped with an AxioCam MRc camera (Zeiss) at 400× and 1000× magnifications and in the ZEN Blue software (http://www.zeiss.com/microscopy/int/home.html). Measurements were made on dried material, mounted in 3% (potassium hydroxide) KOH and in Melzer’s reagent. We examined a minimum of three specimens per species, whenever the total number of specimens allowed it, and we measured 20–30 micromorphological structures of each type. Measurements were made to the nearest 0.1 µm, except basidial length, which was measured to the nearest µm. As a necessary aid in identification, we present the values recorded both as spans of the lowest to the highest value and as mean values. For the spans, the 5% smallest and largest measurements are denoted in brackets, in the cases where they differed from the remaining 90%. We calculated the mean values for each specimen omitting the 5% tails and values presented for each species are hence a span of such data. Spore measurements include lobes but exclude echinuli and the hilar appendage. We did not measure abnormally large spores, originating from two-sterigmate basidia. Measurements of basidial width were made at the widest part of the tip of the basidia; basidial length excludes sterigmata. We obtained the width of hyphae from unbroken, internodal sections.

**Figure 1.**
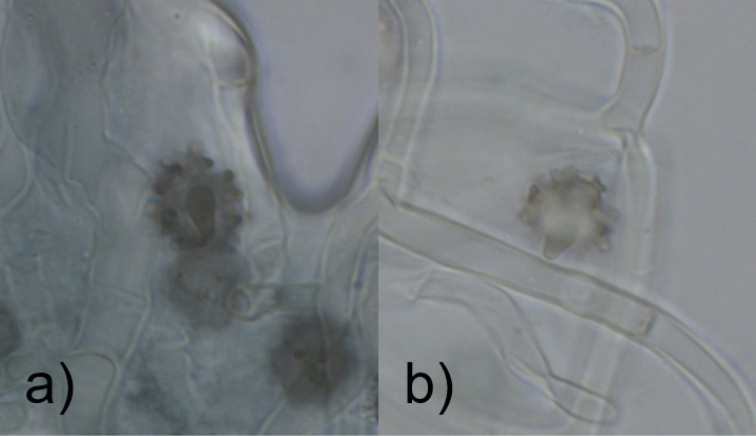
Angle of spore faces. Dorsal side of spores, seen in **a** frontal face and **b** tilted frontal face.

The spore measurements follow Kõljalg (1996) in the recognition of the dorsal side of basidiospores observed in face view as the frontal face (Fig. 1) and the sides of the spores seen in side-view as the lateral faces. Great care has been taken to correctly identify these faces while conducting measurements and not tilted versions of the same, as inclusion of such would undoubtedly lead to an increased margin of error.

For optimal usage of the species descriptions in this article, we recommend readers to utilise the online version, where high resolution pictures are available.

### Molecular data

We generated sequences from four regions for the study: the complete ITS region, including the 5.8S gene, and about 1200 bases of the 5´end of the LSU nuclear ribosomal DNA; about 600 bases of translation elongation factor subunit 1 alpha (Tef1α); and approximately 500 bases of the mtSSU. DNA extractions, PCR reactions and sequencing were performed as described in Larsson et al. (2018). The primers used to amplify the complete ITS region and the 5´end of the LSU region were ITS1F (Gardes and Bruns 1993), LR21, LR0R and LR7 (Hopple and Vilgalys 1999); for Tef1α we used EF983F and EF1567R (Rehner and Buckley 2005); and for mtSSU we used MS1 and MS2 (White et al. 1990). Primers used for sequencing were ITS1, ITS4, MS1, MS2 (White et al. 1990), Ctb6 (https://nature.berkeley.edu/brunslab/tour/primers.html), Lr5 (Hopple and Vilgalys 1999), EF983F and EF1567R.

To assemble the DNA sequences, we used Sequencher 5.1 (Gene Codes, Ann Arbor, MI, USA). We aligned them in AliView 1.18 (Larsson 2014), utilising the L-INS-i strategy as implemented in MAFFT v. 7.017 (Katoh and Standley 2013) and manually adjusted the resulting multiple sequence alignments. Only a few sequences in the Tef1α dataset contained introns. They proved unalignable between species and were removed. In addition, we complemented the nrDNA dataset with representatives of all ITS genotypes belonging to the same 3% Species Hypothesis in the UNITE database (Kõljalg et al. 2005, Parrent and Vilgalys 2007, Peintner et al. 2007, Bidartondo and Read 2008, Krpata et al. 2008, Obase et al. 2009, Tedersoo et al. 2009, Bacher et al. 2010, Cox et al. 2010, Benucci et al. 2011, Obase et al. 2011, Huang et al. 2012, Kranabetter et al. 2012, Sun et al. 2012, Tĕšitelová et al. 2012, LeDuc et al. 2013, Leonardi et al. 2013, Põlme et al. 2013, Tedersoo et al. 2013, Malysheva et al. 2014, Taylor et al. 2014, Guichon 2015, Malysheva et al. 2016, Argüelles-Moyao et al. 2017, Rosenthal et al. 2017, Nilsson et al. 2019). The sequences generated for this article were deposited in GenBank, with accession numbers MK290647–MK290732 and MK312643–MK312663 (Table 1). The specimens they originate from are indicated with an asterisk (*) in the lists of examined specimens.

**Table 1.** DNA regions included per analysis and collection. Accession numbers of DNA sequences generated for this study start with “MK” and for sequences obtained from UNITE with “UDB”; all other sequences were acquired from GenBank. Type collections are shown in boldface. The respective gene trees included all available sequences, whereas collections and accession numbers whose sequences were included in the STACEY analysis are marked with * and those included in both the STACEY and ASTRAL analyses with **.

Species	Collection no.	Country of origin	Acc. no. ITS	Acc. no. LSU	Acc. no. mtSSU	Acc. no. Tef1α
P.abundiloba**	**O F110312**	Norway	MK290731	MK290731	MK290669	MK312646
P.abundiloba*	TU 110852	Estonia	UDB014123			
P.alnophila**	**O F110313**	Norway	MK290715	MK290715	MK290661	
P.alnophila*	–	China	UDB012458			
* P. alnophila *	–	China	UDB012511			
P.alobata**	**O F110315**	Norway	MK290695	MK290695	MK290665	MK312657
P.alobata**	SS425	Sweden	MK290696	MK290696	MK290664	MK312658
* P. alobata *	KHL11873	Sweden	MK290693			
* P. alobata *	O F110316	Norway	MK290694			
* P. alobata *	TU 115626	Slovenia	UDB020318			
P.atrofusca**	**ML7553**	USA	MK290732		MK290651	
P.atrofusca*	–	China	HQ850125			
* P. atrofusca *	–	China	HQ850126			
* P. atrofusca *	–	China	HQ850127			
P.media**	**TU115609**	Estonia	MK290714	MK290714	MK290653	
P.media*	–	Canada	KC840631			
* P. media *	TU 115608	Estonia	UDB016437			
* P. media *	–	Italy	HM044465			
* P. media *	–	Italy	HM044464			
* P. media *	–	Russia	UDB007475			
P.pinophila**	**SS358**	Sweden	MK290708	MK290708	MK290654	
P.pinophila**	SS419	Sweden	MK290710		MK290655	MK312655
* P. pinophila *	O F110328	Norway	MK290709	MK290709		
* P. pinophila *	SS440	Sweden	MK290711			
* P. pinophila *	SS418	Sweden	MK290712			
* P. pinophila *	O F110330	Norway	MK290713			
* P. pinophila *	–	R. o. Korea	AB506089			
* P. pinophila *	–	China	AB636446			
* P. pinophila *	–	R. o. Korea	AB587761			
P.pluriloba**	**US 4263**	Finland	MK290698	MK290698	MK290672	MK312650
P.pluriloba**	SS439	Sweden	MK290699	MK290699	MK290671	MK312649
* P. pluriloba *	–	USA	KF617867			
* P. pluriloba *	–	Canada	JN652992			
P.rotundispora**	**SS413**	Sweden	MK290674	MK290674	MK290657	MK312651
P.rotundispora**	SS394	Sweden	MK290728	MK290728	MK290656	
* P. rotundispora *	SS393	Sweden	MK290729			
* P. rotundispora *	KHL17682	Norway	MK290730			
* P. rotundispora *	TU100138	Estonia	MK290727			
* P. rotundispora *	–	UK	EU668195			
* P. rotundispora *	–	Italy	DQ990858			
* P. rotundispora *	–	Italy	JX625330			
* P. rotundispora *	–	Austria	EF644141			
P.sciastra**	**SS359**	Sweden	MK290686		MK290666	MK312662
P.sciastra**	SS420	Sweden	MK290689		MK290667	MK312661
P.sciastra**	SS312	Sweden	MK290687			MK312663
* P. sciastra *	O F110317	Norway	MK290684	MK290684		
* P. sciastra *	O F110318	Norway	MK290688	MK290688		
* P. sciastra *	TU 124213	Estonia	UDB028204	UDB028204		
* P. sciastra *	TU 124211	Estonia	UDB028202	UDB028202		
* P. sciastra *	TU 110153	Turkey	UDB004970	UDB004970		
* P. sciastra *	O F110322	Norway	MK290685			
* P. sciastra *	SS423	Sweden	MK290690			
* P. sciastra *	KHL17308b	Sweden	MK290691			
* P. sciastra *	TAA 187322	UK	UDB001616			
* P. sciastra *	TU 110113	Turkey	UDB004951			
* P. sciastra *	TU 100644	Estonia	UDB016813			
* P. sciastra *	–	USA	KP814390			
* P. sciastra *	–	USA	EF619790			
P.tristis**	**SS193**	Sweden	MK290679	MK290679	MK290662	
P.tristis**	LK 54/13	Finland	MK290683		MK290663	MK312659
* P. tristis *	KHL15084	Norway	MK290682	MK290682		
* P. tristis *	O F110300	Norway	MK290676	MK290676		
* P. tristis *	TU108134	Estonia	MK290677			
* P. tristis *	O F110297	Norway	MK290678			
* P. tristis *	O F110298	Norway	MK290680			
* P. tristis *	KHL16367	Norway	MK290681			
* P. tristis *	TAAM 159485	Estonia	AF274771			
* P. tristis *	TU 115642	Slovenia	UDB020327			
* P. tristis *	TU 115439	Estonia	UDB016304			
* P. tristoides *	**O F110306**	Norway	MK290692	MK290692		
* P. tristoides *	–	Estonia	UDB008832			
* P. tristoides *	–	Czechia	GU327494			
P.umbrina**	**SS351**	Sweden	MK290700	MK290700	MK290659	MK312654
P.umbrina**	SS239	Sweden	MK290704		MK290660	
P.umbrina**	SS221	Norway	MK290703			MK312653
* P. umbrina *	O F110268	Norway	MK290702	MK290702		
* P. umbrina *	O F110296	Norway	MK290701			
* P. umbrina *	SS280	Sweden	MK290705			
* P. umbrina *	SS174	Sweden	MK290706			
* P. umbrina *	TU 115344	Finland	UDB011636			
* P. umbrina *	TU 115209	Norway	AF274772			
* P. umbrina *	TU 108084	Canada	UDB015056			
* P. umbrina *	–	Denmark	AJ889979			
* P. umbrina *	–	USA	FJ803973			
P.umbrinascens**	**SS335**	Sweden	MK290697	MK290697	MK290670	MK312647
P.umbrinascens*	–	Italy	HM370480			
* P. umbrinascens *	–	Italy	HM370468			
*P.* sp. 1**	SS285	Sweden	MK290716	MK290716	MK290658	MK312652
*P.* sp. 1*	–	Mexico	KF041350			
*P.* sp. 1	–	Russia	KJ769286			
*P.* sp. 1	–	Russia	KP783455			
*P.* sp. 2**	SS169	Sweden	MK290707	MK290707	MK290668	MK312648
*P.* sp. 3	–	Estonia	UDB002898			
*P.* sp. 3	–	Estonia	UDB002899			
P.flavovirens**	KHL17461	Finland	MK290722		MK290648	MK312644
* P. flavovirens *	KHL16310	Sweden	MK290723	MK290723		
P.griseopergamacea**	LLSS883	Norway	MK290721	MK290721	MK290649	
* P. griseopergamacea *	SS401	Sweden	MK290720			
P.humicola**	SS345	Sweden	MK290724	MK290724	MK290650	MK312643
* P. humicola *	SS212	Sweden	MK290675	MK290675		
P.mucidula**	LLSS1155	Norway	MK290725		MK290673	MK312656
* P. mucidula *	LLSS1123	Norway	MK290726	MK290726		
P.nigra**	KHL16273	Finland	MK290718	MK290718	MK290647	MK312645
* P. nigra *	LLSS838	Norway	MK290719	MK290719		
P.rhizopunctata**	SS129	Sweden	MK290717	MK290717	MK290652	
* P. rhizopunctata *	–	Canada	KP889924			
P.vepallidospora**	TU 115205	Norway	UDB000278	UDB000278		
* P. vepallidospora *	–	Germany	HM146848			

The species described in this article have been provided with links to the UNITE Species Hypotheses they are part of, in the cases where such exist. Upon publication of the article, the Species Hypotheses will be updated with their new names and the DNA sequences generated for the article will be made available in GenBank. At the next update of UNITE, the ITS sequences will then be copied from GenBank and clustered into the appropriate Species Hypotheses.

### Molecular analyses

We used SplitsTree 4.14.4 (Huson and Bryant 2006) to explore the amount of intragenic conflict and possible presence of intragenic recombination, and RDP4 (Martin et al. 2015) to test for recombination. In RDP, all DNA regions were initially submitted to testing with the methods RDP, GENECONV, Chimaera and MaxChi, with Bonferroni correction and 0.01 as the significance level. We submitted sequences with significant signs of recombination to a second round of testing that made use of all recombination methods. Sequences with a positive result for more than two methods with p-values ≤ 10^-5^ in the second round were regarded as probable recombinants.

To generate Bayesian phylogenetic trees from the alignments, we used BEAST 2.4.7 (Bouckaert et al. 2014), employing the standard version of the programme for gene tree estimation, and STACEY 1.2.4 (Jones 2017) for species tree inference under the multispecies coalescent model. We prepared the xml-files for the BEAST 2 runs in BEAUti 2.4.7 (Bouckaert et al. 2014). The following minimal partitions were assumed per unlinked genetic region (Table 2): ITS1, 5.8S, ITS2, LSU (nrDNA); Tef1α first positions, Tef1α second positions, Tef1α third positions (Tef1α); mtSSU (mtSSU). We used the automated best-fit tests implemented in PAUP 4.0a (Swofford 2002) to select optimal substitution models and substitution model partitions for each minimal partition. Using three substitution schemes, the following partitions and models had the highest ranking, according to BIC scores: ITS1+ITS2 (HKY+I+G), 5.8S+LSU (GTR+I+G), Tef1α first positions (JC+I), Tef1α second positions (K80+I), Tef1α third positions (HKY) and mtSSU (GTR+G). The BEAST 2 and STACEY analyses did not converge under the GTR model and invariant site fraction parameter (I), however, such that we used HKY+G for the two nrDNA substitution model partitions and the mtSSU region. Omitting the I parameter in a rerun of the partition test for Tef1α yielded the result JC+G for first+second positions and HKY for the third positions and these were hence the partitions and models we adopted in the BEAST 2 analyses.

**Table 2.** Partitions and models used in the STACEY analysis.

DNA region	Minimal partitions	Substitution model partitions	Substi-tution model	Clock model partitions	Clock model	Tree-estimation partitions
nrDNA	ITS1	ITS1+ITS2	HKY+G	ITS1	Lognormal, relaxed	ITS1+5.8S+ITS2+LSU
	5.8S	5.8S+LSU	HKY+G	5.8S	Lognormal, relaxed	ITS1+5.8S+ITS2+LSU
	ITS2	ITS1+ITS2	HKY+G	ITS2	Lognormal, relaxed	ITS1+5.8S+ITS2+LSU
	LSU	5.8S+LSU	HKY+G	LSU	Lognormal, relaxed	ITS1+5.8S+ITS2+LSU
Tef1α	Tef1α 2nd pos.	Tef1α 1st pos.+ Tef1α 2nd pos.	JC+G	Tef1α 2nd pos.	Lognormal, relaxed	Tef1α 1st pos.+ Tef1α 2nd pos.+ Tef1α 3rd pos.
	Tef1α 2nd pos.	Tef1α 1st pos.+ Tef1α 2nd pos.	JC+G	Tef1α 2nd pos.	Lognormal, relaxed	Tef1α 1st pos.+ Tef1α 2nd pos.+ Tef1α 3rd pos.
	Tef1α 3rd pos.	Tef1α 3rd pos.	HKY	Tef1α 3rd pos.	Lognormal, relaxed	Tef1α 1st pos.+ Tef1α 2nd pos.+ Tef1α 3rd pos.
mtSSU	mtSSU	mtSSU	HKY+G	mtSSU	Lognormal, relaxed	mtSSU

The substitution rate of each partition was estimated independently of the others in each BEAST 2 run. We set all individuals as separate species in the STACEY analysis. We set the trees of the minimal nrDNA partitions as linked, as did we for the Tef1α minimal partitions. We set the clock models of all minimal partitions as unlinked and a lognormal, relaxed clock model was assumed for each, as test runs had shown that all partitions had a coefficient of variation well above 0.1 (i.e. implying a relatively high rate variation amongst branches). The clock rate of each partition was estimated in the runs, using a lognormal prior, with a mean set to one in real space. We set the growth rate prior to lognormal, with a mean of 5 and a standard deviation of 2. The Collapse Height prior of the STACEY analysis was set to 10^-5^ and a lognormal prior with a mean of -7 and a standard deviation of 2 was set to the PopPriorScale parameter.

We ran the Markov Chain Monte Carlo (MCMC) chains of the mtSSU and Tef1α regions for 10 million generations with tree and parameter files sampled every 1000 generations. For the nrDNA and STACEY analysis, we ran the MCMC chains for 100 million generations and sampled it every 5000 generations, and for 1 billion generations and sampled it every 25000 generations, respectively. All analyses converged well in advance of the 10% burn-in threshold and had effective sampling size values well above 200 for all parameters. Chain mixing was found to be satisfactory as assessed in Tracer 1.6.0 (Rambaut et al. 2014). After discarding the burn-in trees, maximum clade credibility trees were identified by TreeAnnotator 2.4.7 (Bouckaert et al. 2014). Posterior probabilities of the clusterings of the species trees output by STACEY were analysed in the associated software SpeciesDelimitationAnalyser version 1.8.0, with burn-in set to 10%, simcutoff to 1 and collapseheight to 10^-5^. The estimated similarity matrix was visualised by the R-script plot.simmatrix.R (Jones et al. 2015).

To generate Maximum Likelihood (ML) gene trees, we used PhyML 3.1 (Guindon et al. 2010). We set the substitution model to GTR+I+G for the Tef1α, nrDNA and mtSSU regions, since it was the best-fit model output by the automated model test in PAUP, using AICc and three substitution schemes. The tree topology search was conducted using NNI+SPR, with ten random starting trees. Non-parametric bootstrap analyses with 1000 replicates were performed on the resulting trees. We also inferred a species tree from the ML gene trees, using ASTRAL III (Zhang et al. 2018), with node support calculated as local posterior probabilities (Sayyari and Mirarab 2016).

The Bayesian and ML gene trees comprised the entire nrDNA, mtSSU and Tef1α datasets of this study, while the STACEY and ASTRAL species trees, including the ML trees underlying the latter, contained subsets thereof (Table 1) to avoid destabilising the analyses with large amounts of missing data.

We visually prepared the resulting trees from the Bayesian and ML analyses in FigTree 1.4.3 (Rambaut 2012) and Inkscape (Albert et al. 2018).

## Results

### Molecular species delimitation

**Figure 2.**
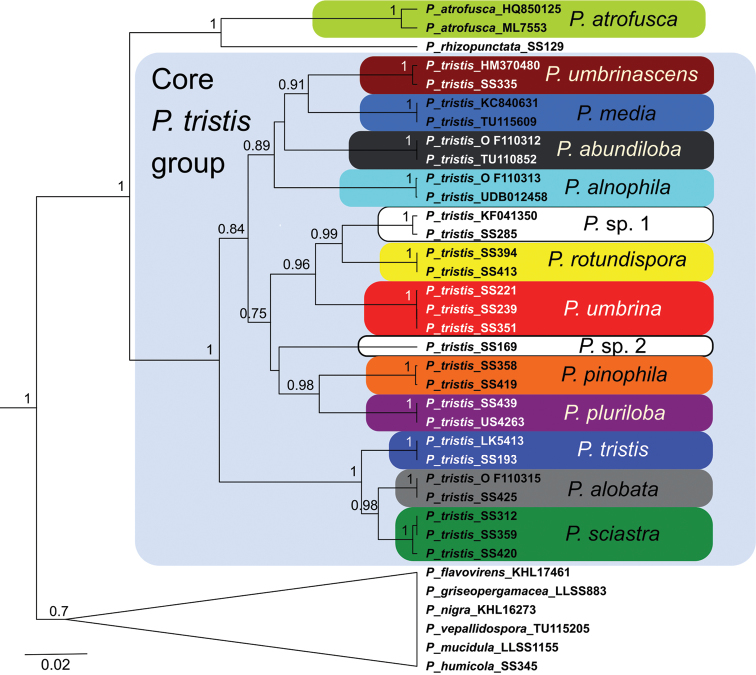
STACEY species tree of the *P.tristis* group. Numbers at nodes denote posterior probability values (only values > 0.70 are shown). The branch lengths are scaled in estimated number of substitutions/site.

The STACEY analysis retrieved 13 well-supported clades, based on DNA from specimens morphologically belonging to the *P.tristis* group. We interpret these as species (Fig. 2). The corresponding clades in the ASTRAL analysis were also supported, when present as more than one leaf node (Fig. 4). We found three of the delimited species, *P.tristis*, *P.umbrina* and *P.atrofusca*, to constitute previously described taxa, while nine species are described as new to science: *P.sciastra*, *P.umbrinascens*, *P.pinophila*, *P.alnophila*, *P.alobata*, *P.pluriloba*, *P.abundiloba*, *P.rotundispora* and *P.media*. We chose not to describe *P.* sp. 1, since the only collection available is too small to be suitable as a type. The same applies to *P.* sp. 2, which in addition was retrieved as a singleton by both analyses.

**Figure 3.**
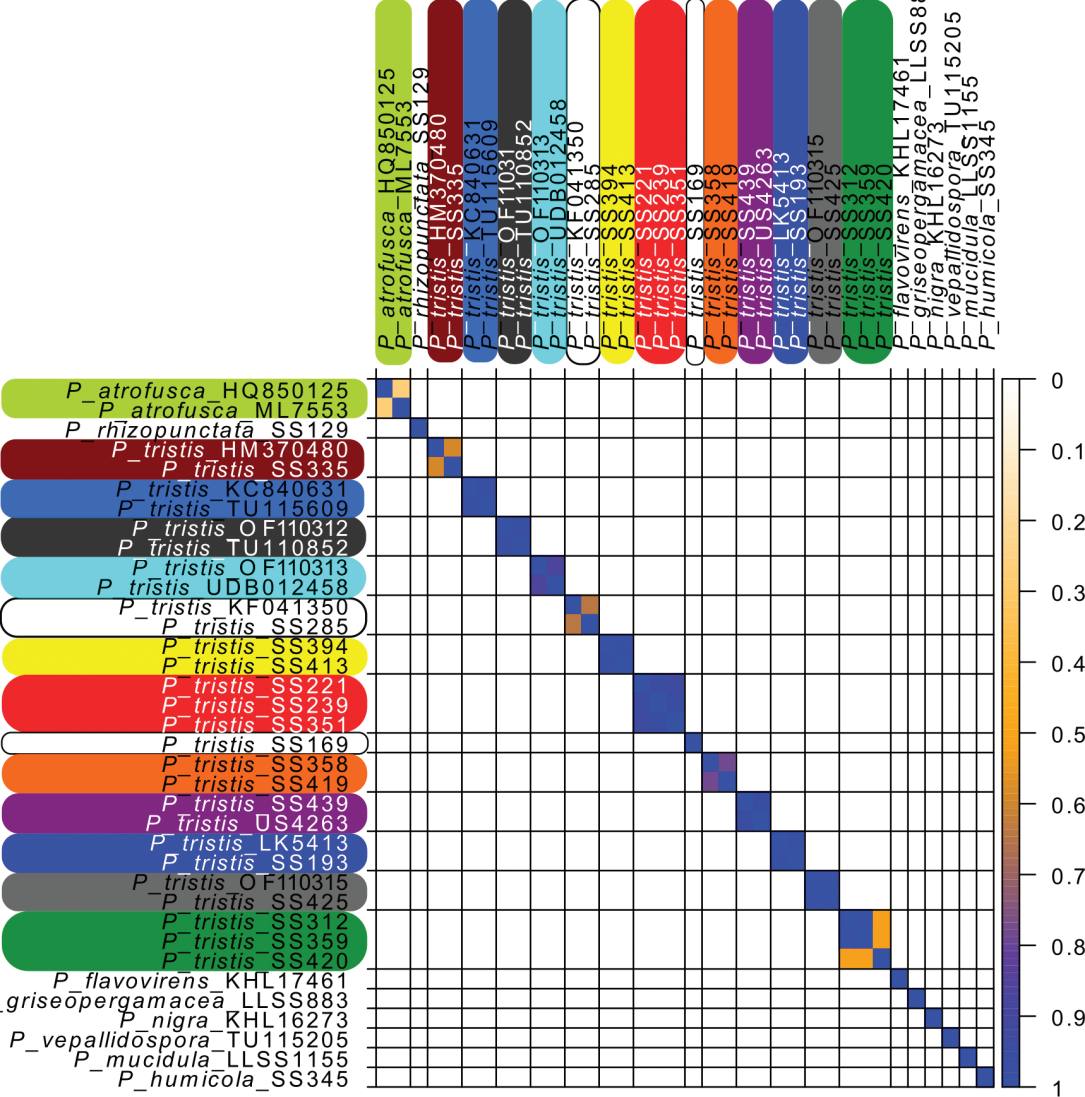
Pairwise similarity matrix between the clusters of the STACEY species tree. The species are colour-coded the same as in Fig. 2. Values between 0 and 1 denote posterior probability.

The delimitation of the species recognised in the species tree equals the clusters output by SpeciesDelimitationAnalyser (Fig. 3). As displayed by the similarity matrix, each cluster has internal support and zero posterior probability of the included individuals belonging to any other cluster. In the case of specimens SS419 and SS420 and sequences HQ850125, HM370480, UDB012458 and KF041350, however, the similarity matrix shows weak support for them to belong to their respective clusters; but given the strong support for the corresponding species in the species tree, we interpret these as instances of intraspecific genetic structure.

**Figure 4.**
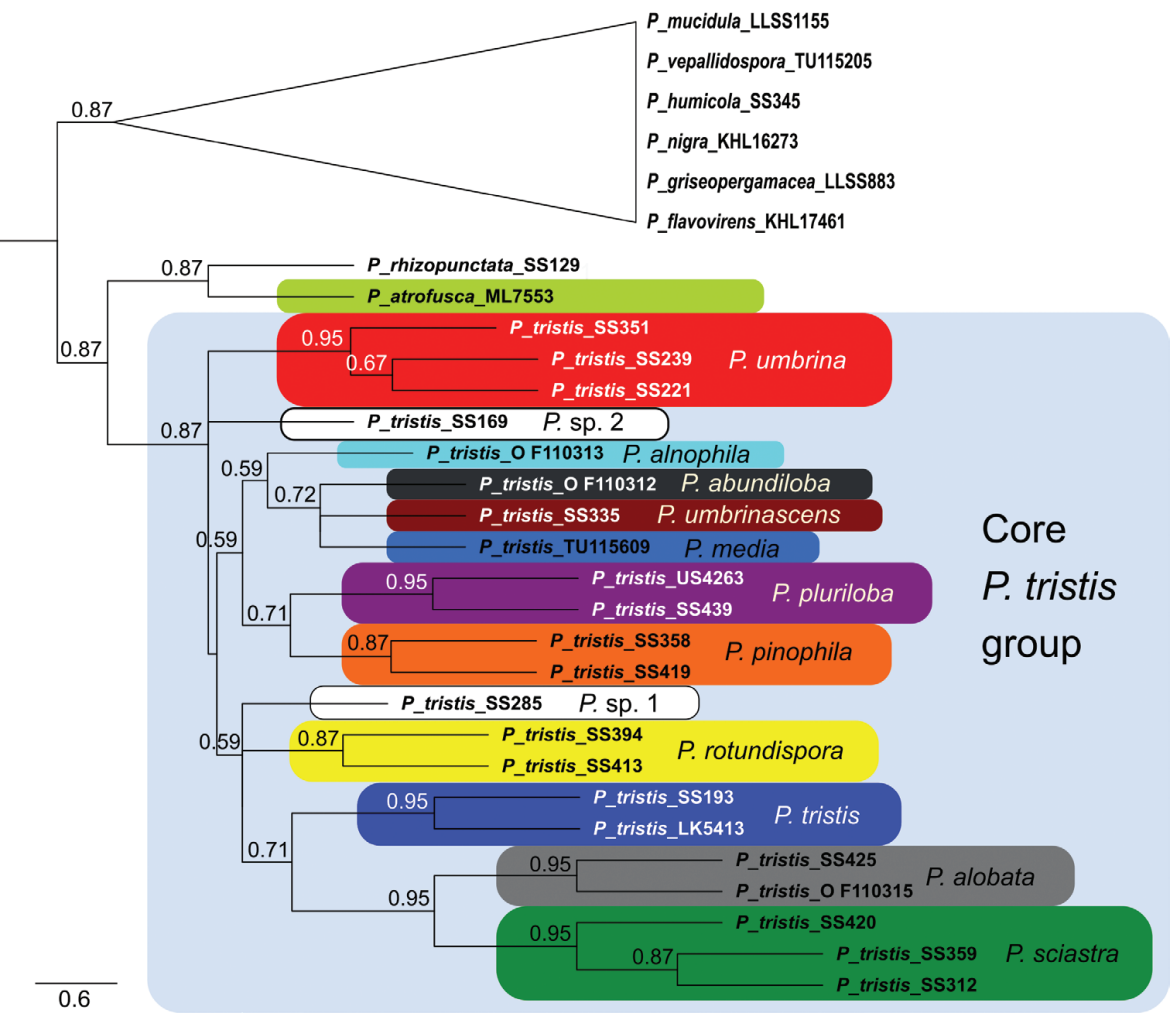
ASTRAL species tree of the *P.tristis* group. Numbers between 0.5 and 1 denote local posterior probability values (only values > 0.5 are shown). The internal branch lengths are scaled in coalescent units, while the length of the terminal branches is a standard value set by the programme.

**Figure 5.**
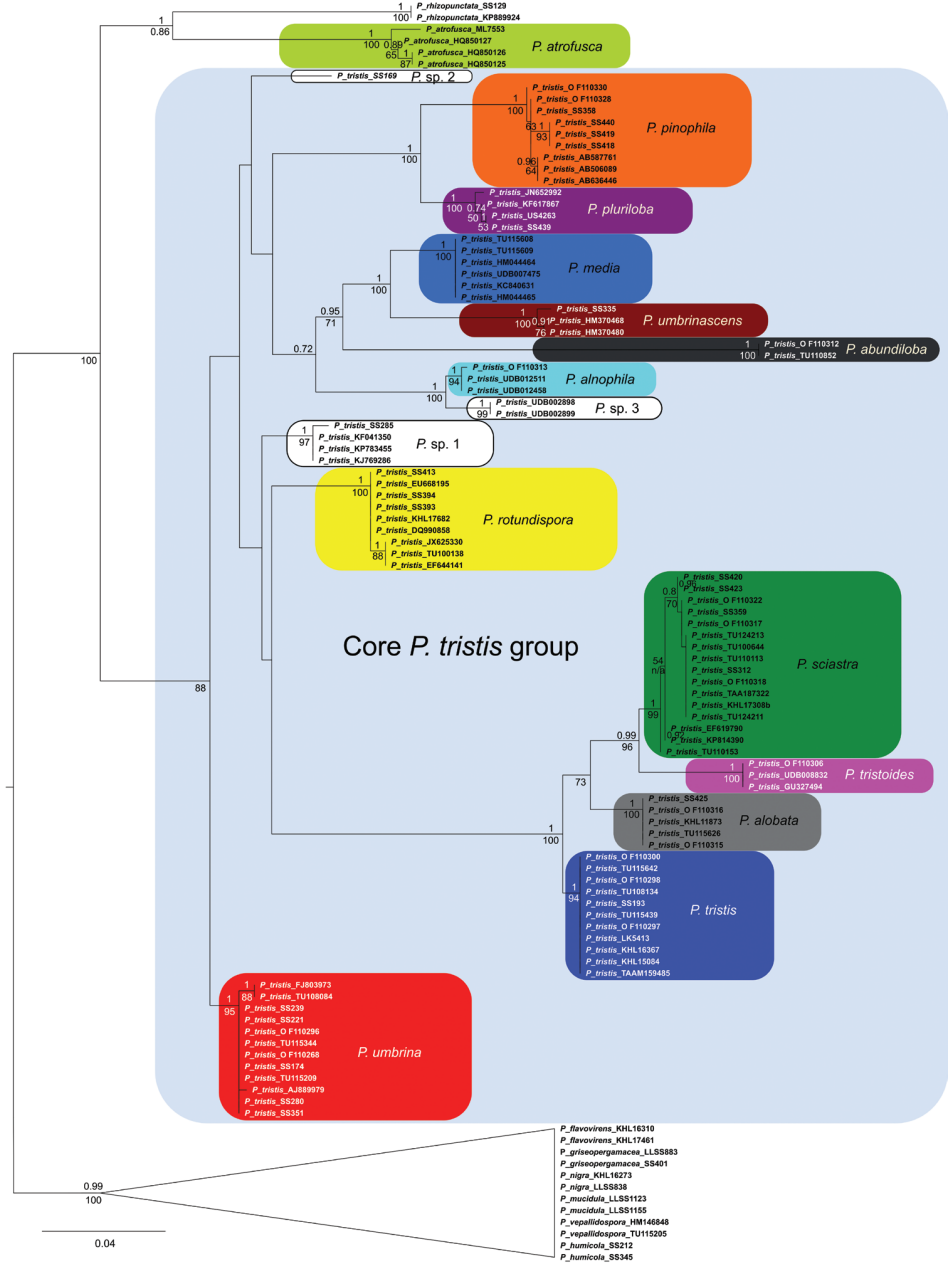
Nuclear ribosomal DNA phylogeny of the *P.tristisgroup*. ML phylogram with bootstrap support values (only values > 50 are shown), and posterior probability values added from congruent Bayesian tree (only values > 0.7 are shown). Branch lengths are scaled in substitutions/site.

In addition to the species delimited based on clades in the species tree, we recognised two species, *P.tristoides* and *P.* sp. 3, based on their presence as highly supported nodes in the nrDNA gene tree (Fig. 5). Their sequences could not be included in the species tree analyses, due to lack of data for the other genetic regions. We did not describe *P.* sp. 3, since it has no physical material tied to it.

### Phylogenetic relationships

The species tree analyses were congruent. The trees retrieved show that the species in the *P.tristis* group, with the addition of *Pseudotomentellarhizopunctata* E.C.Martini & Hentic, form a monophyletic clade with high support (Figs 2, 4). Its two daughter clades, one containing *P.rhizopunctata* and *P.atrofusca* and the other containing the remaining species of the *P.tristis* group (the “core *P.tristis* group”), were also well supported. The phylogenetic relationships within the core *P.tristis* group, however, were not; only the clades (*P.sciastra*, *P.alobata*), ((*P.sciastra*, *P.alobata*), *P.tristis*) and (*P.pinophila*, *P.pluriloba*) were supported by both analyses. In addition, the clades ((*P.rotundispora*, *P.* sp. 1), *P.umbrina*) and (*P.rotundispora*, *P.* sp. 1) were supported by the STACEY analysis.

No signal of intragenic recombination was detected in RDP4, but as indicated by the low phylogenetic resolution present also in the gene trees (Fig. 5, Suppl material 1: Figs S1–S4) and the network-like structure between splits observed in SplitsTree (Suppl material 1: Figs S5–S7) for the included genetic regions, there is considerable intragenic conflict between species. The exception to this pattern is the highly supported, long branch of the clades (((*P.sciastra*, *P.tristoides*), *P.alobata*), *P.tristis*) and ((*P.sciastra*, *P.alobata*), *P.tristis*) in the nrDNA and Tef1α trees, respectively. In the neighbour nets of the nrDNA and Tef1α regions, this clade is reminiscent of the “dog-bone” shape displayed by paralogy, a hypothesis that is reinforced by its placement at the very root of the Tef1α tree. The branch in question thus constitutes the only – but in itself a major – incongruence between the gene trees.

A methodological observation to future users of STACEY with limited amounts of data is that support for species-level nodes decreases dramatically unless at least one included leaf taxon has a complete coverage of all the genetic regions used.

### Type studies

All of the 13 newly described and previously described species can be distinguished morphologically (Table 3), although the differences exhibited by some species pairs are small. We found the previously designated lectotype of *P.tristis* and neotype of *P.umbrina* to fall within the morphological variation of sequenced material, with which they could hence be epitypified. The lectotypes of *H.fuscata* and *H.sitnensis*, however, display the morphological characteristics of *P.tristis*, with which we thus considered them conspecific. Of the seven type specimens studied, we were only able to generate ITS sequence data for *P.atrofusca*. The European collections, studied of *P.atrofusca*, all belong to *P.sciastra*.

**Table 3.** The most taxonomically informative micromorphological characters. Summary statistics for each species is marked in bold. “Frontal” and “lateral” refer to the corresponding spore faces and “L” and “W” denote mean length and width, respectively. All measurements are in µm, with the 5% smallest and largest values denoted in brackets, when differing from the remaining 90%.

Species/ collection	Frontal length	Frontal L	Frontal width	Frontal W	Lateral length	Lateral L	Lateral width	Lateral W	Length echinuli	L echinuli	Width subic. hyphae	W subic. hyphae
***P.abundiloba* sp. nov.**	**(8.8–) 9.2–10.5**	**9.8**	**(8.0–) 8.6–10.7 (–10.8)**	**9.5–9.6**	**(8.9–) 9.3–10.1 (–10.5)**	**9.7–9.8**	**(6.7–) 7.0–8.1 (–8.2)**	**7.3–7.7**	**(0.9–) 1.1–1.8 (–1.9)**	**1.2–1.5**	**(4.3–) 4.8–6.9 (–7.2)**	**5.5–6.1**
holotype	(8.8–) 9.2–10.5	9.8	(8.0–) 8.6–10.7 (–10.8)	9.6	(9.2–) 9.3–10.1 (–10.2)	9.7	(6.7–) 7.0–7.7 (–7.8)	7.3	(0.9–) 1.1–1.8 (–1.9)	1.5	(5.0–) 5.5–6.9 (–7.2)	6.1
TU 110852	(9.0–) 9.3–10.2	9.8	(8.9–) 9.0–10.2 (–10.3)	9.5	(8.9–) 9.3–10.1 (–10.5)	9.8	(7.0–) 7.4–8.1 (–8.2)	7.7	1.0–1.7	1.2	(4.3–) 4.8–6.2	5.5
***P.alnophila* sp. nov. holotype**	**(8.8–) 9.0–10.1 (–10.4)**	**9.5**	**9.2–10.2 (–10.6)**	**9.8**	**9.0–10.6**	**9.6**	**(6.6–) 6.9–8.1 (–8.2)**	**7.7**	**(0.8–) 0.9–1.7**	**1.2**	**4.0–5.0 (–5.1)**	**4.5**
***P.alobata* sp. nov.**	**(9.0–) 9.1–10.7**	**9.7–10.1**	**(8.4–) 8.9–10.5 (–10.7)**	**9.5–9.8**	**(8.9–) 9.1–10.3**	**9.7–9.9**	**(6.5–) 6.7–8.2**	**7.1–7.4**	**1.2–1.8 (–1.9)**	**1.4–1.7**	**(4.3–) 4.6–7.4 (–7.6)**	**5.6–5.9**
holotype	9.5–10.7	10.1	(9.1–) 9.2–10.4 (–10.7)	9.8	(9.6–) 9.7–10.1	9.9	6.8–8.2	7.4	1.2–1.8	1.4	(4.7–) 5.0–6.9	5.9
TU 115626	(9.0–) 9.3–10.5 (–10.6)	9.8	(8.8–) 9.0–10.5	9.5	(8.9–) 9.1–10.3	9.7	(6.7–) 6.8–7.7	7.2	(1.2–) 1.3–1.8 (–1.9)	1.6	(4.3–) 4.6–7.4 (–7.6)	5.8
O F110316	(9.0–) 9.1–10.4	9.7	(8.4–) 8.9–10.3 (–10.5)	9.5	9.5–10.1 (–10.3)	9.8	(6.5–) 6.7–7.5 (–7.6)	7.1	(1.4–) 1.6–1.8 (–1.9)	1.7	(4.7–) 4.8–6.3 (–6.8)	5.6
***P.atrofusca* holotype**	**(6.1–) 6.2–7.0 (–7.1)**	**6.6**	**(5.8–) 6.3–7.2 (–7.3)**	**6.8**	**6.3–6.9 (–7.3)**	**6.5**	**(4.0–) 4.1–4.8 (–5.0)**	**4.4**	**0.6–0.9 (–1.1)**	**0.8**	**(1.7–) 1.8–2.8**	**2.3**
***P.longisterigmata* holotype**	**(9.7–) 10.0–11.7**	**11.0**	**(9.4–) 9.8–11.7**	**10.7**	**10.3–11.5 (–11.7)**	**10.9**	**(6.7–) 7.5–9.1**	**8.5**	**1.2–1.8 (–2.1)**	**1.5**	**4.9–7.2**	**6.2**
***P.media* sp. nov.**	**(7.8–) 8.0–9.5**	**8.9–9.3**	**(8.3–) 8.5–9.9 (–10.1)**	**9.2–9.8**	**(8.8–) 8.9–9.6**	**9.1–9.4**	**(6.6–) 7.0–7.9**	**7.3–7.6**	**(0.8–) 0.9–1.6**	**1.1–1.2**	**(3.6–) 3.7–5.0 (–5.4)**	**4.1–4.6**
holotype	(8.5–) 8.7–9.5	9.3	(9.0–) 9.1–9.9	9.8	(8.8–) 8.9–9.6	9.4	7.0–7.7 (–7.8)	7.3	(0.8–) 0.9–1.3 (–1.4)	1.1	(3.6–) 3.7–4.6	4.1
TU 115608	(7.8–) 8.0–9.4 (–9.5)	8.9	(8.3–) 8.5–9.9 (–10.1)	9.2	8.9–9.4 (–9.6)	9.1	(6.6–) 7.2–7.9	7.6	(0.8–) 0.9–1.6	1.2	(3.8–) 4.4–5.0 (–5.4)	4.6
***P.pinophila* sp. nov.**	**(7.7–) 7.9–10.2 (–10.3)**	**8.6–9.1**	**(7.7–) 8.3–10.1 (–10.2)**	**8.8–9.4**	**(8.2–) 8.3–9.7 (–9.8)**	**8.7–9.0**	**(5.7–) 5.8–6.8 (–7.0)**	**6.3–6.6**	**(0.6–) 0.8–1.4 (–1.5)**	**0.9–1.1**	**3.0–4.9**	**3.6–4.1**
holotype	(7.7–) 7.9–10.2 (–10.3)	9.1	8.3–10.1 (–10.2)	9.4	8.4–9.5 (–9.8)	9.0	(6.0–) 6.1–6.8 (–6.9)	6.3	(0.8–) 0.9–1.4 (–1.5)	1.1	3.2–3.9 (–4.3)	3.6
O F110330	8.0–9.4 (–9.7)	8.7	(8.6–) 8.9–9.9	9.3	(8.5–) 8.6–9.4	8.8	6.1–7.0	6.5	(0.6–) 0.8–1.1	0.9	(3.4–) 3.5–4.9	4.1
O F110305	(8.1–) 8.3–8.9 (–9.4)	8.6	(7.7–) 8.4–9.4 (–9.6)	8.8	(8.2–) 8.3–9.7 (–9.8)	8.7	(5.7–) 5.8–6.8 (–6.9)	6.3	0.8–1.4	1.1	3.0–4.4 (–4.8)	3.7
***P.pluriloba* sp. nov.**	**(9.0–) 9.1–10.8 (–10.9)**	**9.8**	**(9.2–) 9.3–10.9 (–11.1)**	**10.2**	**9.0–10.4 (–10.8)**	**9.6–9.8**	**(6.7–) 6.8–8.5 (8.6)**	**7.5–7.6**	**(0.9–) 1.0–1.9**	**1.4**	**(3.9–) 4.0–5.9 (–6.8)**	**4.8–5.1**
holotype	(9.0–) 9.1–10.4 (–10.8)	9.8	(9.2–) 9.3–10.9 (–11.1)	10.2	9.0–10.4 (–10.8)	9.8	(6.7–) 6.8–8.5 (–8.6)	7.6	(1.0–) 1.1–1.9	1.4	(4.1–) 4.7–5.9 (–6.8)	5.1
SS439	(9.2–) 9.3–10.8 (–10.9)	9.8	9.5–10.9 (–11.0)	10.2	(9.3–) 9.4–9.9 (–10.4)	9.6	6.9–7.9 (–8.1)	7.5	(0.9–) 1.0–1.8 (–1.9)	1.4	(3.9–) 4.0–5.4 (–5.8)	4.8
***P.rhacodia* comb. nov. syntype**	**(7.8–) 8.0–9.1 (–9.3)**	**8.3**	**(7.7–) 7.8–8.9 (–9.0)**	**8.3**	**(7.9–) 8.2–8.9**	**8.5**	**(5.4–) 5.9–6.8 (–7.0)**	**6.3**	**(0.9–) 1.0–1.6 (–1.7)**	**1.3**	**(5.6–) 5.7–7.3 (–8.0)**	**6.5**
***P.rotundispora* sp. nov.**	**(6.7–) 7.0–8.2 (–8.4)**	**7.5–7.6**	**7.0–8.6**	**7.7–7.9**	**7.0–8.2 (–8.3)**	**7.6–7.9**	**(5.2–) 5.3–6.0 (–6.1)**	**5.6–5.7**	**0.5–1.1 (–1.3)**	**0.8**	**3.0–4.4 (–4.6)**	**3.4–3.8**
holotype	(6.7–) 7.0–8.1 (–8.4)	7.6	7.1–8.5 (–8.6)	7.9	(7.1–) 7.2–8.2	7.9	5.5–6.0	5.7	0.7–0.9 (–1.1)	0.8	3.0–4.1 (–4.3)	3.4
SS394	(6.9–) 7.1–8.2 (–8.3)	7.6	(7.0–) 7.3–8.6	7.8	7.0–8.1 (–8.3)	7.7	(5.2–) 5.3–6.0 (–6.1)	5.7	0.5–1.0	0.8	(3.1–) 3.4–4.4 (–4.6)	3.8
SS393	7.0–7.9 (–8.0)	7.5	7.0–8.2 (–8.4)	7.7	7.3–8.2	7.6	5.3–5.8	5.6	0.5–1.1 (–1.3)	0.8	3.1–3.8 (–4.1)	3.5
***P.sciastra* sp. nov.**	**(6.0–) 6.1–7.9 (–8.1)**	**6.6–7.3**	**6.3–8.2**	**6.7–7.7**	**(6.2–) 6.5–7.7 (–8.0)**	**6.8–7.3**	**(4.3–) 4.4–6.0 (–6.2)**	**4.6–5.4**	**(0.5–) 0.6–1.2 (–1.4)**	**0.8–0.9**	**(3.9–) 4.4–6.6 (–6.8)**	**5.0–5.8**
holotype	6.5–7.9 (–8.1)	7.3	(6.8–) 7.0–8.1 (–8.2)	7.7	(6.5–) 6.7–7.7 (–8.0)	7.3	4.7–6.0 (–6.2)	5.4	0.6–1.2 (–1.3)	0.8	(4.5–) 4.8–6.4 (–6.8)	5.7
O F110317	(6.5–) 6.6–7.9 (–8.0)	7.2	(6.9–) 7.0–8.2	7.6	(6.7–) 7.0–7.6 (–7.8)	7.3	4.8–5.9 (–6.1)	5.4	(0.5–) 0.6–1.2 (–1.4)	0.9	(4.7–) 4.9–6.6 (–6.7)	5.8
TAA 187322	(6.0–) 6.1–7.0 (–7.1)	6.6	6.3–7.3 (–7.6)	6.7	(6.2–) 6.5–7.1 (–7.3)	6.8	(4.3–) 4.4–5.6 (–5.7)	4.6	0.6–1.1 (–1.4)	0.8	(3.9–) 4.4–5.8 (–6.0)	5.0
*** P. tristis ***	**7.7–9.1 (–9.2)**	**8.3–8.5**	**8.0–9.3 (–9.6)**	**8.4–8.6**	**7.7–9.0 (–9.1)**	**8.3–8.5**	**5.6–) 6.0–6.8 (–7.0)**	**6.3–6.5**	**(0.8–) 0.9–1.9**	**1.4**	**(4.5–) 4.6–7.4**	**5.4–6.2**
epitype	(7.7–) 8.1–8.8 (–9.0)	8.5	(8.0–) 8.1–9.0	8.6	(7.7–) 8.0–9.0	8.5	(5.6–) 6.1–6.8	6.5	(0.8–) 1.0–1.9	1.4	4.6–6.4 (–6.9)	5.7
lectotype	7.7–8.8	8.3	8.2–9.1	8.6	(7.9–) 8.0–8.8	8.3	6.0–6.7 (–7.0)	6.5	1.1–1.8	1.4	(4.5–) 4.7–7.4	5.9
TAA 159485	(7.8–) 7.9–9.1 (–9.2)	8.4	8.0–8.9 (–9.2)	8.4	8.0–8.7	8.4	6.1–6.7 (–7.0)	6.4	0.9–1.8	1.4	(5.0–) 5.4–6.1 (–6.4)	5.7
L. Kosonen 54/13	8.0–9.1 (–9.2)	8.5	8.1–9.2 (–9.6)	8.6	8.1–8.9	8.4	6.1–6.6	6.3	(0.9–) 1.1–1.7	1.4	(4.6–) 4.7–6.2 (–6.3)	5.4
KHL15084	7.9–9.1	8.3	(8.1–) 8.3–9.3	8.6	7.7–8.9 (–9.1)	8.3	(5.9–) 6.0–6.8	6.4	(0.8–) 1.0–1.8	1.4	(5.2–) 5.3–7.0 (–7.4)	6.2
***H.fuscata* lectotype**	**(7.8–) 8.0–9.0**	**8.5**	**(8.1–) 8.4–9.1**	**8.7**	**(7.9–) 8.1–9.2 (–9.4)**	**8.5**	**6.0–6.5**	**6.3**	**(1.1–) 1.2–1.6 (–1.7)**	**1.4**	**5.4–6.4**	**5.9**
***H.sitnensis* holotype**	**7.9–9.2 (–9.3)**	**8.5**	**(7.7–) 8.1–9.2**	**8.6**	**(7.7–) 8.1–8.9 (–9.2)**	**8.5**	**(5.9–) 6.3–6.9**	**6.5**	**1.0–1.8**	**1.5**	**5.5–6.9 (–7.1)**	**6.1**
***P.tristoides* sp. nov. holotype**	**7.7–8.6 (–8.8)**	**8.2**	**(7.4–) 7.7–9.3 (–9.5)**	**8.5**	**(7.9–) 8.0–8.6**	**8.2**	**6.0–6.5 (–6.7)**	**6.3**	**(0.5–) 0.7–0.9 (–1.1)**	**0.8**	**(4.7–) 4.9–7.1 (–7.6)**	**6.0**
*** P. umbrina ***	**7.7–9.3 (–9.4)**	**8.3–8.7**	**(7.6–) 7.9–9.1 (–9.4)**	**8.4–8.7**	**8.0–9.3 (–9.6)**	**8.4–8.7**	**(5.1–) 5.6–6.7 (–6.9)**	**6.0–6.1**	**(0.7–) 0.8–1.5**	**1.1–1.2**	**3.3–4.8 (–5.3)**	**4.0–4.3**
epitype	(8.0–) 8.4–9.1	8.7	(7.7–) 8.1–9.0 (–9.1)	8.5	(8.0–) 8.2–9.1 (–9.6)	8.6	(5.1–) 5.8–6.7 (–6.8)	6.1	0.9–1.5	1.2	(3.7–) 3.8–4.7 (–4.9)	4.3
neotype	(7.9–) 8.0–9.3 (–9.4)	8.7	8.2–9.1	8.7	8.1–9.3	8.7	(5.4–) 5.8–6.5 (–6.7)	6.0	(0.7–) 0.9–1.4	1.2	3.3–4.7 (–5.3)	4.0
O F110268	7.7–8.9 (–9.0)	8.3	(7.6–) 7.9–9.1 (–9.4)	8.4	8.0–9.1 (–9.5)	8.4	(5.3–) 5.6–6.7 (–6.9)	6.1	0.8–1.3	1.1	3.5–4.8 (–4.9)	4.1
***P.umbrinascens* sp. nov. holotype**	**(8.5–) 8.7–9.4 (–9.6)**	**8.9**	**(8.4–) 8.7–9.2 (–9.3)**	**8.9**	**8.5–9.2 (–9.4)**	**8.9**	**(5.7–) 6.0–6.5**	**6.2**	**(0.9–) 1.0–1.9 (–2.0)**	**1.6**	**3.1–) 3.2–4.3 (–4.8)**	**3.7**

The type collections of *P.longisterigmata* and *H.rhacodium* differ morphologically from all other specimens studied. Their aberrant morphology, with extremely long sterigmata and a very hard and thick basidiome, respectively (see further under “Taxonomy”), however, suggest that they may be misshapen forms of other species.

*Septobasidiumarachnoideum* (Berk. & Broome) Bres. was accepted in *Septobasidium* by the thorough study of Couch (1938) and hence, we consider it excluded from Thelephorales Corner ex Oberw. We designated a plate by Bulliard (1790) as lectotype of *A.phylacteris* and *T.biennis*. The morphology displayed by this plate and stated in the original descriptions of these species does not match any *Pseudotomentella* species known to date (Larsen 1971a, Stalpers 1993, Kõljalg 1996).

### Morphology

We were able to discern a few morphological patterns amongst the clades of the nrDNA trees. The most pronounced is perhaps the lack of hyphal cords and skeletal hyphae in the species of the core *P.tristis* group. These are characters that are present in *P.atrofusca* and *P.rhizopunctata* (Larsen 1971a, Martini and Hentic 2003) and indeed in all other simple-septate *Pseudotomentella* species (Larsen 1971a, Kõljalg 1996). Generally, spore shape and dimensions, the width of subicular hyphae and the length of echinuli proved to be the most taxonomically informative characters. Subhymenial hyphal width, basidial dimensions and sterigmal length were moderately useful for distinguishing between species, whereas the Q value (spore length/width) was considerably less so. The presence of wide subicular hyphae, a blue green reaction in the hymenium and subhymenium and amyloid material present in and on the same, in the species of the clade containing *P.tristis*, *P.alobata*, *P.sciastra* and *P.tristoides*, is also worthy of notice. These are, however, characters also present in species of other clades, e.g. (*P.pinophila*, *P.pluriloba*) and (*P.umbrina*, *P.rotundispora*), where they are not shared amongst all species included and may thus represent plesiomorphic characters.

The specimens examined of *P.sciastra* display considerable morphological variation; the spore and subicular hyphal measurements of TAA187322 deviate markedly from those of SS359 and O F110317. Interestingly, *P.sciastra* is also more genetically variable than the other species studied.

For many species, we recorded a blue-green reaction of subhymenial hyphae, basidia, encrusting material and sometimes also of spores, to occur in KOH. We only observed the reaction occurring close to air bubbles or adjacent to the edges of cover glasses and we did not record it close to the centre of preparations free from air bubbles, unless they had been made slowly and hence had allowed air to come into contact with the entire samples before the application of a cover glass. The same structures often, but not always, also had an amyloid reaction in Melzer’s reagent. Both the blue green and the amyloid reaction could be used as species-separating characters (see further under “Taxonomy”). When present, the encrustation was most prevalent on the bases of basidia, but common also on the upper part of subhymenial hyphae. Occasionally, it was also appearing on subicular hyphae.

### Ecology and geographical distribution

**Figure 6.**
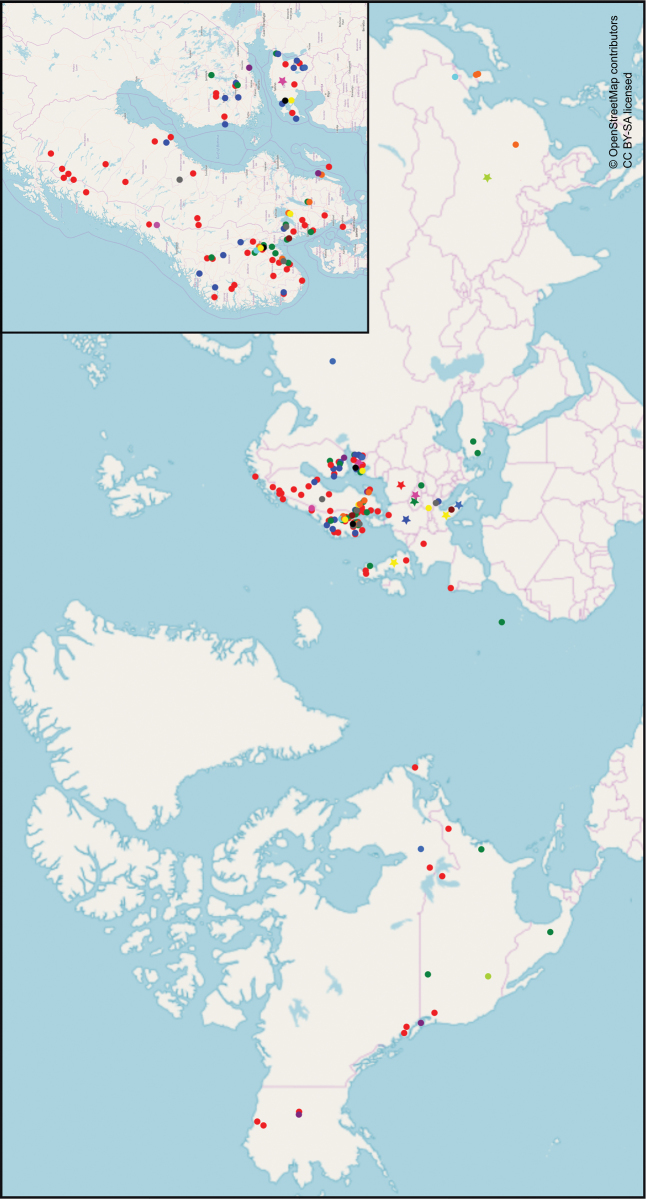
World distribution of new and previously described species in the *P.tristis* group, excluding doubtful taxa. Red – *P.umbrina*; pink – *P.tristoides*; dark green – *P.sciastra*; pale green – *P.atrofusca*; dark blue – *P.tristis*; pale blue – *P.media*; orange – *P.pinophila*; yellow – *P.rotundispora*; grey – *P.alobata*; black – *P.abundiloba*; brown – *P.umbrinascens*; turquoise – *P.alnophila*; purple – *P.pluriloba*.

We found the majority of the collections and sequences included in this study to belong to *P.umbrina* (Table 4). As shown by the origin of these (Fig. 6), *P.umbrina* is distributed across at least 12 countries in Europe and North America, where it has been found growing with 18 different hosts. It is present in Arctic/alpine vegetation above the treeline as well as in coniferous, deciduous and mixed forests and has been encountered on soils with pH ranging from low to high.

**Table 4.** Ecological data based on Scandinavian collection information and worldwide UNITE metadata.

Host	pH	Habitat	Basidiomata collections	Soil and root tip sequences
*Abiesalba*, *Alnusrubra*, *Betulanana*, B.pubescensssp.czerepanovii, B.pubescensssp.pubescens, *Dryasoctopetala*, *Fagussylvatica*, *Piceaabies*, *P.glauca*, *Piceamariana*, *Pinusbanksiana*, *P.pinaster*, *P.sylvestris*, *Pseudotsugamenziesii*, *Pyrolamedia*, *Quercuspetraea*, *Salixpolaris*, *Tsugacanadensis*	Low to high	Tundra Deciduous forest Coniferous forest Mixed forest	74	62
*Castaneasativa*, *Cedruslibani*, *Neottiaovata*, *Piceaabies*, *Quercus* sp.	Intermediate to high	Deciduous forest Coniferous forest Mixed forest	24	5
*Betulapendula*, *Fagussylvatica*	Intermediate to high	Deciduous forest Mixed forest	19	2
*Pinusdensiflora*, *P.massoniana*, *P.sylvestris*, *P.thunbergii*	High	Coniferous forest Mixed forest	9	3
*Castanea* sp., *P.tremula*	High	Deciduous forest Coniferous forest Mixed forest	5	4
*Betulapendula*, *Larixdecidua*, *Piceaglauca*	–	–	2	4
–	High	Coniferous forest Mixed forest	5	0
*Alnusincana*, *A.mandschurica*	Intermediate	Deciduous forest	2	2
* Pseudotsuga menziesii *	Intermediate	Mixed forest	2	2
* Rhododendron decorum *	–	–	1	3
*Cephalantheradamasonium*, *Populusalba*	Intermediate	Mixed forest	1	2
* Corylus avellana *	High	Deciduous forest	1	2
–	High	Mixed forest	2	0

The other species in the *P.tristis* group have been encountered markedly fewer times, with a smaller number of hosts, in less diverse habitats and mostly within a smaller geographical range. They have all been collected on soil with intermediate to high pH or both. Similarly to *P.umbrina*, however, many species seem to form ectomycorrhiza with a range of hosts; they have been collected on the root tips of both broadleaved and coniferous trees, as well as orchid species. Three species seem to have a limited host range: *P.pinophila* has only been found inhabiting the roots of *Pinus* L. species, while *P.alnophila* and *P.* sp. 2 have been found exclusively on the roots of *Alnus* Mill. Two species, meanwhile, now have a different confirmed geographical distribution than previously documented: the only verified sequences and basidiomata of *P.tristis* here studied originate in Europe, while *P.atrofusca* now have no confirmed findings there – the only validated findings are currently the Arizona holotype and three Chinese root tip sequences.

## Taxonomy

We provide descriptions of ten species new to science and of previously described accepted, dubious and excluded species in the *P.tristis* group. A worldwide key to all recognised and dubious species is also presented.

### Key to the species in the *P.tristis* group

*Pseudotomentella* species with brownish spores and subicular hyphae, lacking clamps and chlamydospores.

**Table:** 

1	Basidiome with hyphal cords containing skeletal hyphae, mean width of subicular hyphae 2.3 µm	*** P. atrofusca ***
–	Basidiome lacking hyphal cords and skeletal hyphae, mean width of subicular hyphae 3.4–6.5 µm	**2**
2	Basidiome when dried hard and brittle	*** H. rhacodium ***
–	Basidiome when dried soft cottony or soft, yet rather firm and compact and ± elastic	**3**
3	Basidiome when dried brown in all parts; blue or green colours are completely lacking in immature parts and in the subhymenium of mature parts. No blue green reaction in KOH (though basidia might be very pale green)	**4**
–	Basidiome when dried with blue or green colours in immature parts and in the subhymenium of mature parts. Subhymenial hyphae and basidia with blue green (often strong) reaction in KOH, in the presence of air	**5**
4	Mean length of echinuli 1.1–1.2 µm, mean length of sterigmata 9.6–10.5 µm, spores with three-six lobes or corners (rarely unlobed), basidia very pale greenish in KOH, sometimes with a slightly brown or blue hue, subiculum orange brown, immature hymenium and subhymenium initially pale brown	*** P. umbrina ***
–	Mean length of echinuli 1.6 µm, mean length of sterigmata 8.6 µm, spores with three-four lobes or corners (rarely five-six lobes), basidia pale brown to brown in KOH, sometimes with a greyish hue, subiculum pale yellowish-brown to pale orange brown, immature hymenium and subhymenium initially yellowish-white to pale brown	*** P. umbrinascens ***
5	Subicular hyphae narrow: mean width < 5 µm	**6**
–	Subicular hyphae wide: mean width > 5 µm	**10**
6	Spores short: mean length ≤ 7.8 µm	*** P. rotundispora ***
–	Spores long: mean length ≥ 8.7 µm	**7**
7	Basidiome when dried soft cottony in texture, hymenium bluish-grey (sometimes with a slightly brown hue) also when mature, close to *Alnus*	*** P. alnophila ***
–	Basidiome when dried soft, yet rather firm and compact and ± elastic, mature hymenium various shades of brown, with various hosts	**8**
8	Mean width of subicular hyphae 3.6–4.1 µm, mean width of subhymenial hyphae 3.9–4.0 µm, mean lateral spore width 6.3–6.6 µm, spores commonly roundedly star-shaped, often close to *Pinus*	*** P. pinophila ***
–	Mean width of subicular hyphae > 4.1 µm, mean width of subhymenial hyphae > 4.0 µm, mean lateral spore width ≥ 7.3 µm, spores generally angular-nodulose, with various hosts	**9**
9	Mean width of subicular hyphae 4.8–5.1 µm, noticeably wider than subhymenial hyphae, frontal face of spores with mean dimensions approximately 9.8 × 10.2 µm	*** P. pluriloba ***
–	Mean width of subicular hyphae 4.1–4.6 µm, with ± the same width as subhymenial hyphae, frontal face of spores with mean dimensions approximately 8.9–9.3 × 9.2–9.8 µm	*** P. media ***
10	Spores short: mean length < 8.5 µm	**11**
–	Spores long: mean length > 9.7 µm	**13**
11	Mean spore length 6.7–7.3 µm, spores star-shaped	*** P. sciastra ***
–	Mean spore length 8.2–8.6 µm, spores angular to nodulose	**12**
12	Mean length of echinuli 0.8 µm (maximal length 1.1 µm), mean sterigmal length approximately 8.6 µm	*** P. tristoides ***
–	Mean length of echinuli 1.4 µm (maximal length 1.7–1.9 µm), mean sterigmal length 9.4–10.2 µm	*** P. tristis ***
13	Mean lateral spore dimensions 10.9 × 8.5 µm, sterigmata very long – mean length 14.7 µm	*** P. longisterigmata ***
–	Mean lateral spore dimensions 9.6–9.9 × 7.1–7.7 µm, sterigmata normal – mean length 10.0–12.3 µm	**14**
14	Spores with three-five lobes or corners, mean width of subicular hyphae 4.8–5.1 µm, mean sterigmal length 11.5–12.3 µm, mean frontal spore width 10.2 µm	*** P. pluriloba ***
–	Spores either unlobed or with four-seven lobes or corners, mean width of subicular hyphae 5.5–6.1 µm, mean sterigmal length 10.0–11.5 µm, mean frontal spore width 9.5–9.8 µm	**15**
15	Spores unlobed, amyloid reaction observed in encrustation on basidia and subhymenial hyphae	*** P. alobata ***
–	Spores with four-seven lobes or corners, amyloid reaction not seen in encrustation on basidia and subhymenial hyphae	*** P. abundiloba ***

### Accepted taxa

#### Pseudotomentella abundiloba

Taxon classificationFungiThelephoralesThelephoraceae

Svantesson sp. nov.

MB828974

Fig. 7

##### Type.

NORWAY. Oslo (county): Oslo (municipality), Bygdøy, Hengsåsen, boreonemoral mixed forest on soil with high pH, 22 September 2010, S. Svantesson (holotype: O F110312!, GenBank Acc. No. ITS: MK290731).

##### UNITE SH.

SH032598.07FU

##### Etymology.

The name refers to the spores, which are abundantly lobed.

##### Description.

**Basidiomata** annual, resupinate, membranaceous, effused to several tens of centimetres in diameter. Mature parts continuous, with a rather firm, fibrous and compact, yet quite soft and elastic texture. Hymenium smooth, but sometimes strongly undulating; brown with a pinkish hue. Immature parts discontinuous, byssoid, with a cottony texture. Subhymenium and hymenium of immature parts blue grey to brown grey. Subiculum well developed, loose, fibrous, orange brown; often forms the outer edge of basidiomata, extending noticeably beyond the hymenium. All characters recorded in dried state.

**Figure 7.**
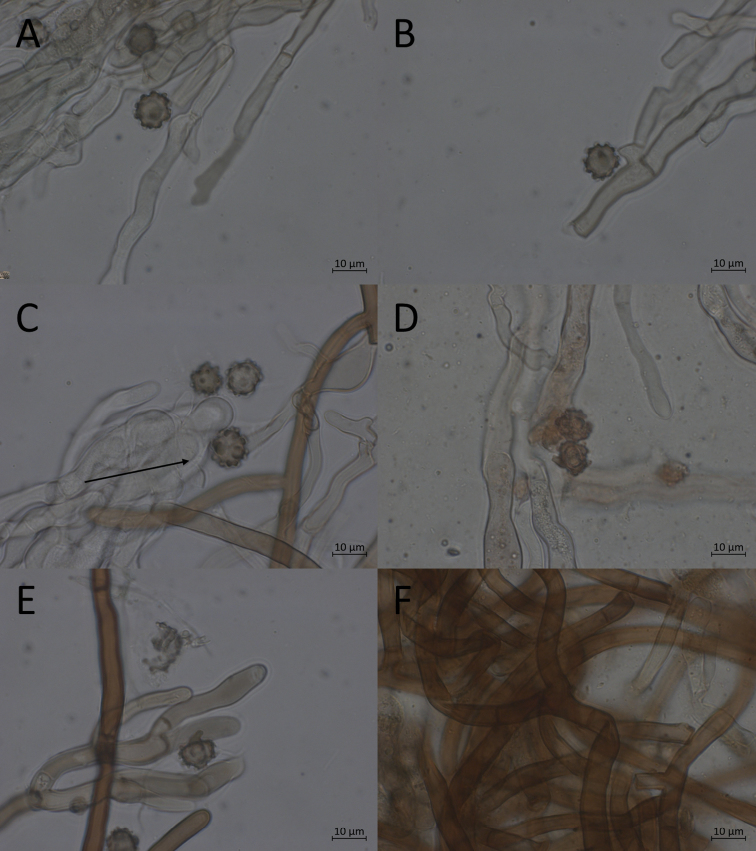
Micromorphological features of *P.abundiloba* in KOH. **A, B** basidiospores in frontal face (TU 110852) **C** in tilted frontal face (TU 110852) **D, E** in lateral face (TU 110852) **F** subicular hyphae (holotype).

**Hyphal cords** lacking, but loose bundles of subicular hyphae sometimes present.

**Hyphal system** monomitic; clamp connections and reaction in Melzer’s reagent absent from all hyphae.

**Subicular hyphae** noticeably long and straight, thick-walled; forming a loose tissue. Individual hyphae (4.3–) 4.8–6.9 (–7.2) μm wide, with a mean width of 5.5–6.1 μm; orange brown to dark brown in both KOH and water.

**Subhymenial hyphae** often somewhat sinuous, thin to thick-walled; forming a rather dense tissue. Individual hyphae (4.0–) 4.1–6.8 (–7.2) μm wide, with a mean width of 5.5–5.7 μm; in the upper parts, hyaline to orange brown or orange green in KOH, with a blue green reaction in the presence of air; in the lower parts, pale orange brown to orange brown in KOH, unchanged in air; in water with strongly granular contents, orange green.

**Encrustation** granular, inamyloid; hyaline to orange brown or orange green in KOH, blue green in the presence of air; orange green in water; common to rare, usually scattered in occurrence on the upper parts of subhymenial hyphae and on the lower parts of basidia.

**Basidia** with four slightly curved sterigmata, occasionally two-sterigmate; clavate to narrowly clavate, sometimes clavopedunculate, thin-walled, with one-three slight constrictions. Dimensions: (63–) 64–92 (–93) × (8.7–) 10.0–14.4 (14.9) μm; mean dimensions: 70–81 × 11.0–12.0 μm. Sterigmata (8.4–) 9.0–12.9 (–13) μm long, with a mean length of 10.0–11.5 μm. Colours and reactions the same as for the upper parts of the subhymenial hyphae, but in addition often with granular contents in KOH.

**Cystidial organs** lacking.

**Basidiospores** in frontal face generally with a subcircular basic shape and a star-shaped, angular, nodulose or sometimes cross-shaped outline, covered in bi- or trifurcate, sometimes singularly attached, echinuli. Nearly all spores with four-seven, low but distinct, rounded to square lobes or corners; unlobed, broadly ovoid spores and rounded, heart-shaped spores infrequently occurring, as well as abnormally large spores originating from two-sterigmate basidia. Frontal dimensions: (8.8–) 9.2–10.5 × (8.0–) 8.6–10.7 (–10.8) μm; mean dimensions: 9.8 × 9.5–9.6 μm; Q-value: 0.9–1.2; mean Q-value: 1.0. Echinuli (0.9–) 1.1–1.8 (–1.9) μm long, with a mean length of 1.2–1.5 μm. Lateral face ellipsoid to semicircular, usually with evenly rounded edges, sometimes with one-three lobes. Lateral dimensions: (8.9–) 9.3–10.1 (–10.5) × (6.7–) 7.0–8.1 (–8.2) μm; mean dimensions: 9.7–9.8 × 7.3–7.7 μm; Q-value: 1.2–1.4 (–1.5); mean Q-value: 1.3. Colour in KOH pale orange green to orange brown, in the presence of air sometimes with a blue green reaction; in water pale orange green; inamyloid.

**Chlamydospores** lacking.

##### Habitat.

The type collection was obtained in an old, mixed forest on soil with high pH. No additional sequences are available in UNITE.

##### Distribution.

Basidiomata encountered in: Estonia and Norway.

##### Remarks.

Within the *P.tristis* group, the basidiomata of *P.abundiloba* are recognised by their lack of hyphal cords and skeletal hyphae and their soft, yet rather firm and compact and ± elastic texture after drying, bluish to greenish colour of immature parts, wide subicular hyphae, long, abundantly lobed spores and inamyloid encrustation on subhymenial hyphae and basidia. *Pseudotomentellaabundiloba*, *P.pluriloba* and *P.media* can appear similar, but none of them has abundantly lobed spores. *Pseudotomentellamedia* further differs by having smaller spores and narrower subicular hyphae, while *P.pluriloba* has narrower subicular hyphae, longer sterigmata and frontally wider spores and *P.alobata* has amyloid encrustation on its subhymenial hyphae and basidia.

##### Additional specimens studied.

ESTONIA. Lääne: Ridala, between Uneste and Võnnu, Ehmja-Turvalepa Special Conservation Area, nutrient-rich, boreonemoral forest, 25 September 2012, L. Tedersoo (TU 110852*).

#### Pseudotomentella alnophila

Taxon classificationFungiThelephoralesThelephoraceae

Svantesson sp. nov.

MB828998

Fig. 8

##### Type.

NORWAY. Buskerud: Ringerike, Juveren N, boreonemoral *Alnusincana* forest on soil with intermediate pH, 25 September 2010, S. Svantesson and N. Svensson (holotype: O F110313!, GenBank Acc. No. ITS: MK290715).

##### UNITE SH.

SH218588.07FU

##### Etymology.

The name refers to the ectomycorrhizal association of the species, which always seems to be with *Alnus*.

##### Description.

**Basidiomata** annual, resupinate, membranaceous, effused. Mature parts continuous, with a soft cottony texture. Hymenium smooth; blue grey, sometimes with a slightly brown hue. Immature parts discontinuous, byssoid, with a soft cottony texture. Subhymenium and hymenium of immature parts pale blue grey to blue grey. Subiculum thin to well developed, loose, fibrous, orange brown; often forms the outer edge of basidiomata, extending noticeably beyond the hymenium. All characters recorded in dried state.

**Figure 8.**
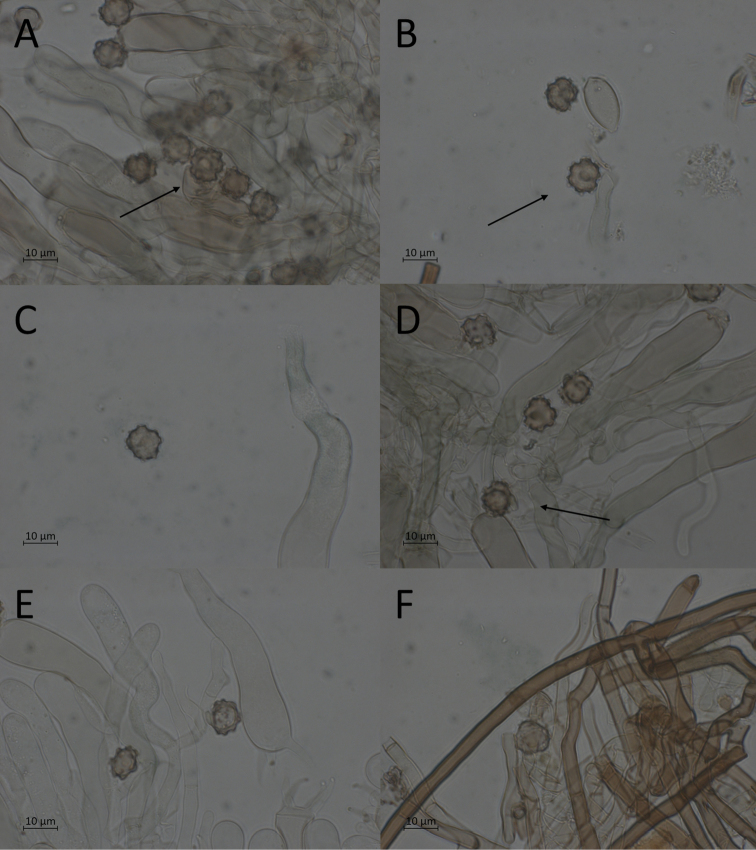
Micromorphological features of *P.alnophila* in KOH. Holotype: **A, B, C** basidiospores in frontal face **D, E** in lateral face **E** subicular hyphae.

**Hyphal cords** lacking, but loose bundles of subicular hyphae sometimes present.

**Hyphal system** monomitic, clamp connections and reaction in Melzer’s reagent absent from all hyphae.

**Subicular hyphae** noticeably long and straight, thick-walled; forming a loose tissue. Individual hyphae 4.0–5.0 (–5.1) μm wide, with a mean width of 4.5 μm; orange brown to dark brown in KOH and orange to orange brown in water.

**Subhymenial hyphae** often somewhat sinuous, thin to thick-walled; forming a rather dense tissue. Individual hyphae (3.2–) 3.4–5 (–5.6) μm wide, with a mean width of 4.1 μm; hyaline to pale orange brown in KOH, blue green in the presence of air; pale green in water, with strongly granular contents.

**Encrustation** not seen.

**Basidia** with four slightly curved sterigmata, occasionally two-sterigmate; clavate to narrowly clavate, sometimes clavopedunculate, thin-walled, with one-three slight constrictions. Dimensions: (66–) 67–93 (–100) × (11.2–) 11.3–14.2 (–15.0) μm; mean dimensions: 83 × 12.8 μm. Sterigmata (9.5–) 11–14.5 (–14.8) μm long, with a mean length of 8.6 μm. Colours and reactions the same as for the subhymenial hyphae, but in addition often with granular contents in KOH.

**Cystidial organs** lacking.

**Basidiospores** in frontal face generally with a subcircular basic shape and an angular to nodulose or sometimes cross-shaped outline, covered in bi- or trifurcate, sometimes singularly attached, echinuli. Nearly all spores with three-five distinct corners or rounded to square lobes; broadly ovoid spores and rounded, heart-shaped spores infrequently occurring, as well as abnormally large spores originating from two-sterigmate basidia. Frontal dimensions: (8.8–) 9.0–10.1 (–10.4) × 9.2–10.2 (–10.6) μm; mean dimensions: 9.5 × 9.8 μm; Q-value: 0.9–1.0; mean Q-value: 1.0. Echinuli (0.8–) 0.9–1.7 μm long, with a mean length of 1.2 μm. Lateral face ellipsoid to ovoid, usually with evenly rounded edges, sometimes with one-three lobes. Lateral dimensions: 9.0–10.6 × (6.6–) 6.9–8.1 (–8.2) μm; mean dimensions: 9.6 × 7.7 μm; Q-value: 1.2–1.3 (–1.4); mean Q-value: 1.3. Colour in KOH pale orange brown to pale orange green, in the presence of air occasionally with a blue green reaction; in water pale green to pale orange green; inamyloid.

**Chlamydospores** lacking.

##### Habitat.

The only specimens recorded to date of *P.alnophila* is the type collection and one other collection from the same locality, which is a mature and, at the collection site pure, stand of *Alnusincana* on clay soil with intermediate pH. In addition, UNITE sequence metadata show that the species forms ectomycorrhiza with at least *Alnusmandschurica* (Kõljalg et al. 2005, Nilsson et al. 2019).

##### Distribution.

Basidiomata encountered in: Norway. Soil or root tip samples confirm presence also in: China.

##### Remarks.

Within the *P.tristis* group, the basidiomata of *P.alnophila* can be recognised by their lack of hyphal cords and skeletal hyphae and their soft cottony texture after drying, bluish to greenish colour of immature parts, narrow hyphae, long spores, bluish-grey mature hymenium (sometimes with a slightly brown hue) and their association with *Alnus*. *Pseudotomentellapluriloba*, *P.media* and *P.pinophila* are similar, but they all have basidiomata that are compact and rather firm after drying and whose mature parts are some shade of brown, without any bluish hue. *Pseudotomentellapluriloba* also has slightly longer spores and echinuli and wider subicular hyphae, while *P.media* and *P.pinophila* have generally slightly smaller microcharacters. *Pseudotomentellapinophila* also has a different spore shape. In addition, neither of these species has been recorded as being associated with *Alnus*.

##### Additional specimens studied.

NORWAY. Buskerud: Ringerike, Juveren N, boreonemoral, *Alnusincana* forest on soil with intermediate pH, 25 September 2010, S. Svantesson and N. Svensson (O F110314).

#### Pseudotomentella alobata

Taxon classificationFungiThelephoralesThelephoraceae

Svantesson sp. nov.

MB828999

Fig. 9

##### Type.

SWEDEN. Dalsland, Mellerud, Skållerud, Norgekullen SW, coniferous forest on soil with high pH, 20 September 2017, S. Svantesson 425 (holotype: GB!, GenBank Acc. No. ITS: MK290696).

##### UNITE SH.

SH030577.07FU

##### Etymology.

The name refers to the spores, which commonly lack lobation.

##### Description.

**Basidiomata** annual, resupinate, membranaceous, effused – often to several tens of centimetres in diameter. Mature parts continuous, with a cottony texture when fresh and a rather firm, fibrous and compact, yet quite soft and elastic texture when dried. Hymenium smooth, but sometimes strongly undulating; brown, purplish-brown or blue-greyish-brown when fresh, brown with a pinkish hue when dried. Immature parts discontinuous, byssoid, with a cottony texture both when fresh and when dried. Subhymenium and hymenium of immature parts blue to blue grey when fresh and blue grey to brown grey when dried. Subiculum well developed, loose, fibrous, orange brown; often forms the outer edge of basidiomata, extending noticeably beyond the hymenium.

**Figure 9.**
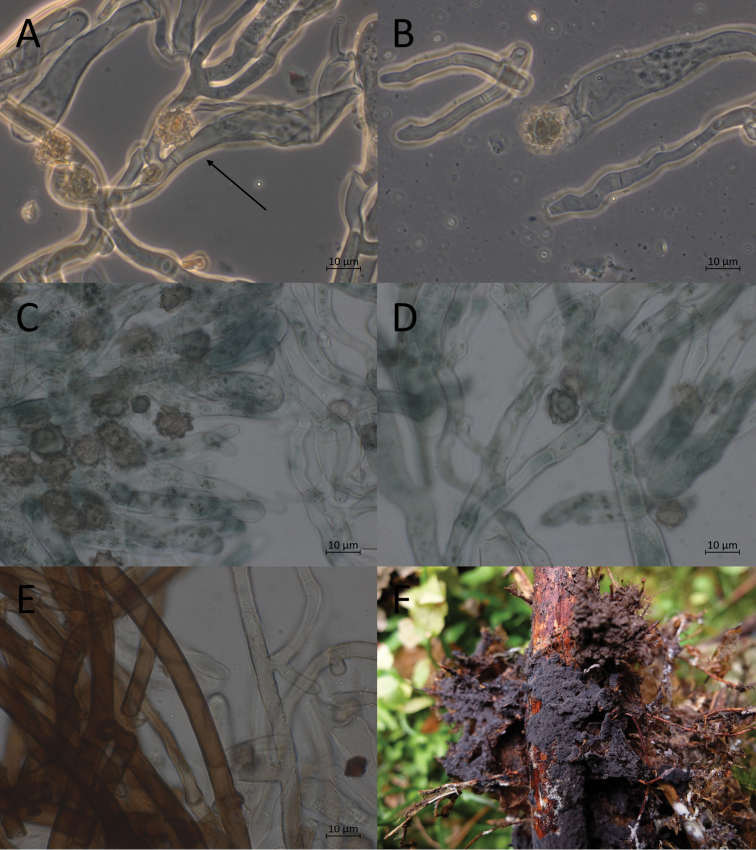
Morphological features of *P.alobata*, mounted in KOH and macroscopically. **A, B** basidiospores in frontal face (O F110315) **C, D** in lateral face (O F110316) **E** subicular hyphae (TU 115626) **F** mature basidiome (holotype).

**Hyphal cords** lacking, but loose bundles of subicular hyphae sometimes present.

**Hyphal system** monomitic, clamp connections absent from all hyphae.

**Subicular hyphae** noticeably long and straight, thick-walled; forming a loose tissue. Individual hyphae (4.3–) 4.6–7.4 (–7.6) μm wide, with a mean width of 5.6–5.9 μm; orange in both KOH and water.

**Subhymenial hyphae** often somewhat sinuous, thin to thick-walled; forming a rather dense tissue. Individual hyphae (3.1–) 3.4–6.9 μm wide, with a mean width of 4.0–4.5 μm; hyaline to pale green in KOH, blue green in the presence of air; yellow to pale orange yellow in water, with strongly granular contents.

**Encrustation** granular, amyloid; purple in KOH, dark blue green in the presence of air; dark brown in water; usually common and scattered in occurrence on the upper parts of subhymenial hyphae and on the lower parts of basidia.

**Basidia** with four slightly curved sterigmata, occasionally two-sterigmate; clavate to narrowly clavate, sometimes clavopedunculate, thin-walled, with one-three slight constrictions. Dimensions: (63–) 64–91 (–98) × (10.2–) 10.5–14.2 (–14.3) μm; mean dimensions: 74–77 × 11.3–12.1 μm. Sterigmata 8.5–12.1 (–12.4) μm long, with a mean length of 10.0–10.3 μm. Colours and reactions the same as for the subhymenial hyphae, but in addition often with granular contents in KOH.

**Cystidial organs** lacking.

**Basidiospores** in frontal face generally with a subcircular basic shape and an unlobed or occasionally weakly pronounced, rounded, heart-shaped outline, covered in bi- or trifurcate, sometimes singularly attached, echinuli. Subcircular, three-five-lobed spores infrequently occurring, as well as abnormally large spores originating from two-sterigmate basidia. Frontal dimensions: (9.0–) 9.1–10.7 × (8.4–) 8.9–10.5 (–10.7) μm; mean dimensions: 9.7–10.1 × 9.5–9.8 μm; Q-value: (0.9–) 1.0–1.1; mean Q-value: 1.0. Echinuli 1.2–1.8 (–1.9) μm long, with a mean length of 1.4–1.7 μm. Lateral face ellipsoid, usually with evenly rounded edges, sometimes with one-three lobes. Lateral dimensions: (8.9–) 9.1–10.3 × (6.5–) 6.7–8.2 μm; mean dimensions: 9.7–9.9 × 7.1–7.4 μm; Q-value: (1.2–) 1.3–1.5; mean Q-value: 1.3–1.4. Colour in KOH pale brownish-yellow, in the presence of air often with a blue green reaction; in water pale greenish-yellow to pale orange yellow; occasionally amyloid.

**Chlamydospores** lacking.

##### Habitat.

Data on habitat are scarce to date, but recent Scandinavian collections have been made in old growth coniferous or mixed forests on soil with high pH.

##### Distribution.

Basidiomata encountered in: Norway, Slovenia and Sweden. No sequences originating from soil or root tip samples in UNITE.

##### Remarks.

Within the *P.tristis* group, the basidiomata of *P.alobata* are recognised by their lack of hyphal cords and skeletal hyphae and their soft, yet rather firm and compact and ± elastic texture after drying, bluish to greenish colour of immature parts, wide subicular hyphae, long, unlobed spores and amyloid encrustation on subhymenial hyphae and basidia. *Pseudotomentellaabundiloba*, *P.pluriloba* and *P.media* can appear similar, but none of them has spores which generally are unlobed. *P.media* further differs by having smaller spores and narrower subicular hyphae, while *P.pluriloba* has narrower subicular hyphae, longer sterigmata and frontally wider spores than *P.alobata*. *Pseudotomentellaabundiloba* sometimes has encrusted subhymenial hyphae and basidia, but without amyloid reaction.

##### Additional specimens studied.

NORWAY. Telemark: Bamble, Rognsflaugane, boreonemoral, mixed forest on soil with high pH, 2 September 2010, K.-H. Larsson and S. Svantesson (O F110316*); Telemark: Tokke, Dalen, Huvestad, boreonemoral, mixed forest on soil with high pH, 28 September 2010, S. Svantesson and N. Svensson (O F110315*);

SLOVENIA. Radovljica: Triglav National Park, Pokljuka plateau, transition zone between secondary spruce forest (in parts with remnants of primary *Fagussylvatica/Acer pseudoplatanus* forest) and natural *Larixdecidua* stand with individual trees of *Pinusmugo*, *Sorbusaucuparia* and *Salix* sp., 1530 m a.s.l., 20 September 2012, U. Kõljalg (TU 115626*);

SWEDEN. Ångermanland: Edsele, Djupdalsmyran, Stordjupdalen, on *Piceaabies*, 29 August 2002, K.-H. Larsson 11873* (GB 0087566).

#### Pseudotomentella atrofusca

Taxon classificationFungiThelephoralesThelephoraceae

M.J.Larsen, Bull. Torrey Bot. Club. 98: 39 (1971)

Fig. 10

##### Type.

UNITED STATES. Arizona: Fort Valley, Coconino, on *Pinusponderosa* Laws., 21 September 1967, R. L. Gilbertson 7553 (holotype: ARIZ!, GenBank Acc. No. ITS: MK290732; isotype: SSMF 685–4578).

##### UNITE SH.

SH005338.07FU

##### Description.

**Basidiome** annual, resupinate, membranaceous, effused. Mature parts continuous, with a cottony texture. Hymenium smooth, brown. Immature parts discontinuous, byssoid, with a cottony texture. Subhymenium and hymenium of immature parts initially whitish-grey to whitish-grey brown, when more mature blue grey to brown grey. Subiculum thin, loose, fibrous, pale brown; often forms the outer edge of the basidiome, extending noticeably beyond the hymenium. All characters recorded in dried state.

**Figure 10.**
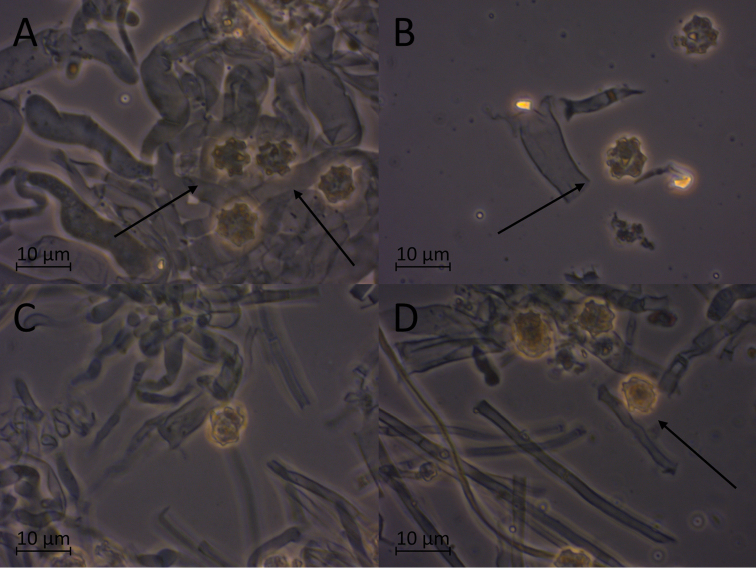
Micromorphological features of *P.atrofusca* in KOH. Holotype: **A, B** basidiospores in frontal face **C, D** in lateral face.

**Hyphal cords** connecting to the edge of the basidiome and thinning out underneath; whitish to pale brown. Individual cords dimitic; formed by a sheathing layer of skeletal hyphae and two layers of generative hyphae; the outer generative hyphae thinner and the inner ones wider, the latter often swollen interseptally. Skeletal hyphae 1.1–1.4 (–1.5) μm wide, with a mean width of 1.3 μm. Outer generative hyphae (2.2) 2.3–2.9 μm wide, with a mean width of 2.6 μm. Inner generative hyphae (3.8) 3.9–5.3 (5.5) μm wide, with a mean width of 4.5 μm. All hyphae pale yellowish-brown in both KOH and water.

**Hyphal system** dimitic, clamp connections and reaction in Melzer’s reagent absent from all hyphae.

**Subicular hyphae** of two kinds: (1) generative hyphae noticeably long and straight, thick-walled; forming a loose tissue, in which (2) skeletal hyphae occur sparsely (most common in areas to where hyphal cords attach). Generative hyphae (1.7–) 1.8–2.8 μm wide, with a mean width of 2.3 μm; pale yellowish-brown to yellowish-brown in both KOH and water. Skeletal hyphae with the same width and colour as in the hyphal cords.

**Subhymenial hyphae** often somewhat sinuous, thin to thick-walled; forming a rather dense tissue. Individual hyphae (1.8–) 2.1–3.0 μm wide, with a mean width of 2.5 μm; hyaline to pale green in KOH, blue green in the presence of air; hyaline to pale blue green in water, with strongly granular contents.

**Basidia** with four slightly curved sterigmata, occasionally two-sterigmate; clavate to narrowly clavate, sometimes clavopedunculate, thin-walled, with one-three slight constrictions. Dimensions: (33–) 34–56 (–59) × (5.8–) 6.2–7.8 (7.9) μm; mean dimensions: 44 × 7.2 μm. Sterigmata 4.4–5.6 (–6.8) μm long, with a mean length of 5.1 μm. Colours and reactions the same as for the subhymenial hyphae, but in addition often with granular contents in KOH.

**Cystidial organs** lacking.

**Basidiospores** in frontal face generally with a subcircular basic shape and an angular to nodulose or sometimes star or cross-shaped outline, covered in bi- or trifurcate, sometimes singularly attached, echinuli. Nearly all spores with five distinct, square lobes, but depending on the precise angle sometimes perceived as three; four-lobed spores occasionally occurring; abnormally large spores originating from two-sterigmate basidia infrequently seen. Frontal dimensions: (6.1–) 6.2–7.0 (–7.1) × (5.8–) 6.3–7.2 (–7.3) μm; mean dimensions: 6.6 × 6.8 μm; Q-value: 0.9–1.0; mean Q-value: 1.0. Echinuli 0.6–0.9 (–1.1) μm long, with a mean length of 0.8 μm. Lateral face ellipsoid to ovoid, usually with evenly rounded edges, sometimes with one-three lobes. Lateral dimensions: 6.3–6.9 (–7.3) × (4.0–) 4.1–4.8 (–5.0) μm; mean dimensions: 6.5 × 4.4 μm; Q-value: (1.3–) 1.4–1.6; mean Q-value: 1.5. Colour in both KOH and water pale brownish-yellow to pale blue green; inamyloid.

**Chlamydospores** lacking.

##### Habitat.

The only specimen recorded to date of *P.atrofusca* is the type collection, which was collected on wood of *Pinusponderosa*. Available UNITE sequences originate from root tips of *Rhododendrondecorum* (Kõljalg et al. 2005, Nilsson et al. 2019).

##### Distribution.

Basidiome encountered in: United States. Soil or root tip samples confirm presence also in: China.

##### Remarks.

Within the *P.tristis* group, the basidiome of *P.atrofusca* can be recognised by its hyphal cords and skeletal hyphae. These features make it unique within the group and the risk for confusion with any other described species should be small. Outside the *P.tristis* group, *P.rhizopunctata* is somewhat similar, but differs from *P.atrofusca* by the presence of chlamydospores on its hyphal cords.

#### Pseudotomentella media

Taxon classificationFungiThelephoralesThelephoraceae

Svantesson & Kõljalg sp. nov.

MB829000

Fig. 11

##### Type.

ESTONIA. Valga: Otepää, Trommi, 12 September 2012, U. Kõljalg (holotype: TU 115609!, GenBank Acc. No. ITS: MK290714).

##### UNITE SH.

SH005336.07FU

##### Etymology.

The name refers to the middling morphological characters of the species, relative to other species in the *P.tristis* group.

##### Description.

**Basidiomata** annual, resupinate, membranaceous, effused to approximately ten centimetres in diameter. Mature parts continuous, with a rather firm, fibrous and compact, yet quite soft and elastic texture when dried. Hymenium smooth, but sometimes strongly undulating; brown with a reddish hue, both when fresh and when dried. Immature parts discontinuous, byssoid, with a cottony texture when dried. Subhymenium and hymenium of immature parts blue to greenish-blue when fresh and pale blue grey or blue grey to grey brown when dried. Subiculum well developed, loose, fibrous, pale brown to pale orange brown; forms the outer edge of basidiomata, extending noticeably beyond the hymenium.

**Figure 11.**
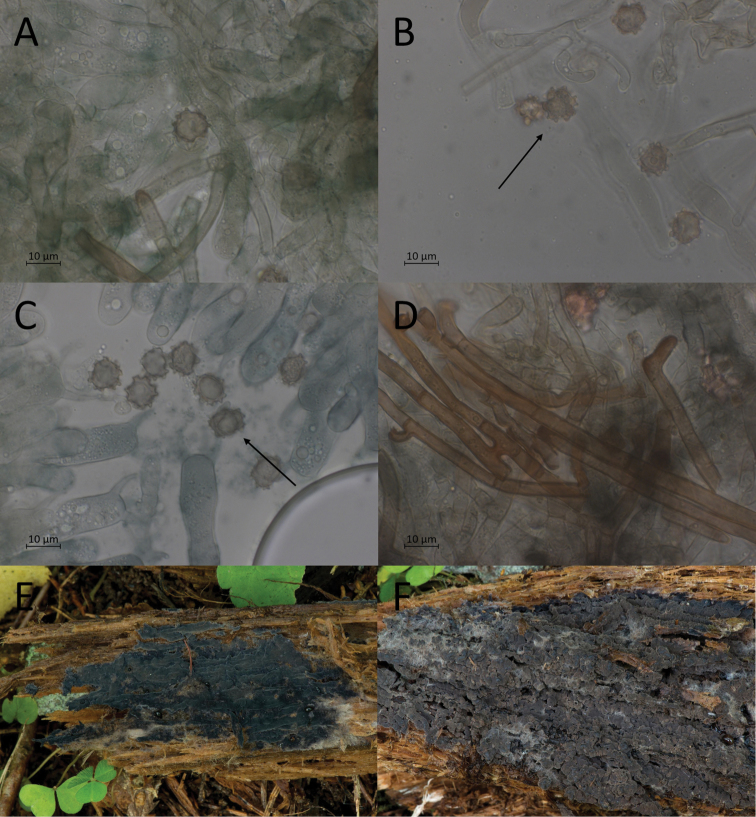
Morphological features of *P.media*, mounted in KOH and macroscopically. **A, B** basidiospores in frontal face (TU 115608) **C** in lateral face (TU 115608) **D** subicular hyphae (TU 115608) **E** younger basidiome (TU 115608) **F** mature basidiome (holotype).

**Hyphal cords** lacking, but loose bundles of subicular hyphae sometimes present.

**Hyphal system** monomitic, clamp connections absent from all hyphae.

**Subicular hyphae** noticeably long and straight, thick-walled; forming a loose tissue. Individual hyphae (3.6–) 3.7–5.0 (–5.4) μm wide, with a mean width of 4.1–4.6 μm; orange brown to brown in both KOH and water.

**Subhymenial hyphae** often somewhat sinuous, thin to thick-walled; forming a rather dense tissue. Individual hyphae (3.2–) 3.4–5.6 (–5.7) μm wide, with a mean width of 4.3–4.4 μm; pale brown, pale orange brown, pale greenish-brown or hyaline in KOH, blue green in the presence of air; orange green to brown in water, with strongly granular contents. Some subhymenial hyphae with a pink colour in water and an amyloid reaction.

**Encrustation** granular, probably amyloid (hard to observe due to the colour); blackish in KOH, dark blue green in the presence of air; blackish in water; scattered in occurrence on the upper parts of subhymenial hyphae and on the lower parts of basidia.

**Basidia** with four slightly curved sterigmata, occasionally two-sterigmate; clavate or sometimes narrowly clavate or clavopedunculate, thin-walled, with one-three slight constrictions. Dimensions: (57–) 58–84 (–87) × (8.8–) 9.6–11.8 (–12.5) μm; mean dimensions: 74–77 × 10.2–10.7 μm. Sterigmata (9.4–) 9.5–11.4 (–11.7) μm long, with a mean length of 10.0–10.8 μm. Colours and reactions the same as for the subhymenial hyphae, but in addition often with granular contents in KOH.

**Cystidial organs** lacking.

**Basidiospores** in frontal face generally with a subcircular basic shape and an angular to nodulose or sometimes cross-shaped outline, covered in bi- or trifurcate, sometimes singularly attached, echinuli. A majority of the spores with three-six indistinct corners to distinct, square lobes; unlobed subellipsoid spores infrequently occurring, as well as abnormally large spores originating from two-sterigmate basidia. Frontal dimensions: (7.8–) 8.0–9.5 × (8.3–) 8.5–9.9 (–10.1) μm; mean dimensions: 8.9–9.3 × 9.2–9.8 μm; Q-value: 0.9–1.0; mean Q-value: 1.0. Echinuli (0.8–) 0.9–1.6 μm long, with a mean length of 1.1–1.2 μm. Lateral face ellipsoid, usually with evenly rounded edges, sometimes with one-three lobes. Lateral dimensions: (8.8–) 8.9–9.6 × (6.6–) 7.0–7.9 μm; mean dimensions: 9.1–9.4 × 7.3–7.6 μm; Q-value: 1.2–1.3; mean Q-value: 1.2–1.3. Colour in KOH pale brown to brown or pale orange brown to orange brown, in the presence of air sometimes blue green; in water orange brown to brown; inamyloid.

**Chlamydospores** lacking.

##### Habitat.

*P.media* has been found to form ectomycorrhiza with at least *Betulapendula*, *Larixdecidua* and *Piceaglauca* (Kõljalg et al. 2005, Nilsson et al. 2019).

##### Distribution.

Basidiomata encountered in: Estonia. Soil or root tip samples confirm presence also in: Canada, Italy and Russia.

##### Remarks.

Within the *P.tristis* group, the basidiomata of *P.media* can be recognised by their lack of hyphal cords and skeletal hyphae and their soft, yet rather firm and compact and ± elastic texture after drying, bluish to greenish colour of immature parts, narrow subicular hyphae, brown mature hymenium, long, laterally wide, angular-nodulose spores and subhymenial hyphae that are of ± the same width as the subicular hyphae. *Pseudotomentellapinophila* and *P.pluriloba* can appear similar, but the spores of *P.pinophila* are laterally narrower and generally star-shaped, while *P.pluriloba* has wider subicular hyphae, larger spores and subhymenial hyphae that are noticeably narrower than the subicular hyphae.

##### Additional specimens studied.

ESTONIA. Valga: Otepää, Trommi, 12 September 2012, U. Kõljalg (TU 115608*).

#### Pseudotomentella pinophila

Taxon classificationFungiThelephoralesThelephoraceae

Svantesson sp. nov.

MB829001

Fig. 12

##### Type.

SWEDEN. Småland: Jönköping, Svarttorp, Ramlaklint, boreonemoral, mixed, old-growth forest, on soil with intermediate pH, 12 September 2016, S. Svantesson 358 (holotype: GB!, GenBank Acc. No. ITS: MK290708).

##### UNITE SH.

SH005337.07FU

##### Etymology.

The name refers to the ectomycorrhizal association of the species, which often seems to be with *Pinus*.

##### Description.

**Basidiomata** annual, resupinate, membranaceous, effused – often to several tens of centimetres in diameter. Mature parts continuous, with a cottony texture when fresh and a rather firm, fibrous and compact, yet quite soft and elastic texture when dried. Hymenium smooth, but sometimes strongly undulating; pale brown to pale greenish-brown when fresh, pale reddish-brown when dried. Immature parts discontinuous, byssoid with a cottony texture, both when fresh and when dried. Subhymenium and hymenium of immature parts blue to grey when fresh, pale blue or blue grey to dark blue grey or brown grey, sometimes with a green hue, when dried. Subiculum well-developed, loose, fibrous, pale orange brown; often forms the outer edge of basidiomata, extending noticeably beyond the hymenium.

**Figure 12.**
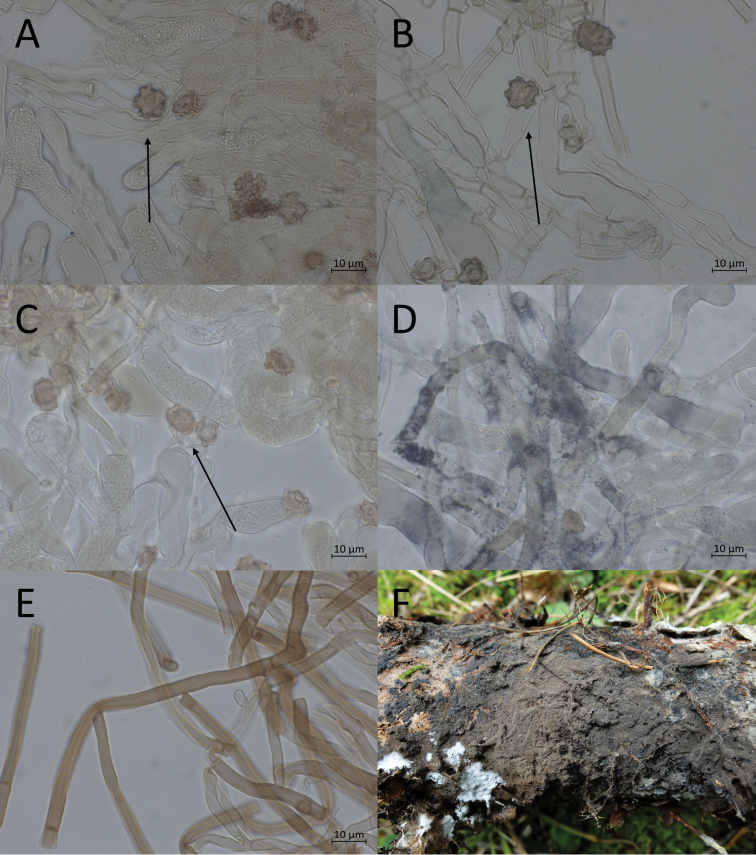
Morphological features of *P.pinophila*, mounted in KOH and macroscopically. **A, B** basidiospores in frontal face (holotype) **C** in lateral face (O F110330) **D** encrusted subhymenial hyphae (O F110305) **E** subicular hyphae (O F110330) **F** mature basidiome (SS418).

**Hyphal cords** lacking, but loose bundles of subicular hyphae sometimes present.

**Hyphal system** monomitic, clamp connections absent from all hyphae.

**Subicular hyphae** noticeably long and straight, thick-walled; forming a loose tissue. Individual hyphae 3.0–4.9 μm wide, with a mean width of 3.6–4.1 μm; pale orange brown to pale pinkish-brown in both KOH and water.

**Subhymenial hyphae** often somewhat sinuous, thin to thick-walled; forming a rather dense tissue. Individual hyphae (3.1–) 3.2–4.7 (–5.2) μm wide, with a mean width of 3.9–4.0 μm; hyaline to pale green or occasionally pale brown in KOH, blue green in the presence of air; pale green to pale orange in water, with strongly granular contents; sometimes with an amyloid reaction in the cell walls.

**Encrustation** granular, amyloid; bluish-black in both KOH and water; common to rare, usually scattered in occurrence on the upper parts of subhymenial hyphae and on the lower parts of basidia.

**Basidia** with four slightly curved sterigmata, occasionally two-sterigmate; clavate to narrowly clavate, sometimes clavopedunculate, thin-walled, with one-three slight constrictions. Dimensions: (53–) 58–74 (–83) × (9.0–) 9.1–11.7 (–12.1) μm; mean dimensions: 65–67 × 9.9–10.3 μm. Sterigmata (8.2–) 8.8–10.5 (–11.9) μm long, with a mean length of 9.6–10.0 μm. Colours and reactions the same as for the subhymenial hyphae, but in addition often with granular contents in KOH.

**Cystidial organs** lacking.

**Basidiospores** in frontal face commonly with a subcircular basic shape and a roundedly star-shaped, sometimes roundedly cross-shaped or angular to nodulose outline, covered in bi- or trifurcate, occasionally singularly attached, echinuli. Lobes distinct, rounded to square; predominantly five, but commonly also three or four; six-lobed or subellipsoid, unlobed spores and spores with corners instead of lobes infrequently occurring, as well as abnormally large spores originating from two-sterigmate basidia. Frontal dimensions: (7.7–) 7.9–10.2 (–10.3) × (7.7–) 8.3–10.1 (–10.2) μm; mean dimensions: 8.6–9.1 × 8.8–9.4 μm; Q-value: 0.9–1.1; mean Q-value: 1.0. Echinuli (0.6–) 0.8–1.4 (–1.5) μm long, with a mean length of 0.9–1.1 μm. Lateral face ellipsoid to ovoid, usually with evenly rounded edges, sometimes with angular edges or one-three lobes. Lateral dimensions: (8.2–) 8.3–9.7 (–9.8) × (5.7–) 5.8–6.8 (–7.0) μm; mean dimensions: 8.7–9.0 × 6.3–6.6 μm; Q-value: 1.3–1.6; mean Q-value: 1.3–1.4. Colour in KOH pale green to orange brown, in the presence of air sometimes with a blue green reaction; in water pale green to orange brown or brown; occasionally amyloid.

**Chlamydospores** lacking.

##### Habitat.

Data on habitat are scarce to date, but recent Scandinavian collections have been made in old coniferous or mixed forests on soil with high pH. *Pseudotomentellapinophila* has been found to form ectomycorrhiza with at least *Pinusdensiflora*, *Pinusmassoniana*, *Pinussylvestris* and *Pinusthunbergii* (Kõljalg et al. 2005, Nilsson et al. 2019). It should be noted however that, although the only hitherto documented hosts of *P.pinophila* belong to the genus *Pinus* and *P.sylvestris* has indeed been present at nearly all Nordic localities of collection, a few of these collections were made at localities where *Pinus* was not recorded as a possible host.

##### Distribution.

Basidiomata encountered in: Norway and Sweden. Soil or root tip samples confirm presence also in: China and Republic of Korea.

##### Remarks.

Within the *P.tristis* group, the basidiomata of *P.pinophila* can be recognised by their lack of hyphal cords and skeletal hyphae and their soft, yet rather firm and compact and ± elastic texture after drying, bluish to greenish colour of immature parts, narrow subicular hyphae, brown mature hymenium, long, laterally narrow and commonly star-shaped spores. *Pseudotomentellasciastra*, *P.pluriloba* and *P.media* can appear similar. Even though *P.sciastra* has star-shaped spores, it also has wider subicular hyphae than *P.pinophila* and, while *P.pluriloba* and *P.media* both share the characters of narrow hyphae, long spores and hymenia that are brown when mature with *P.pinophila*, they differ by having angular-nodulose spores, which are also laterally wider than the spores of *P.pinophila*.

##### Additional specimens studied.

NORWAY. Akershus: Asker, Skaugumåsen, boreonemoral, mixed forest on on soil with high pH, 23 September 2010, S. Svantesson (O F110327); Oslo (county): Oslo (municipality), Bygdøy, Hengsåsen, boreonemoral, mixed forest on soil with high pH, 22 September 2010, S. Svantesson (O F110328*); Oslo (county): Oslo (municipality), Gressholmen, boreonemoral, mixed forest on soil with high pH, 20 September 2010, S. Svantesson (O F110329); Telemark: Bamble, Rognsflaugane, boreonemoral, mixed forest on soil with high pH, 2 September 2010, K.-H. Larsson and S. Svantesson (O F110305); Akershus: Asker, Esvika, Løkenes, boreonemoral, mixed forest on soil with high pH, 15 August 2010, K.-H. Larsson and N. Svensson (O F110330*);

SWEDEN. Västergötland: Götene, Österplana, Hönsäter, coniferous forest on soil with high pH, 14 September 2017 S. Svantesson 418*, 419* (GB); Öland: Borgholm, Böda, Hagudden, mixed forest on soil with high pH, 5 October 2017 S. Svantesson 440* (GB).

#### Pseudotomentella pluriloba

Taxon classificationFungiThelephoralesThelephoraceae

Svantesson sp. nov.

MB829018

Fig. 13

##### Type.

FINLAND. Uusimaa: Loviisa, Rutosinpyhtää, Marinkylä, rotten trunk on the ground (*Picea*), 30 September 2010, U. Söderholm 4263 (holotype: H 6018127!, GenBank Acc. No. ITS: MK290698).

##### UNITE SH.

SH030565.07FU

##### Etymology.

The name refers to the several lobes of the spores.

##### Description.

**Basidiomata** annual, resupinate, membranaceous, effused to approximately ten centimetres in diameter. Mature parts continuous, with a cottony texture when fresh and a rather firm, fibrous and compact, yet quite soft and elastic texture when dried. Hymenium smooth, but sometimes strongly undulating; brown to purplish-brown when fresh, reddish-brown when dried. Immature parts discontinuous, byssoid with a cottony texture, both when fresh and when dried. Subhymenium and hymenium of immature parts blue when fresh, blue grey to brown grey after drying. Subiculum well developed, loose, fibrous, brown with an orange hue; forms the outer edge of basidiomata, extending noticeably beyond the hymenium.

**Figure 13.**
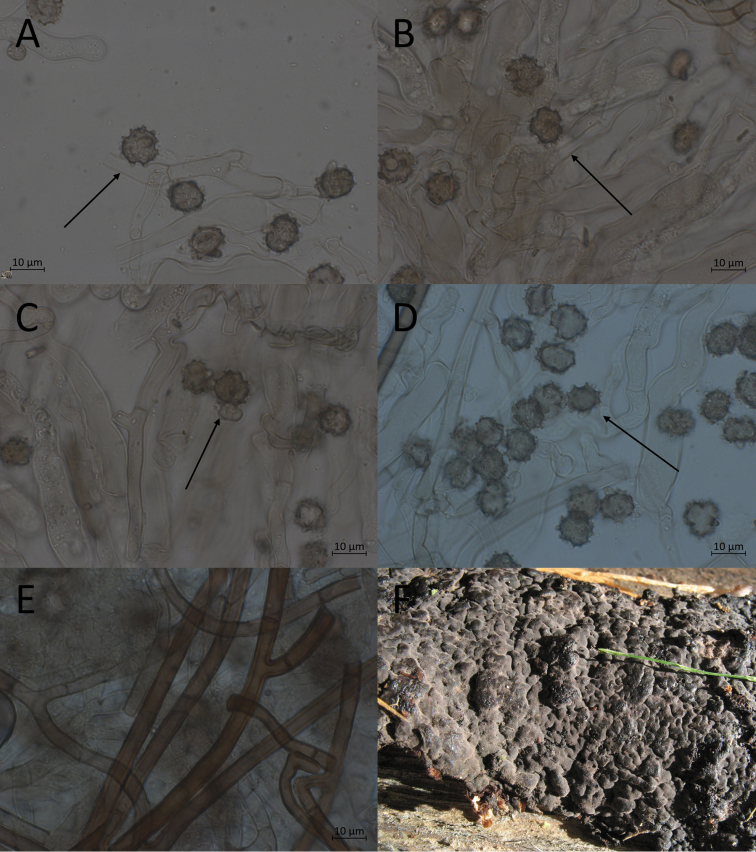
Morphological features of *P.pluriloba*, mounted in KOH and macroscopically. Holotype: **A, B, C** basidiospores in frontal face **D** in lateral face **E** subicular hyphae **F** mature basidiome.

**Hyphal cords** lacking, but loose bundles of subicular hyphae sometimes present.

**Hyphal system** monomitic, clamp connections absent from all hyphae.

**Subicular hyphae** noticeably long and straight, thick-walled; forming a loose tissue. Individual hyphae (3.9–) 4.0–5.9 (–6.8) μm wide, with a mean width of 6.8–5.1 μm; orange brown in both KOH and water.

**Subhymenial hyphae** often somewhat sinuous, thin to thick-walled; forming a rather dense tissue. Individual hyphae (2.7–) 2.9–5.3 (–5.4) μm wide, with a mean width of 4.0–4.2 μm; pale orange green to hyaline in KOH, blue green in the presence of air; pale orange green to hyaline in water, with strongly granular contents.

**Encrustation** granular, amyloid, concolourous with the hyphae in both KOH and water; usually common and scattered in occurrence on the upper parts of subhymenial hyphae and on the lower parts of basidia.

**Basidia** with four slightly curved sterigmata, occasionally two-sterigmate; clavate to narrowly clavate, sometimes clavopedunculate, thin-walled, with one-three slight constrictions. Dimensions: (55–) 58–87 (–94) × (10.3–) 10.7–13.3 (–13.4) μm; mean dimensions: 68–73 × 11.8–12.1 μm. Sterigmata (9.8–) 10.1–13.7 (–14.5) μm long, with a mean length of 11.5–12.3 μm. Colours and reactions the same as for the subhymenial hyphae, but in addition often with granular contents in KOH.

**Cystidial organs** lacking.

**Basidiospores** in frontal face generally with a subcircular basic shape and an angular to nodulose or sometimes cross-shaped outline, covered in bi- or trifurcate, sometimes singularly attached, echinuli. Nearly all spores with three-five distinct corners or rounded to square lobes; unlobed subcircular, unlobed subellipsoid or rounded, heart-shaped spores infrequently occurring, as well as abnormally large spores originating from two-sterigmate basidia. Frontal dimensions: (9.0–) 9.1–10.8 (–10.9) × (9.2–) 9.3–10.9 (–11.1) μm; mean dimensions: 9.8 × 10.2 μm; Q-value: 0.9–1.0 (–1.1); mean Q-value: 1.0. Echinuli (0.9–) 1.0–1.9 μm long, with a mean length of 1.4 μm. Lateral face ellipsoid, usually with evenly rounded edges, rarely with one-three lobes. Lateral dimensions: 9.0–10.4 (–10.8) × (6.7–) 6.8–8.5(8.6) μm; mean dimensions: 9.6–9.8 × 7.5–7.6 μm; Q-value: 1.2–1.4; mean Q-value: 1.3. Colour in KOH pale orange green, in the presence of air often with a pale blue green reaction; in water pale orange; occasionally amyloid.

**Chlamydospores** lacking.

##### Habitat.

Data on habitat are scarce to date, but recent Scandinavian collections have been made in mature to old coniferous or mixed forests on soil with intermediate pH. *Pseudotomentellapluriloba* has been found to form ectomycorrhiza with at least *Pseudotsugamenziesii* (Kõljalg et al. 2005, Nilsson et al. 2019).

##### Distribution.

Basidiomata encountered in: Finland and Sweden. Soil or root tip samples confirm presence also in: Canada and the United States.

##### Remarks.

Within the *P.tristis* group, the basidiomata of *P.pluriloba* are recognised by their lack of hyphal cords and skeletal hyphae and their soft, yet rather firm and compact and ± elastic texture after drying, bluish to greenish colour of immature parts, wide subicular hyphae and noticeably narrower subhymenial hyphae, long, moderately lobed spores and amyloid encrustation on subhymenial hyphae and basidia. *Pseudotomentellaabundiloba*, *P.alobata* and *P.media* can appear similar, but *P.media* differs by having smaller spores and narrower subicular hyphae which are ± the same width as its subicular hyphae, while *P.abundiloba* and *P.alobata* have frontally narrower spores with different lobation than *P.pluriloba*, as well as wider subicular hyphae and shorter sterigmata.

##### Additional specimens studied.

SWEDEN. Öland: Borgholm, Böda, Trollskogen, mixed forest on soil with intermediate pH, 5 October 2017, S. Svantesson 439* (GB).

#### Pseudotomentella rotundispora

Taxon classificationFungiThelephoralesThelephoraceae

Svantesson sp. nov.

MB829020

Fig. 14

##### Type.

SWEDEN. Västergötland: Götene, Medelplana, Eriksberg, boreonemoral, mixed forest on soil with high pH, 17 October 2016, S. Svantesson 413 (holotype: GB!, GenBank Acc. No. ITS: MK290674).

##### UNITE SH.

SH030562.07FU

##### Etymology.

The name refers to the appearance of the spores, which often have rounded or weakly pronounced lobes and comparably short echinuli.

##### Description.

**Basidiomata** annual, resupinate, membranaceous, effused – often to several tens of centimetres in diameter. Mature parts continuous, with a cottony texture when fresh and a rather firm, fibrous and compact, yet quite soft and elastic texture when dried. Hymenium smooth, but sometimes strongly undulating; brown to greenish-brown when fresh, concolourous when dried, but then sometimes with a red hue. Immature parts discontinuous, byssoid with a cottony texture, both when fresh and when dried. Subhymenium and hymenium of immature parts blue to blue green or grey when fresh, pale blue grey to grey blue when dried. Subiculum well developed, loose, fibrous, pale brown to pale orange brown; often forms the outer edge of basidiomata, extending noticeably beyond the hymenium.

**Figure 14.**
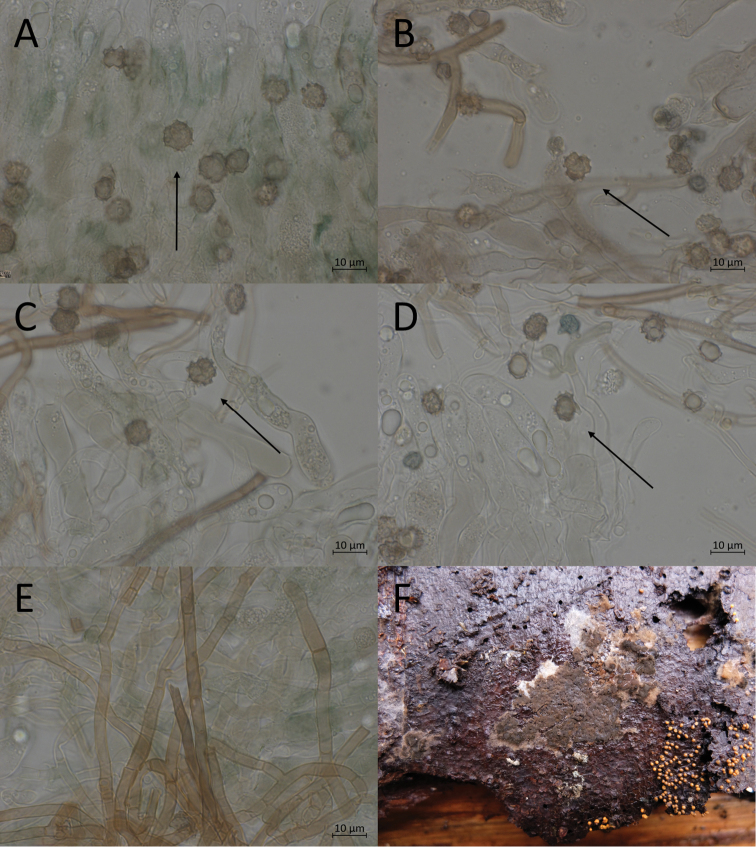
Morphological features of *P.rotundispora*, mounted in KOH and macroscopically. **A** (holotype) **B** (SS393) **C** (holotype) basidiospores in frontal face **D** in lateral face (holotype) **E** subicular hyphae (holotype) **F** mature basidiome (holotype).

**Hyphal cords** lacking, but loose bundles of subicular hyphae sometimes present.

**Hyphal system** monomitic, clamp connections absent from all hyphae.

**Subicular hyphae** noticeably long and straight, thick-walled; forming a loose tissue. Individual hyphae 3.0–4.4 (–4.6) μm wide, with a mean width of 3.4–3.8 μm; brown to orange brown in both KOH and water.

**Subhymenial hyphae** often somewhat sinuous, thin to thick-walled; forming a rather dense tissue. Individual hyphae (2.7–) 3.0–4.6 (–6.3) μm wide, with a mean width of 3.6–3.7 μm; pale brown to brown in KOH, often with orange or green hues, blue green in the presence of air; brown to orange brown in water, with strongly granular contents; some subhymenial hyphae with a pink colour in water and an amyloid reaction in Melzer’s reagent.

**Encrustation** granular, probably amyloid (hard to observe due to the colour); blackish in KOH, dark blue green in the presence of air; blackish in water; scattered in occurrence on the upper parts of subhymenial hyphae and on the lower parts of basidia.

**Basidia** with four slightly curved sterigmata, occasionally two-sterigmate; clavate or sometimes narrowly clavate or clavopedunculate, thin-walled, with one-three slight constrictions. Dimensions: 40–66 (–69) × 8.2–10.6 (–11.1) μm; mean dimensions: 54–60 × 8.8–9.7 μm. Sterigmata (6.6–) 7.4–11.0 (–11.5) μm long, with a mean length of 8.5–10.2 μm.

Colours and reactions the same as for the upper parts of subhymenial hyphae, but in addition often with granular contents in KOH.

**Cystidial organs** lacking.

**Basidiospores** in frontal face very variable. Generally with a subcircular basic shape and an angular, nodulose, star-shaped or occasionally cross-shaped outline, covered in bi- or trifurcate, sometimes singularly attached, echinuli. Nearly all spores with three-seven, commonly three or five, indistinct corners to distinct, usually rounded lobes; spores with angular or square lobes infrequently occurring, as well as abnormally large spores originating from two-sterigmate basidia. Frontal dimensions: (6.7–) 7.0–8.2 (–8.4) × 7.0–8.6 μm; mean dimensions: 7.5–7.6 × 7.7–7.9 μm; Q-value: 0.9–1.1; mean Q-value: 1.0. Echinuli 0.5–1.1 (–1.3) μm long, with a mean length of 0.8 μm. Lateral face ellipsoid, usually with evenly rounded edges, sometimes with one-three lobes. Lateral dimensions: 7.0–8.2 (–8.3) × (5.2–) 5.3–6.0 (–6.1) μm; mean dimensions: 7.6–7.9 × 5.6–5.7 μm; Q-value: 1.3–1.5; mean Q-value: 1.4. Colour in KOH pale brown to brown or pale orange brown to orange brown, in the presence of air sometimes with a blue green reaction; in water brown to orange brown; occasionally with an amyloid reaction.

**Chlamydospores** lacking.

##### Habitat.

Data on habitat are scarce to date, but recent Scandinavian collections have been made in old, coniferous, deciduous or mixed forests on soil with high pH. *Pseudotomentellarotundispora* has been found to form ectomycorrhiza with at least *Castanea* sp. and *Populustremula* (Kõljalg et al. 2005, Nilsson et al. 2019).

##### Distribution.

Basidiomata encountered in: Estonia, Norway and Sweden. Soil or root tip samples confirm presence also in: Austria, Italy and the United Kingdom.

##### Remarks.

Within the *P.tristis* group, the basidiomata of *P.rotundispora* can be recognised by their lack of hyphal cords and skeletal hyphae and their soft, yet rather firm and compact and ± elastic texture after drying, bluish to greenish colour of immature parts, narrow subicular hyphae and short spores. The other species within the group can appear similar, but have either wider hyphae, longer spores or both.

##### Additional specimens studied.

ESTONIA. Lääne: Hanila, Puhtu-Laelatu Nature Reserve, Puhtu peninsula, deciduous forest with *Populus*, *Tilia*, *Quercus* and *Picea*, 11 August 2005, U. Kõljalg (TU 100138*);

NORWAY. Oslo (county): Oslo (municipality), Bygdøy, Dronningberget, mixed forest on soil with high pH, 30 September 2017, K.-H. Larsson 17682* (O);

SWEDEN. Västergötland: Götene, Medelplana, Eriksberg, boreonemoral, mixed forest on soil with high pH, 17 October 2016, S. Svantesson 393*, 394* (GB).

#### Pseudotomentella sciastra

Taxon classificationFungiThelephoralesThelephoraceae

Svantesson & Kõljalg sp. nov.

MB829025

Fig. 15

##### Type.

SWEDEN. Småland: Jönköping, Svarttorp, Ramlaklint, boreonemoral, mixed, old-growth forest, on soil with intermediate pH, 12 September 2016, S. Svantesson 359 (holotype: GB!, GenBank Acc. No. ITS: MK290686).

##### UNITE SH.

SH030554.07FU

##### Etymology.

The name refers to the dark, star-like appearance of the spores.

##### Description.

**Basidiomata** annual, resupinate, membranaceous, effused – often to several tens of centimetres in diameter. Mature parts continuous, with a cottony texture when fresh and a rather firm, fibrous and compact, yet quite soft and elastic texture when dried. Hymenium smooth, but sometimes strongly undulating; blue grey when fresh and brown with a pinkish hue when dried. Immature parts discontinuous, byssoid with a cottony texture, both when fresh and when dried. Subhymenium and hymenium of immature parts blue grey when fresh or occasionally green or even yellow; blue grey to brown grey when dried. Subiculum well developed, loose, fibrous, pale orange brown to brown; often forms the outer edge of basidiomata, extending noticeably beyond the hymenium.

**Figure 15.**
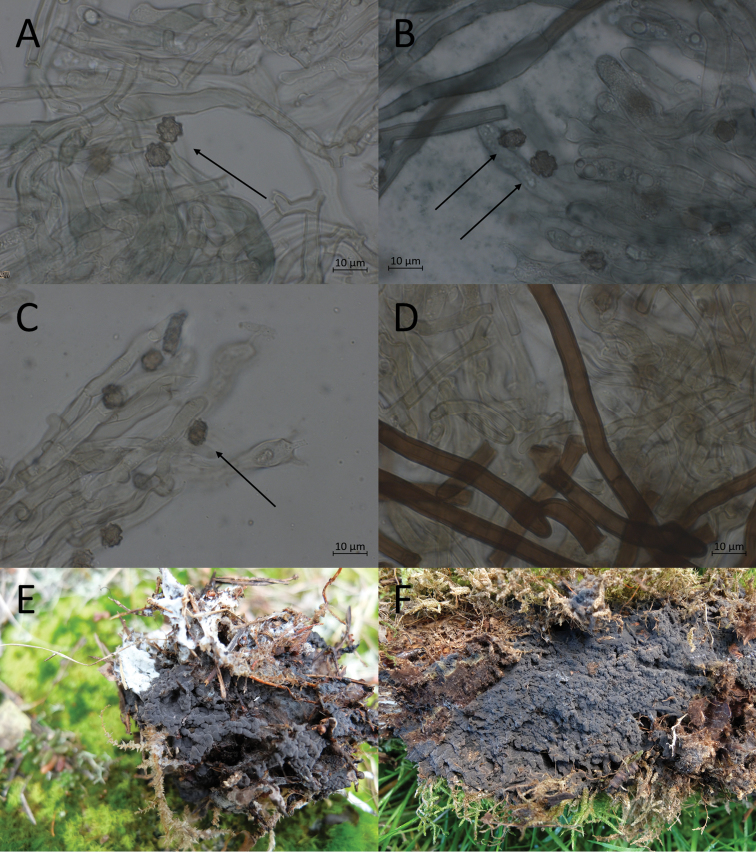
Morphological features of *P.sciastra*, mounted in KOH and macroscopically. **A** basidiospore in frontal face (O F110317) **B** in frontal and lateral faces (O F110317) **C** in lateral face (O F110317) **D** subicular hyphae (O F110317) **E** (SS420) **F** (SS312) mature basidiomata.

**Hyphal cords** lacking, but loose bundles of subicular hyphae sometimes present.

**Hyphal system** monomitic, clamp connections absent from all hyphae.

**Subicular hyphae** noticeably long and straight, thick-walled; forming a loose tissue. Individual hyphae (3.9–) 4.4–6.6 (–6.8) μm wide, with a mean width of 5.0–5.8 μm; brown to orange brown in KOH, orange brown in water.

**Subhymenial hyphae** often somewhat sinuous, thin to thick-walled; forming a rather dense tissue. Individual hyphae 2.9–5.0 (–6.0) μm wide, with a mean width of 3.8–4.0 μm; hyaline to pale green in KOH, blue green in the presence of air; pale orange green to pale yellowish-green in water, with strongly granular contents.

**Encrustation** granular, probably amyloid (hard to observe due to the colour); blackish-brown in KOH, dark blue green in the presence of air; blackish-brown in water; scattered in occurrence on the upper parts of subhymenial hyphae and on the lower parts of basidia.

**Basidia** with four slightly curved sterigmata, occasionally two-sterigmate; clavate to narrowly clavate, sometimes clavopedunculate, thin-walled, with one-three slight constrictions. Dimensions: 42–67 (–68) × 7.3–9.0 (–9.3) μm; mean dimensions: 54–55 × 7.8–8.1 μm. Sterigmata (6.0–) 6.3–8.9 (–9.1) μm long, with a mean length of 7.4–7.9 μm. Colours and reactions the same as for the subhymenial hyphae, but in addition often with granular contents in KOH.

**Cystidial organs** lacking.

**Basidiospores** in frontal face with a subcircular basic shape and a star- or cross-shaped, sometimes angular to nodulose outline, covered in bi- or trifurcate, sometimes singularly attached, echinuli. Nearly all spores with four-six distinct, rounded to more often square lobes or rarely corners; abnormally large spores originating from two-sterigmate basidia infrequently occurring. Frontal dimensions: (6.0–) 6.1–7.9 (–8.1) × 6.3–8.2 μm; mean dimensions: 6.6–7.3 × 6.7–7.7 μm; Q-value: 0.9–1.1; mean Q-value: 1.0. Echinuli (0.5–) 0.6–1.2 (–1.4) μm long, with a mean length of 0.8–0.9 μm. Lateral face ellipsoid to ovoid, with evenly rounded edges or one-three lobes. Lateral dimensions: (6.2–) 6.5–7.7 (–8.0) × (4.3–) 4.4–6.0 (–6.2) μm; mean dimensions: 6.8–7.3 × 4.6–5.4 μm; Q-value: 1.2–1.6 (–1.7); mean Q-value: 1.3–1.5. Colour in KOH brown to yellow brown, in the presence of air often with a green to blue green reaction; in water pale greenish to pale greenish-orange; occasionally amyloid.

**Chlamydospores** lacking.

##### Habitat.

Recent Scandinavian collections have been made in mature to old coniferous, deciduous or mixed forests on soil with intermediate to high pH. *Pseudotomentellasciastra* has been found to form ectomycorrhiza with at least *Castaneasativa*, *Cedruslibani*, *Neottiaovata*, *Piceaabies* and *Quercus* sp. (Kõljalg et al. 2005, Nilsson et al. 2019).

##### Distribution.

Basidiomata encountered in: Estonia, Finland, Norway, Sweden, Turkey and the United Kingdom. Soil or root tip samples confirm presence also in: the Czech Republic, Mexico, Portugal (Madeira) and the United States.

##### Remarks.

All studied European specimens previously identified as *P.atrofusca* belong to *P.sciastra*. The two species display considerable morphological differences (see key).

Within the *P.tristis* group, the basidiomata of *P.sciastra* are recognised by their lack of hyphal cords and skeletal hyphae and their soft, yet rather firm and compact and ± elastic texture after drying, bluish to greenish colour of immature parts, wide subicular hyphae and small, star-shaped spores. *Pseudotomentellapinophila* is similar, but has narrower subicular hyphae and larger spores.

##### Additional specimens studied.

ESTONIA. Ida-Virumaa: Illuka, Puhatu Nature Reserve, Poruni primeval forest, wetlands, 1 October 2006, U. Kõljalg (TU 100644*); [Saare,] Saarema, Kihelkonna, Hülgera, Sampling area G4422, 25 September 2015, A. Saitta (TU 124211*, TU 124213*);

FINLAND. Etelä-Häme: Jyväskylä, Korpilahti, Oittila, on dead trunk of *Ulmusglabra*, 3 September 2014, U. Söderholm 4755 (H 6052710); Kanta-Häme: Lammi, Lammi Biological Station, Leib-rich forest, 12 September 2001, K.-H. Larsson (TU 108754*);

NORWAY. Oslo (county): Oslo (municipality), Bygdøy, Hengsåsen, boreonemoral, mixed forest on soil with high pH, 16 August 2010, K.-H. Larsson and N. Svensson (O F110317*); Østfold: Moss, Jeløya, boreonemoral, mixed forest on soil with high pH, 26 September 2010, S. Svantesson and N. Svensson (O F110318*); Oppland: Dovre, Grimsdalen, Austre Stakkstosætra, *Pinussylvestris* forest, 26 August 2010, K.-H. Larsson and S. Svantesson (O F110301); Vestfold: Larvik, Kvelde, Jordstøyp, boreonemoral, mixed forest on soil with intermediate pH, 1 October 2010, K.-H. Larsson (O F110302); Ibidem, on soil with high pH, 1 September 2010, K.-H. Larsson and S. Svantesson (O F110303, F110304); Aust-Agder, Risør, Glupedalen, boreonemoral, mixed forest on soil with high pH, 10 September 2010, S. Svantesson and N. Svensson (O F110319, F110320, F110321); Aust-Agder: Tvedestrand, Eidbo, boreonemoral, mixed forest on soil with intermediate pH, 10 September 2010, S. Svantesson and N. Svensson (O F110322*); Oslo (county): Oslo (municipality), Gressholmen, boreonemoral, mixed forest on soil with high pH, 20 September 2010, S. Svantesson (O F110323, F110324); Oslo (county): Oslo (municipality), Killingen, boreonemoral, mixed forest on soil with high pH, 22 September 2010, S. Svantesson (O F110325); Buskerud: Ringerike, Ulltveit Nature Reserve, boreonemoral, coniferous forest on soil with high pH, 25 September 2010, S. Svantesson and N. Svensson (O F110326);

SWEDEN. Småland: Jönköping, Svarttorp, Ramlaklint, boreonemoral, mixed, old-growth forest, on soil with intermediate pH, 12 September 2016, S. Svantesson 360 (GB); Bohuslän: Tanum (municipality), Tanum (parish), Lammön, boreonemoral, deciduous forest on soil with high pH, 6 September 2016, S. Svantesson 312* (GB); Västergötland: Göteborg, Askim, Årekärrslunden, 24 October 2015, K.-H. Larsson 17308b* (GB); Dalsland, Mellerud, Skållerud, Österbo, mixed forest on soil with high pH, 20 September 2017, S. Svantesson 420* (GB); Ibidem, Norgekullen SW, coniferous forest on soil with high pH, 20 September 2017, S. Svantesson 423* (GB);

TURKEY. [Antalya: Elmalı,] Ciglikara, 2009, L. Tedersoo (TU 110153*); [Isparta:] Yukan-Gökdere [=Yukarı Gökdere], 2009, L. Tedersoo (TU 110113*);

UNITED KINGDOM. Scotland, Aberdeenshire: Inverurie, Burnhervie, in a small group of planted *Populus* trees, 16 September 2005, I. J. Alexander (TAA 187322*).

#### Pseudotomentella tristis

Taxon classificationFungiThelephoralesThelephoraceae

(P. Karst.) M.J.Larsen, Nova Hedwigia 22(1–2): 613 (1971) [1972]

Fig. 16

##### Homotypic names.

Hypochnussubfuscusssp.tristis P.Karst., Meddeland. Soc. Fauna Fl. Fenn. 9: 71 (1883). *Hypochnustristis* (P.Karst.) P.Karst. Bidrag Kännedom Finlands Natur Folk. 48: 440 (1889). *Tomentellatristis* (P.Karst.) Höhn. & Litsch., Sitzungsber. Kaiserl. Akad. Wiss., Wien. Math.-Naturwiss. Cl., Abt. 1 115: 1572 (1906). Type. FINLAND. Tavastia australis [= Etelä-Häme]: Tammela, Mustiala, ad Betulam, 19 August 1865, P.A. Karsten (lectotype: Herbarium P. A. Karsten 3036 [H 6018703]!, designated by M.J. Larsen in Nova Hedwigia 22(1–2): 613 (1971) [1972]); SWEDEN. Västerbotten: Vännäs, Orrböle, boreal, mixed forest on soil with high pH, 28 August 2015, S. Svantesson 193 (EPITYPE: GB!, here designated, MycoBank Typification No. MBT384911, GenBank Acc. No. ITS: MK290679).

**Figure 16.**
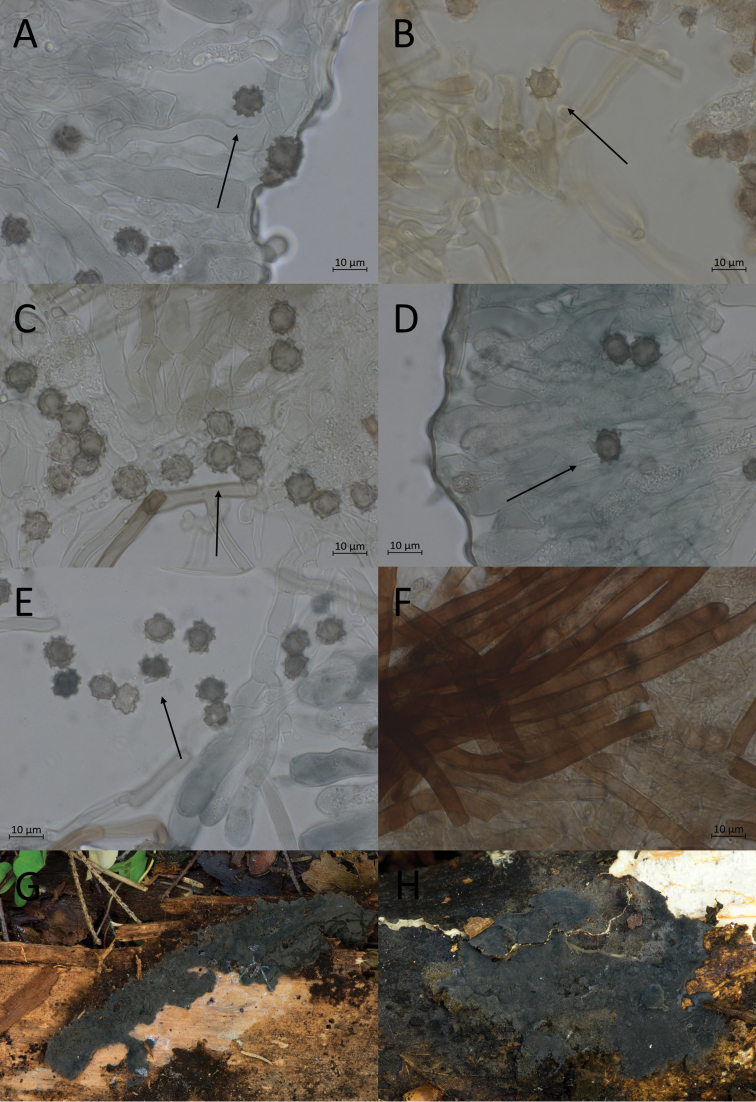
Morphological features of *P.tristis*, mounted in KOH and macroscopically. A (LK54/13) **B** (lectotype) **C** (epitype), basidiospores in frontal face **D** (epitype) **E** (TAA 159485) in lateral face **F** subicular hyphae (epitype) **G** young basidiome (TU 115439) **H** mature basidiome (TU 115642).

##### Heterotypic names.

***Hypochnopsisfuscata*** P.Karst., Bidrag Kännedom Finlands Natur Folk 48: 443 (1889). *Hypochnusfuscatus* (P.Karst.) Sacc., Syll. fung. 9: 244 (1891). Type. FINLAND. Tavastia australis: Messuby [Tavastia australis = Etelä-Häme; Messuby is part of the city of Tampere], September 1860, P.A. Karsten (lectotype: Herbarium P.A. Karsten 770 [H 6059014]!, designated by M.J. Larsen in Nova Hedwigia 22(1–2): 616 (1971) [1972]); SWEDEN. Västerbotten: Vännäs, Orrböle, boreal, mixed forest on ground with high pH, 28 August 2015, S. Svantesson 193 (EPITYPE: GB!, here designated, MycoBank Typification No. MBT384955, GenBank Acc. No. ITS: MK290679).

#### Hypochnus sitnensis

Taxon classificationFungiThelephoralesThelephoraceae

Bres., Atti Imp. Regia Accad. Roveretana. 3(1): 115 (1897)

##### Type.

SLOVAKIA [Hungary at the time of collection]. Prenčow, Sitno, infra filagorum, in trunco putr. Fagi, 11 September 1895, Andr. Kmet (holotype: S F15178!).

##### UNITE SH.

SH030560.07FU

##### Description.

**Basidiomata** annual, resupinate, membranaceous, effused – often to several tens of centimetres in diameter. Mature parts continuous, with a cottony texture when fresh and a rather firm, fibrous and compact, yet quite soft and elastic texture when dried. Hymenium smooth, but sometimes strongly undulating; blue grey to purplish-brown when fresh, blue grey or blue-greenish grey to brown, with a reddish hue, when dried. Immature parts discontinuous, byssoid with a cottony texture, both when fresh and when dried. Subhymenium and hymenium of immature parts blue green, blue or blue grey when fresh and pale grey blue or pale blue grey to grey blue or blue grey when dried, sometimes with a green hue. Subiculum well developed, loose, fibrous, orange brown; often forms the outer edge of basidiomata, extending noticeably beyond the hymenium.

**Hyphal cords** lacking, but loose bundles of subicular hyphae sometimes present.

**Hyphal system** monomitic, clamp connections absent from all hyphae.

**Subicular hyphae** noticeably long and straight, thick-walled; forming a loose tissue. Individual hyphae (4.5) 4.6–7.4 μm wide, with a mean width of 5.4–6.2 μm; orange brown to dark brown in KOH, orange brown to brown in water.

**Subhymenial hyphae** often somewhat sinuous, thin to thick-walled; forming a rather dense tissue. Individual hyphae 3.2–6.2 (7.2) μm wide, with a mean width of 3.9–4.5 μm; pale orange brown to pale green in KOH, blue green in the presence of air; pale green to pale greenish-orange in water, with strongly granular contents.

**Encrustation** granular, probably amyloid (hard to observe due to the colour); blackish in KOH, dark blue green in the presence of air; blackish in water; scattered in occurrence on the upper parts of subhymenial hyphae and on the lower parts of basidia.

**Basidia** with four slightly curved sterigmata, occasionally two-sterigmate; clavate to narrowly clavate, sometimes clavopedunculate, thin-walled, with one-three slight constrictions. Dimensions: 51–76 (–84) × (8.1) 8.3–13.7 (–14.6) μm; mean dimensions: 56–62 × 9.6–11.6 μm. Sterigmata (8.0) 8.3–11.3 (13.3) μm long, with a mean length of 9.4–10.2 μm. Colours and reactions the same as for the subhymenial hyphae, but in addition often with granular contents in KOH.

**Cystidial organs** lacking.

**Basidiospores** in frontal face generally with a subcircular basic shape and an unlobed, angular, weakly nodulose or sometimes cross-shaped outline, covered in bi- or trifurcate, occasionally singularly attached echinuli. A majority of the spores normally unlobed or with three-five indistinct corners to rounded lobes; subcircular spores with more pronounced, sometimes square lobes or ovoid to subellipsoid spores also common in some specimens; subcircular, six-lobed spores infrequently occurring, as well as abnormally large spores originating from two-sterigmate basidia. Frontal dimensions: 7.7–9.1 (9.2) × 8.0–9.2 (9.6) μm; mean dimensions: 8.3–8.6 × 8.4–8.8 μm; Q-value: 0.9–1.1; mean Q-value: 1.0–1.1. Echinuli (0.8) 0.9–1.9 μm long, with a mean length of 1.4 μm. Lateral face ellipsoid to narrowly ovoid or sometimes semicircular in shape, usually with evenly rounded edges, sometimes with one-three lobes. Lateral dimensions: (7.7) 8.0–9.0 × (6.0) 6.1–6.8 (7.0) μm; mean dimensions: 8.3–8.5 × 6.3–6.5 μm; Q-value: 1.2–1.4 (–1.5); mean Q-value: 1.3. Colour in KOH brown to orange brown, in the presence of air often with a blue green reaction; in water greenish-orange to orange brown; occasionally amyloid.

**Chlamydospores** lacking.

##### Habitat.

Data on habitat are scarce to date, but recent Scandinavian collections have been made in mature to old deciduous or mixed forests on soil with intermediate to high pH. *Pseudotomentellatristis* has been found to form ectomycorrhiza with at least *Betulapendula* and *Fagussylvatica* (Kõljalg et al. 2005, Nilsson et al. 2019).

##### Distribution.

Basidiomata encountered in: Estonia, Finland, Norway, Slovakia, Slovenia and Sweden. Soil or root tip samples confirm presence also in: Germany.

##### Remarks.

We here select a Swedish specimen to serve as an epitype for both *P.tristis* and *H.fuscata*. This decision is based on four reasons: first, the present study has found *P.tristis* and *H.fuscata* to be conspecific; secondly, the lectotypes of these species were both collected in Finland (within the same county); thirdly we have found *P.tristis* to occur at several localities in both Finland and Sweden; and fourthly, the Swedish material chosen is both ampler and displays more variation with respect to maturity of the basidiome than the single available recent Finnish collection.

The type specimen of *H.sitnensis* was collected in Slovakia, i.e. far from the type locality of *P.tristis*. It displays the morphological characters of *P.tristis*, apart from the absence of an amyloid reaction in the encrusting material found on basidia and subhymenial hyphae. This might be an artefact of, for example, its drying conditions, time or intraspecific variation, but since the specimen is in too poor a condition to allow DNA sequencing with currently available methods, this cannot be ascertained. We therefore consider it a synonym of *P.tristis* and suggest it be epitypified in due course with locally sampled material that matches the type.

In the case of *H.sitnensis*, there is only one collection matching the locality and habitat description of the protologue as well as the collector stated. It predates the publication of the species. This collection must hence be regarded as a holotype.

P.A. Karsten 770 is the lectotype of *H.fuscata*, as designated by Larsen in Nova Hedwigia (1971), but his note has been placed in P.A. Karsten 769, which has created confusion amongst mycologists studying these specimens. Mature spores that fall within the morphological span of *P.tristis* can easily be found in all collections that match Karsten’s (1889) description of the species, with respect to locality and date. It would hence seem that the smooth, small, bluish spores he writes of in the protologue (see Introduction) probably were immature ones, studied in the presence of air.

Within the *P.tristis* group, basidiomata of *P.tristis* itself can be recognised by their lack of hyphal cords and skeletal hyphae and their soft, rather elastic texture after drying, bluish to greenish colour of immature parts, wide subicular hyphae, medium sized, commonly angular to nodulose spores and relatively long echinuli and sterigmata. *Pseudotomentellatristoides* is similar but has shorter echinuli and sterigmata, *P.sciastra* has smaller, star-shaped spores and *H.rhacodium* (only known from the type) has hard, brittle basidiomata after drying.

##### Additional specimens studied.

ESTONIA. Valga: Otepää, Kääriku, Välkjärve, 10 September 2012, U. Kõljalg (TU 115439*); Tartumaa: Võnnu, Terikeste, on fallen branch of *Piceaabies* in mixed forest, 20 August 1996, U. Kõljalg (TAAM 159485*); Lääne: Vormsi, road from Diby to Norrby, deciduous forest with *Betula* and *Corylus*, 27 September 2008, U. Kõljalg (TU 108134*);

FINLAND. Kanta-Häme: Lammi, Lammi Biological Station, Leib-rich forest, 12 September 2001, U. Kõljalg (TU 108757*); Satakunta: Luvia, Säppi, on fallen decayed *Betula*, 11 September 2013, L. Kosonen 54/13* (TUR);

NORWAY. Møre og Romsdal: Nesset, Eikesdal, Ljåstranda, rich, deciduous forest, 18 September 2011, K.-H. Larsson 15084* (O); Oppland: Vinstra, Liadalen, rich, deciduous forest, 24 September 2013, K.-H. Larsson 16367* (O); Hedmark: Ringsaker, Liberget, 24 August 1984, K.-H. Larsson 5901 (GB 87563); Sogn og Fjordane: Stryn, Flostranda Nature Reserve, boreonemoral, deciduous forest on ground with high pH, 25 September 2013, K.-H. Larsson (O F110297*); Rogaland: Forsand, Rössdalen, boreonemoral, deciduous forest on ground with high pH, 29 September 2012, K.-H. Larsson and S. Svantesson (O F110298*); Oppland: Nord-Fron, Liadalane Nature Reserve, boreonemoral, deciduous forest on ground with intermediate pH, 24 September 2013, K.-H. Larsson (O F110299, F110300*);

SLOVENIA. Upravna enota Kočevje: Rahjenavski Rog virgin forest reserve, S and E edge of the reserve, beech-silver fir old growth forest, 21 September 2012, S. Kõljalg; U. Kõljalg (TU 115642*);

SWEDEN. Västerbotten: Vännäs (municipality), Vännäs (parish), Orrböle, boreal, mixed, secondary, mature forest, on ground with high pH, 28 August 2015, S. Svantesson 188 (GB); Dalsland: Ödskölt, S of lake Ivägsjön, on deciduous wood, 22 September 1990, K. Hjortstam 17197 (K.-H. Larsson private collection).

#### Pseudotomentella tristoides

Taxon classificationFungiThelephoralesThelephoraceae

Svantesson & K.H.Larss. sp. nov.

MB829030

Fig. 17

##### Type.

NORWAY. Nord-Tröndelag: Snåsa, Bergsåsen, boreal, deciduous forest on soil with intermediate pH, 28 August 2012, K.-H. Larsson (holotype: O F110306!, GenBank Acc. No. ITS: MK290692).

##### UNITE SH.

SH030566.07FU

##### Etymology.

The name refers to the overall similarity between this species and *P.tristis*.

##### Description.

**Basidiome** annual, resupinate, membranaceous, effused to approximately ten centimetres in diameter. Mature parts continuous, with a rather firm, fibrous and compact, yet quite soft and elastic texture. Hymenium smooth; brown with a reddish hue. Immature parts discontinuous, byssoid with a cottony texture. Subhymenium and hymenium of immature parts initially pale greyish-blue, when more mature dark blue grey. Subiculum well-developed, loose, fibrous, brown with an orange hue; forms the outer edge of the basidiome, extending noticeably beyond the hymenium. All characters recorded in dried state.

**Figure 17.**
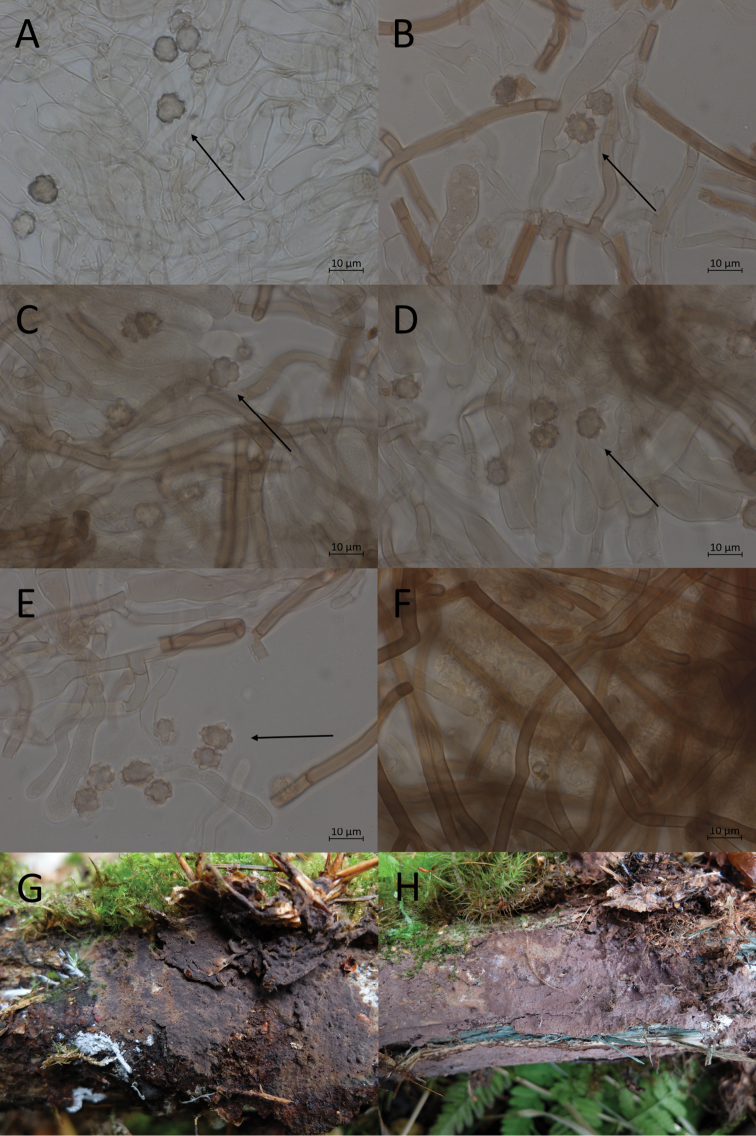
Micromorphological features of *P.tristoides* in KOH. Holotype: **A, B, C** basidiospores in frontal face **D, E** in lateral face **E** subicular hyphae.

**Hyphal cords** lacking, but loose bundles of subicular hyphae sometimes present.

**Hyphal system** monomitic, clamp connections absent from all hyphae.

**Subicular hyphae** noticeably long and straight, thick-walled; forming a loose tissue. Individual hyphae (4.7–) 4.9–7.1 (–7.6) μm wide, with a mean width of 6.0 μm; orange brown to dark brown in KOH, orange brown to brown in water.

**Subhymenial hyphae** often somewhat sinuous, thin to thick-walled; forming a rather dense tissue. Individual hyphae (3.1–) 3.2–5.3 (–5.4) μm wide, with a mean width of 4.6 μm; pale yellowish-brown in KOH, pale green to blue green in the presence of air; pale green to pale orange green in water, with strongly granular contents.

**Encrustation** granular, amyloid, concolourous with the hyphae in both KOH and water; scattered in occurrence on the upper parts of subhymenial hyphae and on the lower parts of basidia.

**Basidia** with four slightly curved sterigmata, occasionally two-sterigmate; clavate to narrowly clavate, sometimes clavopedunculate, thin-walled, with one-three slight constrictions. Dimensions: (49–) 54–72 (–75) × (7.3–) 7.9–10.0 (–10.5) μm; mean dimensions: 63 × 9.1 μm. Sterigmata (7.6–) 7.8–9.9 (–10.5) μm long, with a mean length of 8.6 μm. Colours and reactions the same as for the subhymenial hyphae.

**Cystidial organs** lacking.

**Basidiospores** in frontal face generally with a subcircular basic shape and an angular to nodulose or sometimes cross-shaped outline, covered in bi- or trifurcate, sometimes singularly attached, echinuli. A majority of the spores with three-five indistinct corners to distinct, square lobes; subellipsoid, ovoid and subcircular spores with a rather evenly rounded outline occasionally occurring, as well as subcircular, six-lobed spores; abnormally large spores originating from two-sterigmate basidia infrequently seen. Frontal dimensions: 7.7–8.6 (–8.8) × (7.4–) 7.7–9.3 (–9.5) μm; mean dimensions: 8.2 × 8.5 μm; Q-value: 0.9–1.1; mean Q-value: 1.0. Echinuli (0.5–) 0.7–0.9 (–1.1) μm long, with a mean length of 0.8 μm. Lateral face ellipsoid, usually with evenly rounded edges, sometimes with one-three lobes. Lateral dimensions: (7.9–) 8.0–8.6 × 6.0–6.5 (–6.7) μm; mean dimensions: 8.2 × 6.3 μm; Q-value: 1.2–1.4; mean Q-value: 1.3. Colour in KOH brown to yellow brown, in the presence of air often with a green to blue green reaction; in water pale greenish to pale greenish-orange; occasionally amyloid.

**Chlamydospores** lacking.

##### Habitat.

The only specimen recorded to date of *P.tristoides* is the type collection, which was obtained in an old, mixed forest on soil with intermediate pH. UNITE sequence metadata show that the species forms ectomycorrhiza with at least *Populusalba* and *Cephalantheradamasonium* (Kõljalg et al. 2005, Nilsson et al. 2019).

##### Distribution.

Basidiomata encountered in: Norway. Soil or root tip samples confirm presence also in: Estonia and the Czech Republic.

##### Remarks.

Within the *P.tristis* group, the basidiome of *P.tristoides* can be recognised by its lack of hyphal cords and skeletal hyphae and its soft, yet rather firm and compact and ± elastic texture after drying, bluish to greenish colour of immature parts, wide subicular hyphae, medium sized, angular-nodulose spores and relatively short echinuli and sterigmata. *Pseudotomentellatristis* is similar but has longer echinuli and sterigmata, *P.sciastra* has smaller, star-shaped spores and *H.rhacodium* (only known from the type) has hard, brittle basidiomata after drying.

#### Pseudotomentella umbrina

Taxon classificationFungiThelephoralesThelephoraceae

(Fr.) M.J.Larsen, Canad. J. Bot. 45: 1298 (1967)

Fig. 18

##### Homotypic names.

*Thelephoraumbrina* Fr. Elench. fung. 1: 199 (1828), non Pers. (1801), sanctioned name [Fries explicitly excluded *T.umbrina* Pers. from his concept]. *Hypochnusumbrinus* (Fr.) Fr. [basionym not cited], Summa veg. Scand.: 337 (1849), non Wallr. (1833), illegitimate name [combination also made by Quélet (1888) and Burt (1916)]. *Corticiumumbrinum* (Fr.) Fr., Hymenomyc. eur.: 658 (1874). *Coniophoraumbrina* (Fr.) Sacc., Syll. fung. 6: 652 (1888) [as (Alb. & Schwein.) Fr.]. *Tomentellaumbrina* (Fr.) Litsch., Bull. Soc. Mycol. France. 49(1): 52 (June 20, 1933) [combination also made by Donk, Meded. Bot. Mus. Herb. Rijks Univ. Utrecht. 9: 29 (before July 7 1933)]. *Prillieuxiaumbrina* (Fr.) Park.-Rhodes 1956 Ann. Bot. (Oxford). 20(78): 258. 1956, invalid name, basionym not cited. *Tomentellastrumumbrinum* (Fr.) Svrček, Ceská Mykol. 12(2): 70 (1958).

##### Type.

SWEDEN. Småland: Femsjö, E. Fries (neotype: Herb. Fries [UPS F003106]!, designated by E.A. Burt in Ann. Missouri Bot. Gard 3: 213 (1916)); Småland: Hylte, Femsjö, Femsjö Church Nature Reserve, boreonemoral, mixed forest on soil with intermediate pH, 7 September 2016, S. Svantesson 351 (EPITYPE: GB!, here designated, MycoBank Typification No. MBT384818, GenBank Acc. No. ITS: MK290700).

##### UNITE SH.

SH030549.07FU

##### Description.

**Basidiomata** annual, resupinate, membranaceous, effused – often to several tens of centimetres in diameter. Mature parts continuous, with a cottony texture when fresh and a rather firm, fibrous and compact, yet quite soft and elastic texture when dried. Hymenium smooth, but sometimes strongly undulating; blue grey or purplish-grey to pale brown or brown when fresh, pale brown to brown when dried, sometimes with a reddish or greyish hue. Immature parts discontinuous, byssoid with a cottony texture, both when fresh and when dried. Subhymenium and hymenium of immature parts pale blue grey or pale purplish-grey to pale brown when fresh, pale brown when dried. Subiculum well developed, loose, fibrous, orange brown; often forms the outer edge of basidiomata, extending noticeably beyond the hymenium.

**Figure 18.**
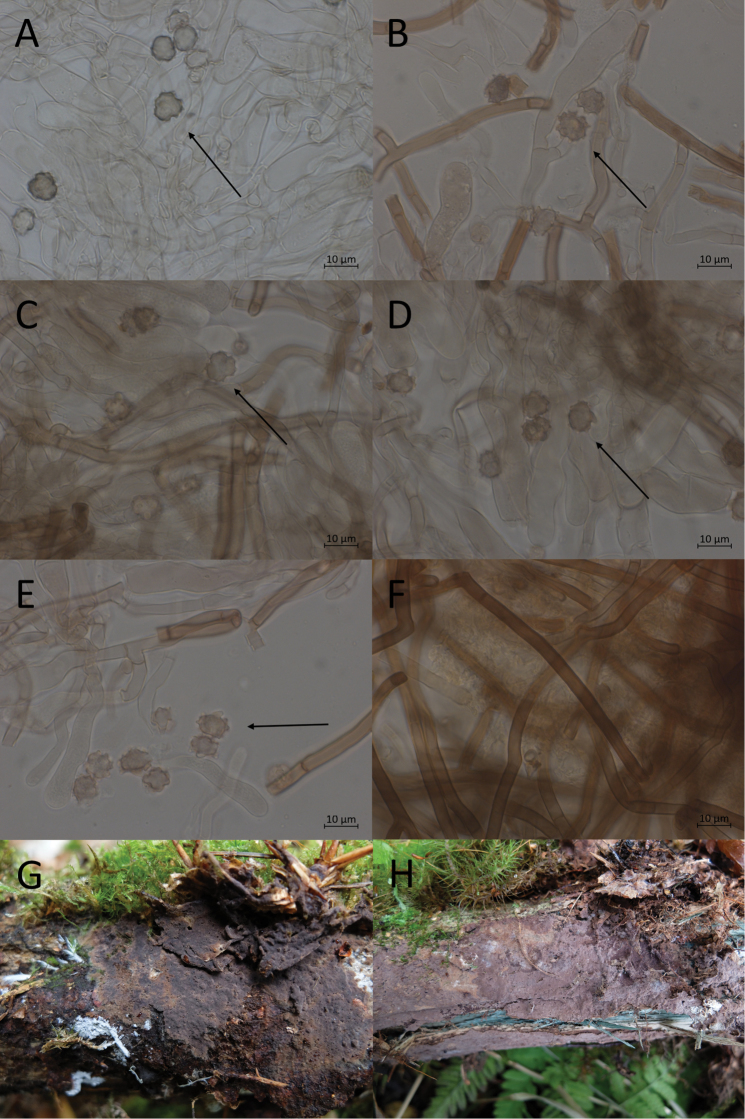
Morphological features of *P.umbrina*, mounted in KOH and macroscopically. **A** (O F110268) **B** (epitype) **C** (epitype) basidiospores in frontal face **D, E** in lateral face (epitype) **F** subicular hyphae (epitype) **G** (epitype) **H** (SS174) mature basidiomata.

**Hyphal cords** lacking, but loose bundles of subicular hyphae sometimes present.

**Hyphal system** monomitic, clamp connections and reaction in Melzer’s reagent absent from all hyphae.

**Subicular hyphae** noticeably long and straight, thick-walled; forming a loose tissue. Individual hyphae 3.3–4.8 (–5.3) μm wide, with a mean width of 4.0–4.3 μm; orange brown to brown in KOH, orange brown in water.

**Subhymenial hyphae** often somewhat sinuous, thin to thick-walled; forming a rather dense tissue. Individual hyphae (2.8–) 2.9–5.0 (–5.9) μm wide, with a mean width of 3.7–4.2 μm; in the upper parts, pale green in KOH, sometimes with a faintly blue or brown hue; in the lower parts, orange brown to brown; in water, orange brown, with strongly granular contents.

**Encrustation** lacking.

**Basidia** with four slightly curved sterigmata, occasionally two-sterigmate; clavate to narrowly clavate, sometimes clavopedunculate, thin-walled, with one-three slight constrictions. Dimensions: (54–) 57–71 (–77) × (8.3–) 8.5–10.9 (–12.4) μm; mean dimensions: 60–64 × 9.5–10.3 μm. Sterigmata (8.7–) 8.8–11.1 (–11.7) μm long, with a mean length of 9.6–10.5 μm. Colour for the great majority very pale green in KOH, sometimes with a faintly blue or brown hue (but not the blue green reaction present in other species), for a small number formed directly from subicular hyphae brown; sometimes with granular contents; in water orange brown and with strongly granular contents.

**Cystidial organs** lacking.

**Basidiospores** in frontal face generally with a broadly subellipsoid, triangular or subcircular basic shape and an unlobed, angular, nodulose or sometimes cross-shaped outline, covered in bi- or trifurcate, occasionally singularly attached, echinuli. A majority of the spores normally with three-six indistinct corners to distinct, square lobes; broadly ellipsoid, unlobed spores infrequently occurring (but dominate in some collections), as well as abnormally large spores originating from two-sterigmate basidia. Frontal dimensions: 7.7–9.3 (–9.4) × (7.6–) 7.9–9.1 (–9.4) μm; mean dimensions: 8.3–8.7 × 8.4–8.7 μm; Q-value: (0.9–) 1.0–1.1; mean Q-value: 1.0. Echinuli (0.7–) 0.8–1.5 μm long, with a mean length of 1.1–1.2 μm. Lateral face ellipsoid to narrowly ovoid or sometimes semicircular in shape, usually with evenly rounded edges, sometimes with one-three lobes. Lateral dimensions: 8.0–9.3 (–9.6) × (5.1–) 5.6–6.7 (–6.9) μm; mean dimensions: 8.4–8.7 × 6.0–6.1 μm; Q-value: (1.2–) 1.3–1.6 (–1.7); mean Q-value: 1.4–1.5. Colour in KOH pale green to pale brown; in water orange brown to brown; inamyloid.

**Chlamydospores** lacking.

##### Habitat.

*P.umbrina* has a wide ecological amplitude. Recent Scandinavian collections have been made in young to old deciduous, mixed and coniferous forests on soil with low to high pH, as well as on the tundra. The species has been found to form ectomycorrhiza with at least *Abiesalba*, *Alnusrubra*, *Betulanana*, Betulapubescensssp.czerepanovii, Betulapubescensssp.pubescens, *Dryasoctopetala*, *Fagussylvatica*, *Piceaabies*, *Piceaglauca*, *Piceamariana*, *Pinusbanksiana*, *Pinuspinaster*, *Pinussylvestris*, *Pseudotsugamenziesii*, *Pyrolamedia*, *Quercuspetraea*, *Salixpolaris* and *Tsugacanadensis* (collection data; Kõljalg et al. 2005, Nilsson et al. 2019).

##### Distribution.

Basidiomata encountered in: Canada, Estonia, Finland, Norway, Sweden and the United Kingdom. Soil or root tip samples confirm presence also in: France, Poland, Spain and the United States.

##### Remarks.

The nomenclatural situation surrounding *P.umbrina* is complex. Fries described *Thelephoraumbrina*, explicitly excluding *Thelephoraumbrina* Pers., but synonymising it with Thelephoraumbrinavar.lignatilis Alb & Schwein. These names might represent different species or not, but in either case do not threaten *Thelephoraumbrina* Fr., due to the sanctioning.

A large number of names synonymous with *P.umbrina* have been illegitimately or superfluously published. Fries himself (1847), as well as Quélet (1888) and Burt (1916), seem to have overlooked Wallroth’s (1833) combination of *Hypochnusumbrinus* (Alb. & Schwein.) Wallr. from *Himantiaumbrina* Alb. & Schwein, (1805) and hence created illegitimate name combinations. The status of Donk’s (1933) combination of *Tomentellaumbrina* (Fr.) Donk versus Litschauer’s (1933) remains hard to evaluate due to the fact that, although 20 June is known to be the date of Litschauer’s publication, 7 Julyis when Donk defended the thesis wherein he published his combination; the publication date of Donk’s thesis was probably at an unknown point in time prior to that of his dissertation. Combinations based on *Thelephoraumbrina* where the combining authors cite Alb. & Schwein as authors of the basionym (e.g. Saccardo 1888) have to be considered miscitations, since there is no *Thelephoraumbrina* Alb. & Schwein.

Our interpretation of *Thelephoraumbrina* Fr. as the basionym follows Burt (1916), Litschauer (1933), Svrcek (1958) and Larsen (1967), although Rogers and Jackson (1943) considered it to be a synonym of *Coniophoraolivacea*. The name is not used here in the sense of what we today interpret as *C.olivacea*, but in the sense of Burt’s (1916) type selection.

The material in the Fries Herbarium cited by Burt (1916), as the type of *Thelephoraumbrina* Fr., constitutes a collection made by Fries at locus classicus, Femsjö, but in Fries’s own handwriting, it is identified as *Corticiumumbrinum* (Fr.) Fr., a name he combined *T.umbrina* to in 1874 (Fries 1874). Therefore, Burt’s typification cannot be considered a lectotype, but must be regarded as a neotype. We here designate an epitype from Femsjö, which matches the neotype morphologically.

Within the *P.tristis* group, basidiomata of *P.umbrina* can be recognised by their brown colour – blue or green colours are completely absent from immature parts and from the subhymenium of mature parts – their soft, rather elastic texture after drying and their microcharacters. *Pseudotomentellaumbrinascens* is very similar but has slightly different microcharacters (see key). *Hypochnusrhacodium* (only known from the type) is also similar but has hard, brittle basidiomata after drying.

##### Additional specimens studied.

CANADA. Newfoundland: Crooked Knife, mixed forest with *Betula*, *Alnus* and *Picea*, 99 m a.s.l., 10 September 2008, U. Kõljalg (TU 108084*);

ESTONIA. Põlva: Vastse-Kuuste, older *Pinus-Picea* mixed forest between Kiidjärve and Taevaskoja, near Maarja village, 22 September 2005, U. Kõljalg (TU 100329, 100339, 100340); Saare: Muhu, Kesselaid, Karjalasma forest, *Piceaabies* forest, 28 August 1998, Erast Parmasto (TAAM 174051); Põlva: Vastse-Kuuste, coniferous forest with *Pinus* and *Picea* along road between Kiidjärve and Taevaskoja, east of Ahja river, 18 August 2005, U. Kõljalg (TU 100194); Viljandi: Pääsmä laas, Sooma National Park, on a fallen *Betula* trunk over Halliste river, 7 September 2000, U. Kõljalg (TU 108538);

FINLAND. Kanta-Häme: Lammi, Kotinen Virgin Forest, 10 September 2001, U. Kõljalg (TU 108742, 108743, 108744); Etelä-Häme, Ruovesi, Siikaneva swamp islands, 14 September 1999, U. Kõljalg (TAAM 159809, 159810); Satakunta: Ilkaalinen, under *Picea* log and mosses, 29 August 2010, U. Kõljalg (TU 115017); Varsinais-Suomi: Parainen, Kuggö, 24 October 2009, P. Kunttu (TU115344*);

NORWAY. Oppland, Dovre, Hjerkinn, low alpine vegetation under *Salixphyllicifolia*, *Salixlapponica* and *Betulanana*, on soil with low pH, 14 September 2014, S. Svantesson 216, 221* (GB); Akershus: Asker, Skaugumåsen, boreonemoral, mixed forest on moderately alkaline, moderately nutrient-rich ground under, 23 September 2010, S. Svantesson (O F110268*); Troms: Kvænangen, Kvænangselva, boreal mixed forest on soil with low pH, 31 August 2013, B. Larsson and K.-H. Larsson (O F110269); Ibidem, boreal, deciduous forest on soil with intermediate pH, 31 August 2013, B. Larsson and K.-H. Larsson (O F110270, F110271); Oppland: Dombås, Hjerkinnholen, boreal, mixed forest on soil with low pH, 30 September 2013, K.-H. Larsson (O F110272, F110273, F110274, F110275, F110276, F110277); Sogn og Fjordane: Leikanger, Flætene-Vesterheim, boreonemoral, mixed forest on soil with low pH, 2 October 2012, K.-H. Larsson and S. Svantesson (O F110278, F110279, F110280); Sogn og Fjordane: Eid, Eitrefjellet, deciduous forest on soil with high pH, 25 September 2013, K.-H. Larsson (O F110281); Oppland, Dovre, Grimsdalen, Storberget, subalpine Betulapubescensssp.czerepanovii forest on soil with low pH, 26 August 2010, K.-H. Larsson and S. Svantesson (O F110282, F110283, F110284, F110285, F110286); Aust-Agder: Tvedestrand, Eidbo, boreonemoral, mixed forest on soil with intermediate pH, 10 September 2010, S. Svantesson and N. Svensson (O F110307); Aust-Agder: Åmli, Gangsei W, boreonemoral, mixed forest on soil with low pH, 09 September 2010, S. Svantesson and N. Svensson (O F110308); Telemark: Drangedal, Asgjerdstigfjellet, boreonemoral, deciduous forest on on soil with intermediate pH, 28 September 2010, S. Svantesson and N. Svensson (O F110309, F110310); Vest-Agder: Mandal, Uføra, nemoral, deciduous forest on soil with high pH, 26 September 2012, K.-H. Larsson and S. Svantesson (O F110287); Sogn og Fjordane: Leikanger, Kvinnafossen, boreonemoral, mixed forest on soil with high pH, 2 October 2012, K.-H. Larsson and S. Svantesson (O F110288); Nord-Tröndelag: Grong, Gartlandselva, boreal, coniferous forest on soil with low pH, 27 August 2012, K.-H. Larsson (O F110289, F110290, F110291, F110292, F110293, F110294); Nordland: Saltdal, Nystadneslia, boreal, mixed forest on soil with intermediate pH, 24 August 2012, K.-H. Larsson (O F110295, F110296*); Telemark: Tokke, Dalen, Huvestad, boreonemoral, mixed forest on soil with high pH, 28 September 2010, S. Svantesson and N. Svensson (O F110311); Akershus: Nannestad, Tomte farm, 3 September 2004, U. Kõljalg (TU 100005, 100007); Telemark: Sauherad, E of Nordagutu, W slope of Bjørndalsfjell along path to Svanastøl, 24 September 2003, K.-H. Larsson 12094 (TU); Buskerud: Nes, Alungruken, 25 September 1997, J. Stokland (TU 115209*), Rogaland: Forsand, Rössdalen, on *Salix* sp., 14 October 1998, K. Hjortstam 17918 (K.-H. Larsson private collection);

SWEDEN. Lycksele Lappmark: Storuman, Blaiken N, boreal, mixed, old-growth forest on fertile, moderately alkaline ground, 26 August 2015, S. Svantesson 137 (GB); Västerbotten: Umeå, Stora Tuvan, older, boreal, mixed forest on soil with low pH, 28 August 2015, S. Svantesson 174*, 175 (GB); Lule Lappmark: Gällivare, Ritsem, subalpine Betulapubescensssp.czerepanovii forest on soil with low pH, 11 August 2016, S. Svantesson 234, 239*, 240 (GB); Lule Lappmark: Jokkmokk, Slappejaure NO, middle alpine vegetation on soil with high pH, 14 August 2016, S. Svantesson 255, 256 (GB); Lule Lappmark: Jokkmokk, Unna Duvgge, low alpine vegetation on soil with intermediate pH, 15 August 2016, S. Svantesson 277 (GB); Lule Lappmark: Jokkmokk, Ajajaure N, low alpine vegetation on soil with high pH, 16 August 2016, S. Svantesson 279, 280* (GB); Halland: Kungsbacka, Släp, Särö Västerskog, old growth *Pinus* and *Quercus* forest, under a *Pinus* log, 1 October 1999 U. Kõljalg (TAAM 159818); Ångermanland; Sollefteå, Sörgraninge mångfaldspark, Språngsjöberget, 9 September 2014, K.-H. Larsson 16608 (GB); Västergötland: Alingsås, Simmenäshalvön, Gräskärr, on *Picea*, 5 October 2008, B. and K. Hjortstam 20311, 20332 (K.-H. Larsson private collection); Ibidem, on wood of *Quercus* on the ground, 13 September 2004, K. Hjortstam 18795 (K.-H. Larsson private collection); Ibidem, on *Picea* bark, 17 October 2001, K. Hjortstam 18531 (K.-H. Larsson private collection); Västergötland: Vårgårda, Nårunga, Sandviksås, on branch of *Quercusrobur*, 8 November 2000, Björn Nordén (TU 115240);) ; Öland: Böda, Fagerör, under log of *Pinussylvestris*, 15 October 2016, E. Larsson 387-16 (GB); Öland: Böda, Bryum Sandvik, under log of *Pinussylvestris*, 15 October 2016, E. Larsson 384B-16 (GB);

UNITED KINGDOM OF GREAT BRITAIN AND NORTHERN IRELAND. Scotland, Invernesshire: Glen Strathfarrar National Nature Reserve, ancient *Pinussylvestris* forest with a few oak trees, 14 September 2005, U. Kõljalg (TU 100304); Scotland, Morayshire: Culbin Forest, planted *Pinussylvestris* forest on sand dunes, 13 September 2005, U. Kõljalg (TU 100292).

#### Pseudotomentella umbrinascens

Taxon classificationFungiThelephoralesThelephoraceae

Svantesson sp. nov.

MB829031

Fig. 19

##### Type.

SWEDEN. Bohuslän: Tanum (municipality), Tanum (parish), Greby Kleva, boreonemoral, deciduous forest on soil with high pH, RT90: E1236840, N6518916, 6 September 2016, S. Svantesson 335 (holotype: GB!, GenBank Acc. No. ITS: MK290697)

##### UNITE SH.

SH030563.07FU

##### Etymology.

The name refers to the overall morphological similarity to *P.umbrina*.

##### Description.

**Basidiome** annual, resupinate, membranaceous, effused to approximately ten centimetres in diameter. Mature parts continuous, with a cottony texture when fresh and a rather firm, fibrous and compact, yet quite soft and elastic texture when dried. Hymenium smooth; greenish-brown when fresh, pale brown when dried. Immature parts discontinuous, byssoid with a cottony texture, both when fresh and when dried. Subhymenium and hymenium of the immature parts initially yellowish-white to pale brown, in the dried basidiome, when more mature pale brown. Subiculum well developed, loose, fibrous, pale yellowish-brown to pale orange brown; forms the outer edge of the basidiome, extending noticeably beyond the hymenium.

**Figure 19.**
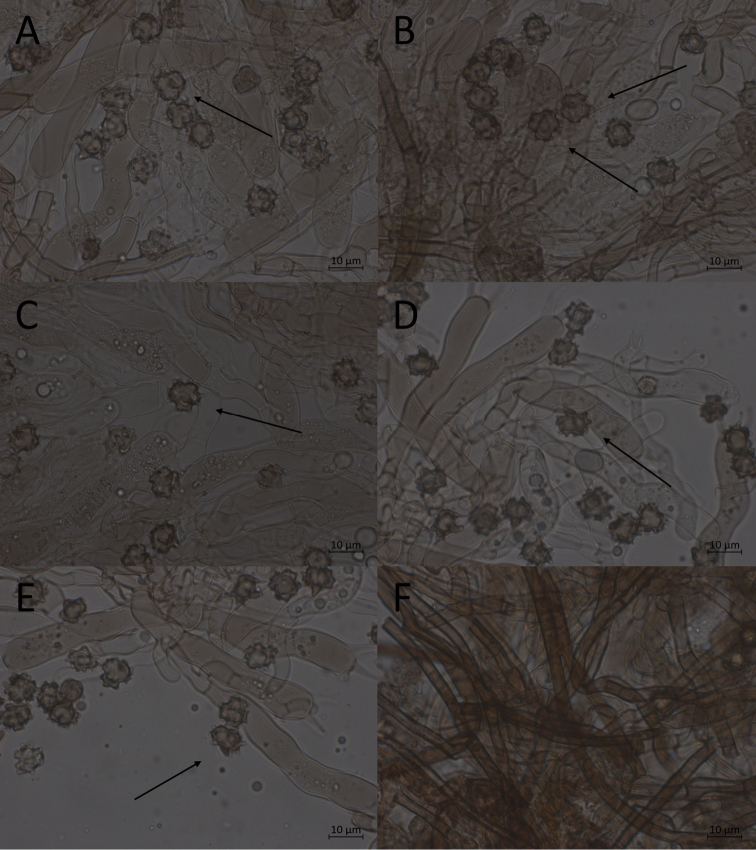
Micromorphological features of *P.umbrinascens* in KOH. Holotype: **A, B, C** basidiospores in frontal face **D, E** in lateral face **E** subicular hyphae.

**Hyphal cords** lacking, but loose bundles of subicular hyphae sometimes present.

**Hyphal system** monomitic, clamp connections and reaction in Melzer’s reagent absent from all hyphae.

**Subicular hyphae** noticeably long and straight, thick-walled; forming a loose tissue. Individual hyphae 3.1–) 3.2–4.3 (–4.8) μm wide, with a mean width of 3.7 μm; pale orange brown to brown in KOH, orange brown in water.

**Subhymenial hyphae** often somewhat sinuous, thin to thick-walled; forming a rather dense tissue. Individual hyphae 3.6–5.7 (–7.1) μm wide, with a mean width of 4.5 μm; pale grey brown to grey brown or brown in KOH; orange brown to pale green in water (but not with the blue green reaction present in other species), with strongly granular contents.

**Encrustation** not seen.

**Basidia** with four slightly curved sterigmata, occasionally two-sterigmate; clavate to narrowly clavate, sometimes clavopedunculate, thin-walled, with one-three slight constrictions. Dimensions: (57–) 58–71 (–75) × (8.8–) 9.5–11.5 (–12.5) μm; mean dimensions: 64 × 10.6 μm. Sterigmata 8.1–9.5 (–10.1) μm long, with a mean length of 8.6 μm. Colours and reactions the same as for the subhymenial hyphae, but in addition sometimes with granular contents in KOH.

**Cystidial organs** lacking.

**Basidiospores** in frontal face generally with a triangular or subcircular basic shape and an angular to cross-shaped or sometimes nodulose outline, covered in bi- or trifurcate, sometimes singularly attached, echinuli. Nearly all spores with three-four distinct, often rounded lobes; subcircular, five-lobed spores infrequently occurring, as well as abnormally large spores originating from two-sterigmate basidia. Frontal dimensions: (8.5–) 8.7–9.4 (–9.6) × (8.4–) 8.7–9.2 (–9.3) μm; mean dimensions: 8.9 × 8.9 μm; Q-value: 1.0 (–1.1); mean Q-value: 1.0. Echinuli (0.9–) 1.0–1.9 (–2.0) μm long, with a mean length of 1.6 μm. Lateral face ellipsoid to narrowly ovoid or sometimes semicircular in shape, usually with evenly rounded edges, sometimes with one-three lobes. Lateral dimensions: 8.5–9.2 (–9.4) × (5.7–) 6.0–6.5 μm; mean dimensions: 8.9 × 6.2 μm; Q-value: 1.3–1.5 (–1.6); mean Q-value: 1.4. Colour in KOH pale brown to pale greenish-brown colour; in water pale brownish-orange to pale greenish-orange; inamyloid.

**Chlamydospores** lacking.

##### Habitat.

The only specimen recorded to date of *P.umbrinascens* is the type collection, which was obtained in an old, coastal, deciduous forest on soil with high pH. UNITE sequence metadata shows that the species forms ectomycorrhiza with at least *Corylusavellana* (Kõljalg et al. 2005, Nilsson et al. 2019).

##### Distribution.

Basidiomata encountered in: Sweden. Soil or root tip samples confirm presence also in: Italy.

##### Remarks.

Within the *P.tristis* group, basidiomata of *P.umbrinascens* can be recognised by their brown colour, their soft, rather elastic texture after drying and their microcharacters. Blue or green colours are completely absent from immature parts and from the subhymenium of mature parts. *Pseudotomentellaumbrina* is very similar but has slightly different microcharacters (see key). *Hypochnusrhacodium* (only known from the type) is also similar but has basidiomata that are hard and brittle after drying.

### Dubious taxa

#### Pseudotomentella longisterigmata

Taxon classificationFungiThelephoralesThelephoraceae

M.J.Larsen, Canad. J. Bot. 45: 1298 (1967)

Fig. 20

##### Type.

UNITED STATES. Washington: Olympic Peninsula, Sol Duc River, on coniferous wood, 25 August 1957, J. L. Lowe 8061 (holotype: BPI US0291345!; isotype: SYRF).

##### Description.

**Basidiome** annual, resupinate, membranaceous, effused to approximately ten centimetres in diameter. Mature parts continuous, with a cottony to rather firm, fibrous and compact, yet quite soft and elastic texture. Hymenium smooth; bluish-grey to brownish-grey. Immature parts discontinuous, byssoid with a cottony texture. Subhymenium and hymenium of immature parts bluish-grey. Subiculum well-developed, loose, fibrous, orange brown; forms the outer edge of the basidiome, extending noticeably beyond the hymenium. All characters recorded in dried state.

**Figure 20.**
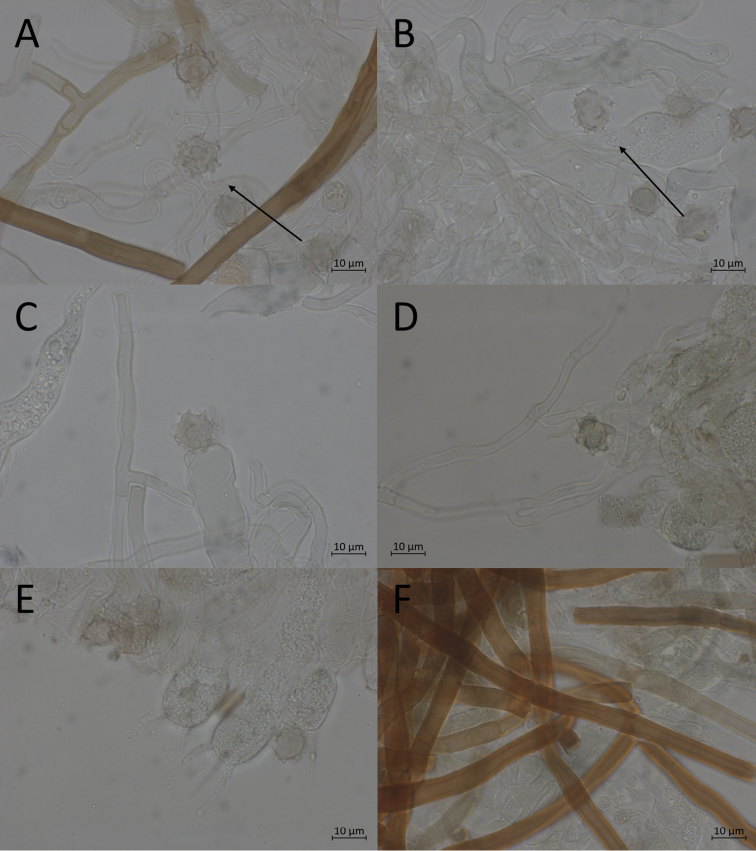
Micromorphological features of *P.longisterigmata* in KOH. Holotype: **A, B, C** basidiospores in frontal face **D** in lateral face **E** basidia **F** subicular hyphae.

**Hyphal cords** lacking, but loose bundles of subicular hyphae sometimes present.

**Hyphal system** monomitic, clamp connections absent from all hyphae.

**Subicular hyphae** noticeably long and straight, thick-walled; forming a loose tissue. Individual hyphae 4.9–7.2 μm wide, with a mean width of 6.2 μm; orange brown to brown in KOH, orange to orange brown in water.

**Subhymenial hyphae** often somewhat sinuous, thin to thick-walled; forming a rather dense tissue. Individual hyphae (3.0–) 3.2–4.9 (–6.1) μm wide, with a mean width of 3.9 μm; pale brownish-green in KOH, blue green in the presence of air; brownish-green in water, with strongly granular contents.

**Encrustation** granular, probably amyloid (hard to observe due to the colour); dark brownish-green in KOH, dark blue green in the presence of air; blackish in water; scattered in occurrence on the upper parts of subhymenial hyphae and on the lower parts of basidia.

**Basidia** with four very long, slightly curved to hypha-like sterigmata, occasionally two-sterigmate; clavate or sometimes clavopedunculate, thin-walled, with one-three slight constrictions. Dimensions: (73–) 77–110 (–121) × (12.3–) 13.0–15.1 (–16.3) μm; mean dimensions: 91 × 13.9 μm. Sterigmata (11.2–) 11.7–17.9 (–19.3) μm long, with a mean length of 14.7 μm. Colours and reactions the same as for the subhymenial hyphae, but in addition often with granular contents in KOH.

**Cystidial organs** lacking.

**Basidiospores** in frontal face generally with a subcircular basic shape and an angular to nodulose or sometimes cross-shaped outline, covered in bi- or trifurcate, sometimes singularly attached, echinuli. A majority of the spores with three-five indistinct lobes; unlobed subellipsoid or broadly ovoid spores present to a lesser extent, as well as subcircular, six or seven-lobed spores; abnormally large spores originating from two-sterigmate basidia infrequently occurring. Frontal dimensions: (9.7–) 10.0–11.7 × (9.4–) 9.8–11.7 μm; mean dimensions: 11.0 × 10.7 μm; Q-value: (0.9–) 1.0 (–1.1); mean Q-value: 1.0. Echinuli 1.2–1.8 (–2.1) μm long, with a mean length of 1.5 μm. Lateral face ellipsoid to ovoid, usually with evenly rounded edges, sometimes with one-three lobes. Lateral dimensions: 10.3–11.5 (–11.7) × (6.7–) 7.5–9.1 μm; mean dimensions: 10.9 × 8.5 μm; Q-value: 1.2–1.6; mean Q-value: 1.3. Colour in KOH pale brown to pale greenish-brown, in the presence of air often with a green to blue green reaction; in water brown; occasionally amyloid.

**Chlamydospores** lacking.

##### Habitat.

The only specimen of *P.longisterigmata* recorded to date is the type collection, which was collected on coniferous wood in a coastal forest in the state of Washington, United States.

##### Distribution.

Basidiomata encountered in: the United States.

##### Remarks.

The type collection is large and in seemingly good condition but repeated attempts at obtaining a useful DNA sequence from it proved unfruitful. The specimen exhibits a peculiar morphology, where the basidia carry sterigmata that are unusually long for the *P.tristis* group. They are often cylindrical rather than tapering and hence resemble generative hyphae – a growth form that basidia are sometimes seen reverting into in corticioid basidiomata formed under unfavourable conditions. It is therefore doubtful whether *P.longisterigmata* is a true species, but since this presently cannot be ascertained and, in order to stimulate its recollection, the name is here retained as a separate species.

There are relatively few spores in the hymenium of the holotype and many of them are immature. A more mature fruiting body of the species would hence probably have a browner colour.

Within the *P.tristis* group, the basidiome of *P.longisterigmata* can be recognised by its lack of hyphal cords and skeletal hyphae, its soft, yet rather firm and compact and ± elastic texture after drying, bluish to greenish colour of immature parts, wide subicular hyphae, large spores and long sterigmata. *Pseudotomentellaalobata*, *P.pluriloba* and *P.abundiloba* are similar but all have smaller spores and shorter sterigmata.

#### Hypochnus rhacodium

Taxon classificationFungiThelephoralesThelephoraceae

Berk. & M.A.Curtis ex Burt, Ann. Missouri Bot. Gard. 13(3): 322 (1926).

Fig. 21

##### Type.

UNITED STATES OF AMERICA. Pennsylvania: on underside of decaying logs of apparently a frondose species, E. Michener 1435 (syntypes: Mo. Bot. Gard. Herb. 5095 [BPI US0291002]!; FH Curtis Herb. 4061; K Curtis Herb. 4061, designated by E.A. Burt in Ann. Missouri Bot. Gard. 13: 322 (1926)).

##### Description.

**Basidiome** annual, resupinate, membranaceous, effused. Mature parts continuous, with a hard and rather brittle texture. Hymenium smooth; pale brown to brown with a pink hue. Subiculum loose, fibrous and dark brown with an orange hue. All characters recorded in dried state.

**Figure 21.**
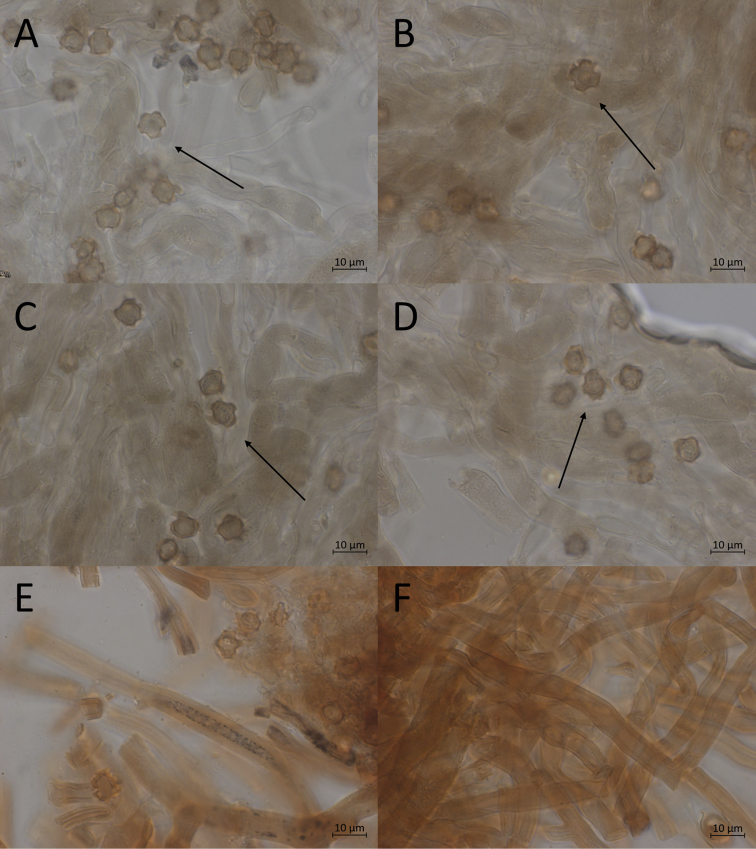
Micromorphological features of *H.rhacodium* in KOH. Syntype: **A, B** basidiospores in frontal face **C, D** in lateral face **E** subicular hyphae with granulation and **F** without.

**Hyphal cords** lacking, but loose bundles of subicular hyphae sometimes present.

**Hyphal system** monomitic, clamp connections and reaction in Melzer’s reagent absent from all hyphae.

**Subicular hyphae** noticeably long and straight, thick-walled; forming a loose tissue. Individual hyphae (5.6–) 5.7–7.3 (–8.0) μm wide, with a mean width of 6.5 μm; pale to dark orange brown in both KOH and water, sometimes with granular contents which turn blue green in the presence of air.

**Subhymenial hyphae** often somewhat sinuous, thin to thick-walled; forming a rather dense tissue. Individual hyphae (3.7–) 3.8–5.3 (–6.1) μm wide, with a mean width of 4.7 μm; brown to pale orange brown or pale green in KOH (but not with the blue green reaction present in other species); orange brown to brown in water, with strongly granular contents.

**Encrustation** granular, probably amyloid (hard to observe due to the colour); brownish-black in KOH, dark blue green in the presence of air; brownish-black in water; scattered in occurrence on the subicular hyphae.

**Basidia** with four slightly curved sterigmata, occasionally two-sterigmate; narrowly clavate or sometimes narrowly clavopedunculate, thin-walled, with one-three slight constrictions. Dimensions: 73–105 (–109) × (8.8–) 8.9–10.1 (–11.2) μm; mean dimensions: 94 × 9.6 μm. Sterigmata (8.5–) 9.5–12.1 (–12.5) μm long, with a mean length of 10.9 μm. Colours and reactions the same as for the subhymenial hyphae.

**Cystidial organs** lacking.

**Basidiospores** in frontal face generally with a subcircular basic shape and an angular to nodulose or sometimes cross-shaped outline, covered in bi- or trifurcate, sometimes singularly attached, echinuli. Nearly all spores with three-five distinct, rounded to square lobes; abnormally large spores originating from two-sterigmate basidia infrequently occurring. Frontal dimensions: (7.8–) 8.0–9.1 (–9.3) × (7.7–) 7.8–8.9 (–9.0) μm; mean dimensions: 8.3 × 8.3 μm; Q-value: 0.9–1.1; mean Q-value: 1.0. Echinuli (0.9–) 1.0–1.6 (–1.7) μm long, with a mean length of 1.3 μm. Lateral face ellipsoid to ovoid or sometimes subcylindrical, usually with angular edges, sometimes with one-three lobes. Lateral dimensions: (7.9–) 8.2–8.9 × (5.4–) 5.9–6.8 (–7.0) μm; mean dimensions: 8.5 × 6.3 μm; Q-value: 1.2–1.4 (–1.6); mean Q-value: 1.3. Colour in KOH brown to orange brown, in the presence of air with a blue green reaction; in water greenish-orange to orange brown; inamyloid.

**Chlamydospores** lacking.

##### Habitat.

The only specimen of *H.rhacodium* recorded to date is the type collection, which was collected in Pennsylvania, United States. No further information on habitat or any further locality description is available.

##### Distribution.

Basidiome encountered in: the United States.

##### Remarks.

The hymenium of *H.rhacodium* is very thick and dense in comparison to all other *Pseudotomentella* species. It consists of tightly packed basidia, which are overlapping in length and have a total thickness equalling four-six basidial lengths. All other morphological characters fit well within the *P.tristis* group, thus suggesting an abnormal basidiome.

Within the *P.tristis* group, the basidiome of *H.rhacodium* can be recognised by its lack of hyphal cords and skeletal hyphae and its hard and brittle texture after drying. This feature makes it unique within the group and the risk for confusion with any other described species should hence be small.

### Excluded taxa

#### Septobasidium arachnoideum

Taxon classificationFungiThelephoralesThelephoraceae

(Berk. & Broome) Bres., Ann. Mycol. 14 (3-4): 241 (1916)

##### Homotypic names.

*Thelephoraarachnoidea* Berk. & Broome, J. Linn. Soc., Bot. 14: 64 (1873) [1875]. *Hypochnusarachnoideus* (Berk. & Broome) Bres., Ann. Mycol. 1(2): 108 (1903). *Tomentellaarachnoidea* (Berk. & Broome) Höhn. & Litsch., Wiesner Festschrift: 77 (1908).

##### Type.

CEYLON [Nowadays Sri Lanka]. Habgalla, Feb. 1868, [M. J. Berkeley and C. E. Broome] No. 539 (K).

##### Remarks.

Bresadola (1916) combined *Thelephoraarachnoidea* Berk. & Broome to *Septobasidium*, and the species was accepted by Couch (1938) in his detailed review of the genus. We thus have no reason to believe that this species belongs in *Pseudotomentella*.

#### Tomentella biennis

Taxon classificationFungiThelephoralesThelephoraceae

(Fr.) A.M.Rogers Mycologia 40(5): 634 (1948)

##### Homotypic names.

*Auriculariaphylacteris* Bull., Herb. France 10: plate 436, fig. 2 (1790). *Thelephoraphylacteris* (Bull.) J.F.Gmel., Syst. nat. 2 (2): 1441 (1792) [combination also made by de Candolle (1815)]. *Thelephorabiennis*, Fr., Syst. mycol. 1: 449 (1821), sanctioned name. *Phylacteriabiennis* (Fr.) Bigeard, Fl. champ. sup. France 2: 452 (1913).

##### Type.

Bulliard JBF (1790) Herbier de la France, ou Collection complette des plantes indigenes de ce royaume; Avec leurs Détails Anatomiques, leurs propriétés, et leurs usages en Medecine. Tome 10, plate 436, figure 2, LECTOTYPE of *Auriculariaphylacteris*, here designated (Mycobank Typification No. MBT384912), LECTOTYPE of *Thelephorabiennis*, here designated (MBT384913).

##### Remarks.

Bulliard (1790) described *Auriculariaphylacteris* and Gmelin (1792) combined it to *Thelephora*. Seemingly, both Fries and de Candolle (1815) overlooked Gmelin’s combination. Fries (1821) created the name *Thelephorabiennis*, citing under it *A.phylacteris* and *T.phylacteris* DC, but seems to attribute it to de Candolle in the index of the Systema Mycologicum 3 (Fries 1832). This is probably an error and since Fries is the original author of the name, we agree with Petersen (1975) that the authorship is *Thelephorabiennis* Fr.

Fries (1821) indicated “v.ic.”, which would be a reference to the plate in Bulliard (1790). There are no specimens under any of the aforementioned names known to have been examined by Bulliard, de Candolle or Fries. Consequently Bulliard’s plate of *A.phylacteris* (1790), mentioned by Fries (1821), is here designated as the lectotype of *A.phylacteris* and *T.biennis*.

The protologue of *T.biennis* describes and the plate of *A.phylacteris* depicts a species which is plicated at the lower part of the basidiome, yellowish-white when young, brown when older and which eventually turns black. It is further described as biennial and growing up from the ground and on to stones and branches, if they are present in its vicinity. It is hence doubtful whether the species in question belongs to the Thelephorales at all and, even though it has been synonymised with *P.umbrina* by Burt (1916), Litschauer (1933) and Svrček (1958), it does not match any *Pseudotomentella* described to date.

## Discussion

In a world where unseen and undescribed new phyla hide in a grain of soil (Nilsson et al. 2016) and where visible, morphologically delimited taxa increasingly turn out to constitute cryptic species (e.g. Kroken and Taylor 2001, Shivas and Cai 2012), it is reassuring to note that some visible, molecularly delimited species can still be separated morphologically. Similarly to many other fungal species, we, nevertheless, found *P.tristis* s.l. to constitute a complex of closely related and morphologically very similar species (e.g. Lücking et al. 2014, Jeppson et al. 2017 and Larsson et al. 2018). Thus Hjortstam’s (1969) interpretation of *P.tristis* and *P.umbrina* as two different species was indeed correct, but so was Larsen’s (1971a) argument that the variation he observed could not be separated into two species; under the name *P.tristis* are hiding no less than 13 species exhibiting morphological characteristics so close in range that they would indeed seem like a continuum to all mycologists without the aid of molecular analysis methods. In the light of our resurrection of *P.umbrina* as a separate taxon and the reviewed delimitation of *P.tristis* and *P.atrofusca*, this study not only proves the importance of combining molecular analysis methods with careful morphological studies, but also shows the power of these in conjunction with type studies. In the case of *H.rhacodium* and *P.longisterigmata*, the problems that can arise from species descriptions based solely on morphology are also clearly demonstrated.

From the perspective of functionality and usability of the international DNA sequence databases, it is satisfying to acknowledge that, while metadata from ecological studies have been very useful for understanding the molecularly delimited taxa presented here, future ecological studies querying such databases now have more reliable names to use. One species in the *P.tristis* group – *P.umbrina* – was indeed found to be widespread, have a wide ecological amplitude and, at least in northern Europe, to be commonly occurring. This is not to say that all the other species in the group are less widely distributed or have narrower ecological niches; some species, for example, *P.pinophila*, *P.tristis* itself and *P.sciastra*, have been collected in widely separated countries and habitats, but in comparison to *P.umbrina* they have rarely been encountered so far. More material is needed to establish the frequency of occurrence and ecological niches of all species in the species complex – information that might prove a helpful complement to morphology in the process of species identification, given the high degree of similarity between some species. With the current knowledge, it is quite paradoxical that the combination *P.tristis* was made by an American mycologist (Larsen 1971a), while the species in question now has no confirmed findings in North America. In contrast, *P.atrofusca*, a species believed to be widely distributed in Europe (GBIF 13–08–2018) and documented from the Russian far east (Kõljalg 1996), is now only known with certainty from the North American type collection and three sequences from China. South East Asia and Russia generally constitute large white spaces on the mycological map, even though findings so far indicate that species in the *P.tristis* group do occur in these areas. Even after taking the ecological knowledge gap into account, it is interesting to note that, unlike species in ectomycorrhizal genera such as *Leccinum* Gray and *Hygrophorus* Fr. that show strong host preferences and have more limited distribution ranges (den Bakker et al. 2004, Moreau et al. 2018), most species in the *P.tristis* group are able to form ectomycorrhiza with a broad range of hosts and are widely distributed. The fact that all species, except for *P.umbrina*, seem to be restricted to areas where soil pH is intermediate or high is possibly a factor that could help explain their difference in occurrence frequency.

The present study clarifies the application of the name *P.tristis*. In doing so, however, it renders hundreds of previous molecular ecology studies obscure with respect to this particular name. The name of *P.tristis* has served as something of a wastebasket for any and all *Pseudotomentella* species, owing both to the obscure nature of the underlying taxonomy and to the noisy state of taxonomic annotations in the public sequence databases. Thus, while the present study clarifies the use of the name *P.tristis*, it also raises doubts about previous molecular ecology results in the context of this name. To the extent that previous studies have relied on UNITE Species Hypotheses identifiers rather than Latin names when reporting molecular ecology results, this problem will be solved automatically. However, any study that tied species occurrences only to Scientific names may, from now on, convey incorrect information in the context of *Pseudotomentella*.

To the extent that it can be assessed given the moderate phylogenetic resolution, it is intriguing that the morphological characters that differ between species (e.g. spore size and shape, subicular hyphal width) do not seem to have a strong phylogenetic link. Whether these absences of patterns have the same cause, for example, an old rapid radiation, with extensive gene flow or are just artefacts of time and chance – causing both intragenic mutational conflict and genetic drift towards evolutionarily neutral shifts in morphology – is unclear, but could possibly be resolved by analysis of additional genetic regions. This may also shed some light on the presence of paralogous relationships between some of the taxa in the group and would possibly resolve some species into additional new species. The considerable genetic and morphological variation exhibited by *Pseudotomentellasciastra*, for example, may well indicate a species complex. Both ASTRAL and STACEY should be robust with the relatively small datasets used in the present study, in the sense that the employed datasets should not include less species than the analyses support. Additional specimen sampling may, however, aid in the distinction between populations and any possible, additional species and would thus, besides widened gene sampling, also be preferable in an extended study of the group.

Concerning morphology, the presence of amyloid material in and on basidia and subhymenial hyphae of Thelephorales species does not seem to have been reported. This is surprising, given its possible usefulness as a discriminatory character between species. Whether the cause of this is rarity or obscurity remains to be revealed by further studies in the field. Similarly worthy of notice is the blue green reaction observed in the same micromorphological structures of some species. Such a reaction has been mentioned by others studying *Tomentella* and *Pseudotomentella* (Larsen 1971a, Kõljalg 1996), but we would like to draw attention to the observation that the reaction in question here only seems to occur in the presence of air and also to its probable usefulness as a species-separating character. Finally, this study demonstrates clearly the necessity of applying a well-developed and consistent methodology when assessing the morphological characters of closely related species. It cannot be emphasised enough how important it is for those who endeavour to correctly identify Thelephorales species to carefully measure spores using the methodology originally described by Kõljalg (1996) and further explained in the Methods sections of this paper.

## Supplementary Material

XML Treatment for Pseudotomentella abundiloba

XML Treatment for Pseudotomentella alnophila

XML Treatment for Pseudotomentella alobata

XML Treatment for Pseudotomentella atrofusca

XML Treatment for Pseudotomentella media

XML Treatment for Pseudotomentella pinophila

XML Treatment for Pseudotomentella pluriloba

XML Treatment for Pseudotomentella rotundispora

XML Treatment for Pseudotomentella sciastra

XML Treatment for Pseudotomentella tristis

XML Treatment for Hypochnus sitnensis

XML Treatment for Pseudotomentella tristoides

XML Treatment for Pseudotomentella umbrina

XML Treatment for Pseudotomentella umbrinascens

XML Treatment for Pseudotomentella longisterigmata

XML Treatment for Hypochnus rhacodium

XML Treatment for Septobasidium arachnoideum

XML Treatment for Tomentella biennis

## Supplementary materials

Supplementary material 1 Solving the taxonomic identity of *Pseudotomentellatristis* s.l. (Thelephorales, Basidiomycota) Data type: (phylogenetic trees/neighbour nets) Explanation note: Phylogenetic trees (BEAST and PHYML) of the Tef1α and mtSSU regions and neighbour nets of the nrDNA, Tef1α and mtSSU alignments. This dataset is made available under the Open Database License (http://opendatacommons.org/licenses/odbl/1.0/). The Open Database License (ODbL) is a license agreement intended to allow users to freely share, modify, and use this Dataset while maintaining this same freedom for others, provided that the original source and author(s) are credited.Sten Svantesson, Karl-Henrik Larsson, Urmas Kõljalg, Tom W. May, Patrik Cangren, R. Henrik Nilsson, Ellen Larsson
