# Supplementary material for: Solving the taxonomic identity of Pseudotomentellatristis s.l. (Thelephorales, Basidiomycota) – a multi-gene phylogeny and taxonomic review, integrating ecological and geographical data
Source: MycoKeys. 2019 Apr 4;50:1–77. doi: 10.3897/mycokeys.50.32432 (PMC6477855; doi:10.3897/mycokeys.50.32432)
Supplement: Supplementary material 1 [file mycokeys-50-001-s001.docx]

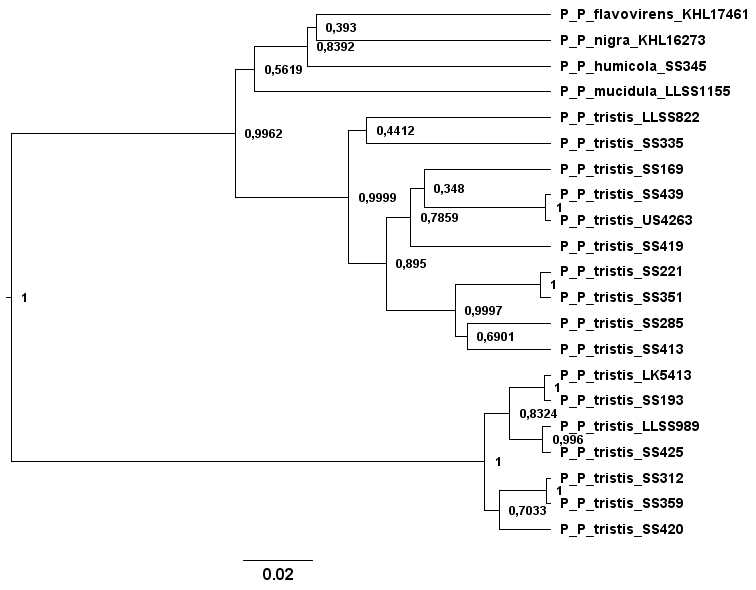


**Figure S1. Bayesian Tef1α-phylogeny of the *P. tristis* group.** Values between 0 and 1 denote posterior probability. The branch lengths are scaled in substitutions/site.


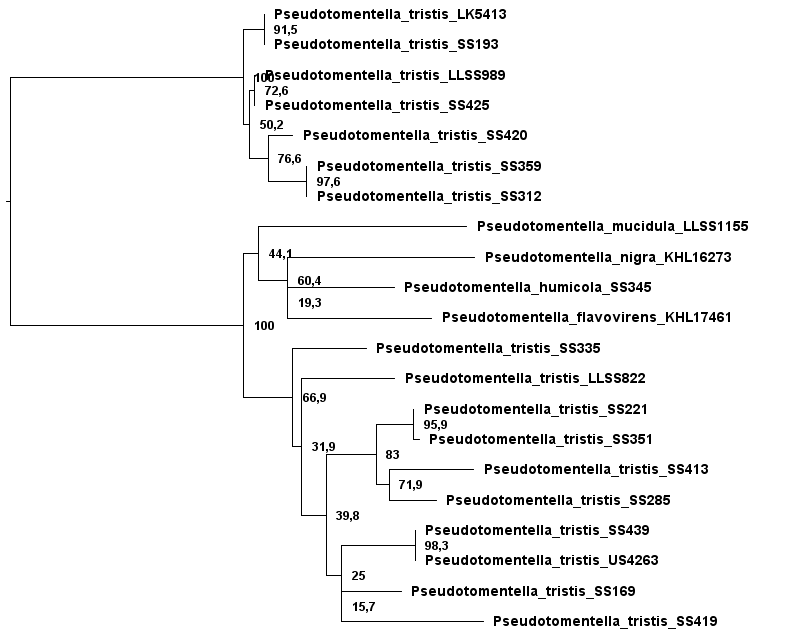


**Figure S2. ML Tef1α-phylogeny of the *P. tristis* group.** Values between 0 and 100 denote bootstrap support.


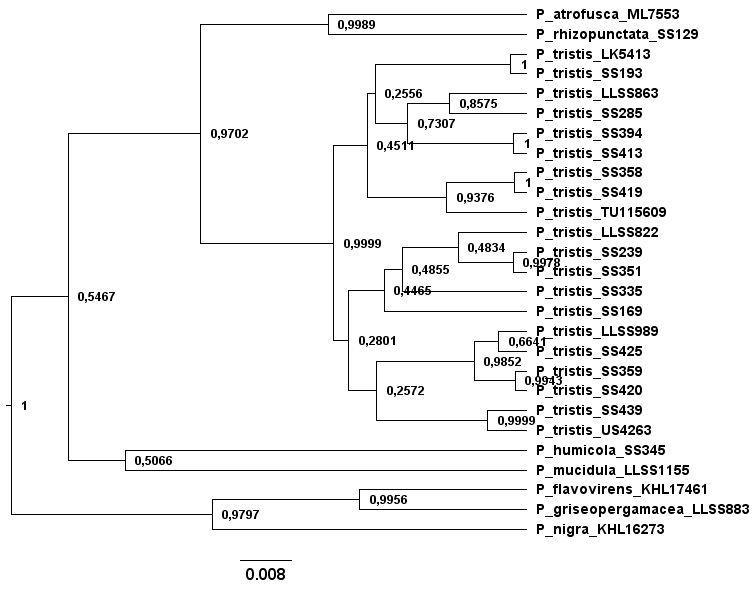


**Figure S3. Bayesian mtSSU-phylogeny of the *P. tristis* group.** Values between 0 and 1 denote posterior probability. The branch lengths are scaled in substitutions/site.


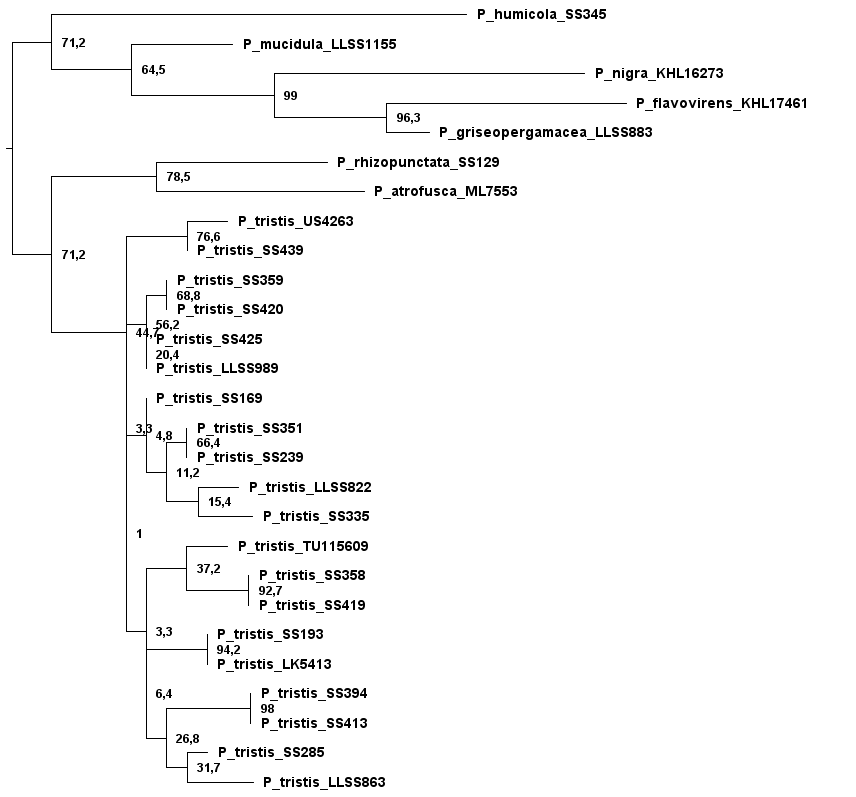


**Figure S4. ML mtSSU-phylogeny of the *P. tristis* group.** Values between 0 and 100 denote bootstrap support.


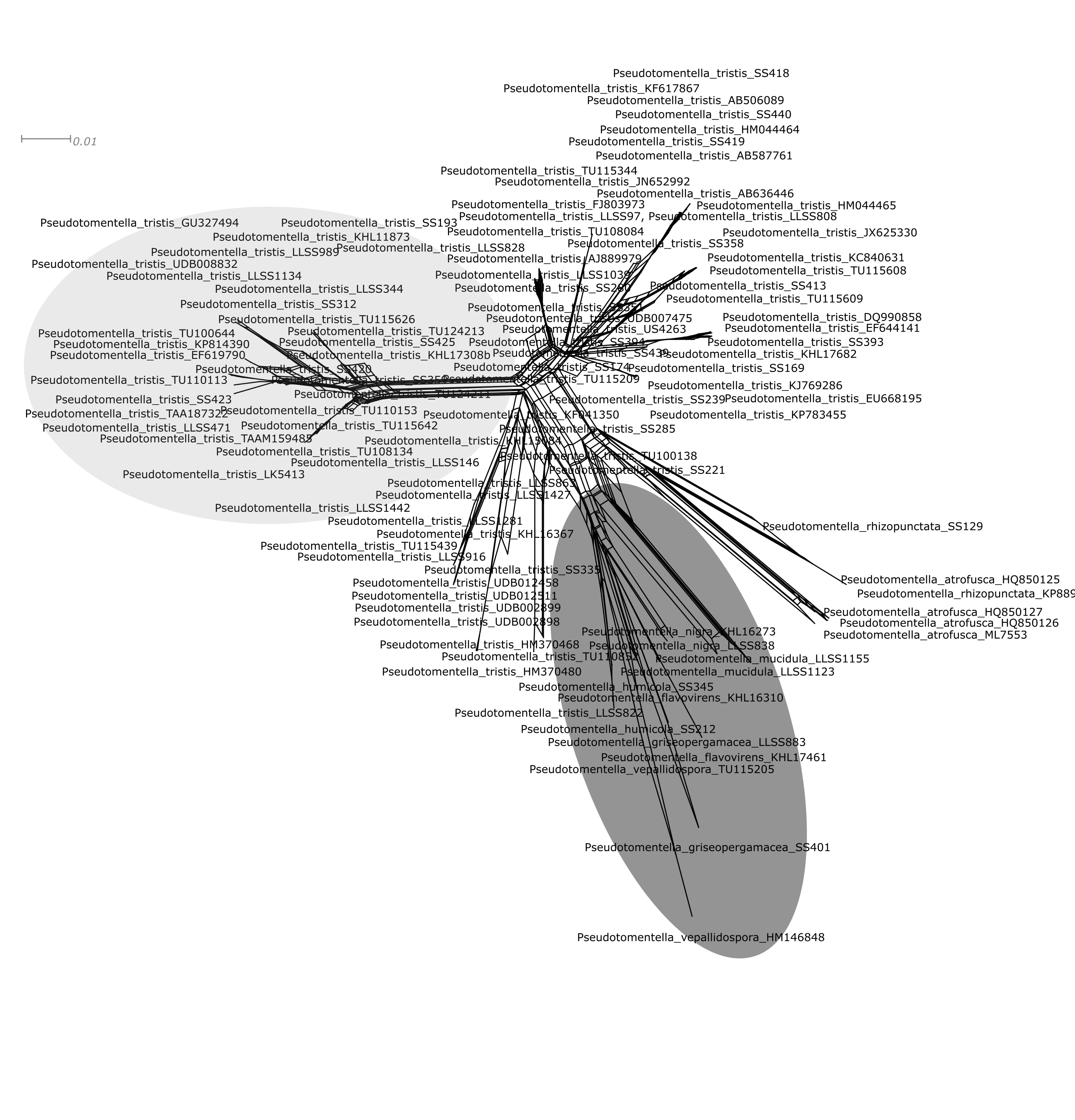


**Figure S5. Neighbour net of the rDNA alignment.** The branch carrying the species *P. sciastra*, *P. tristoides*, *P. alobata* and *P. tristis* is shown in pale grey and the outgroup is highlighted in darker grey. The network is scaled in uncorrected pairwise distances.


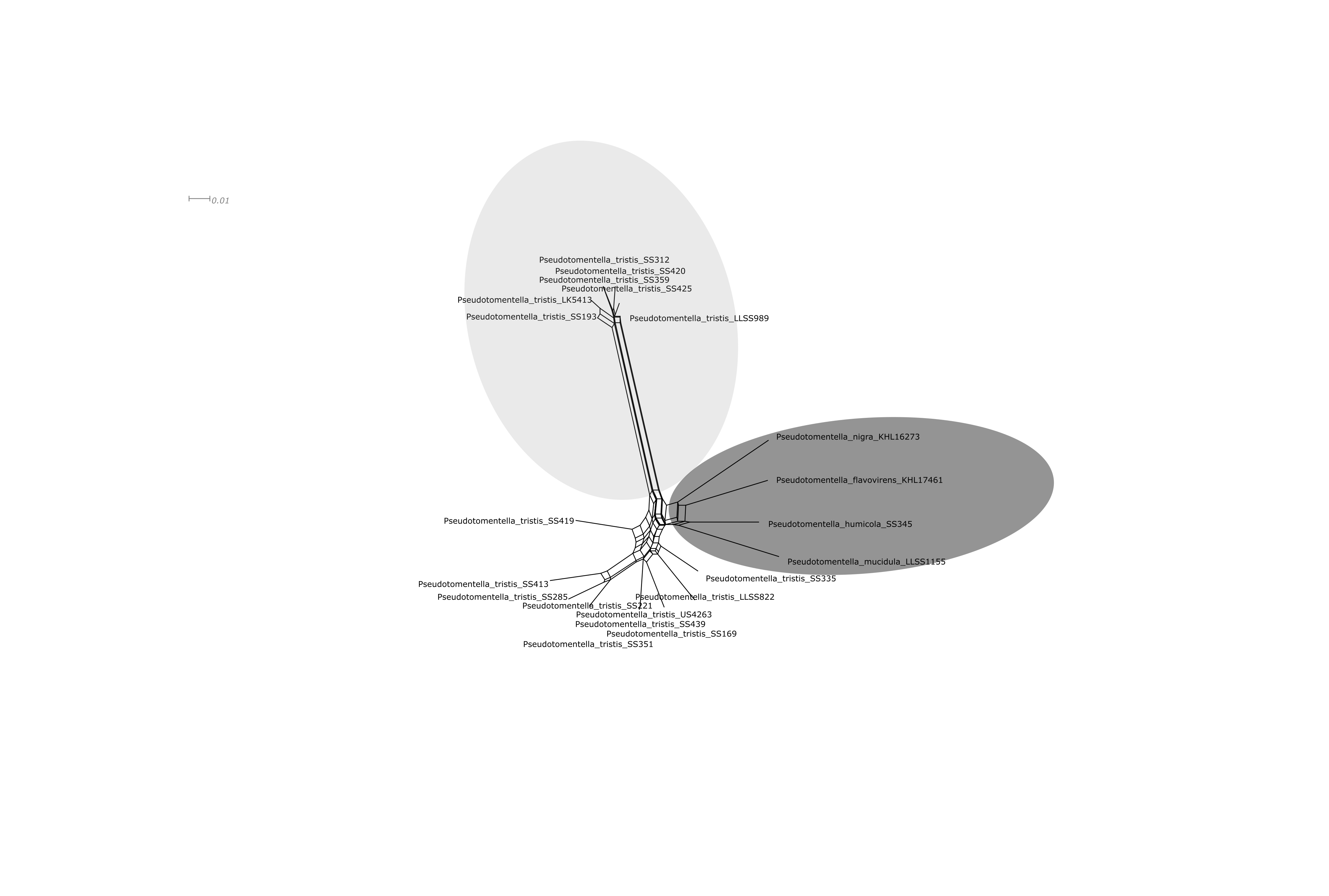


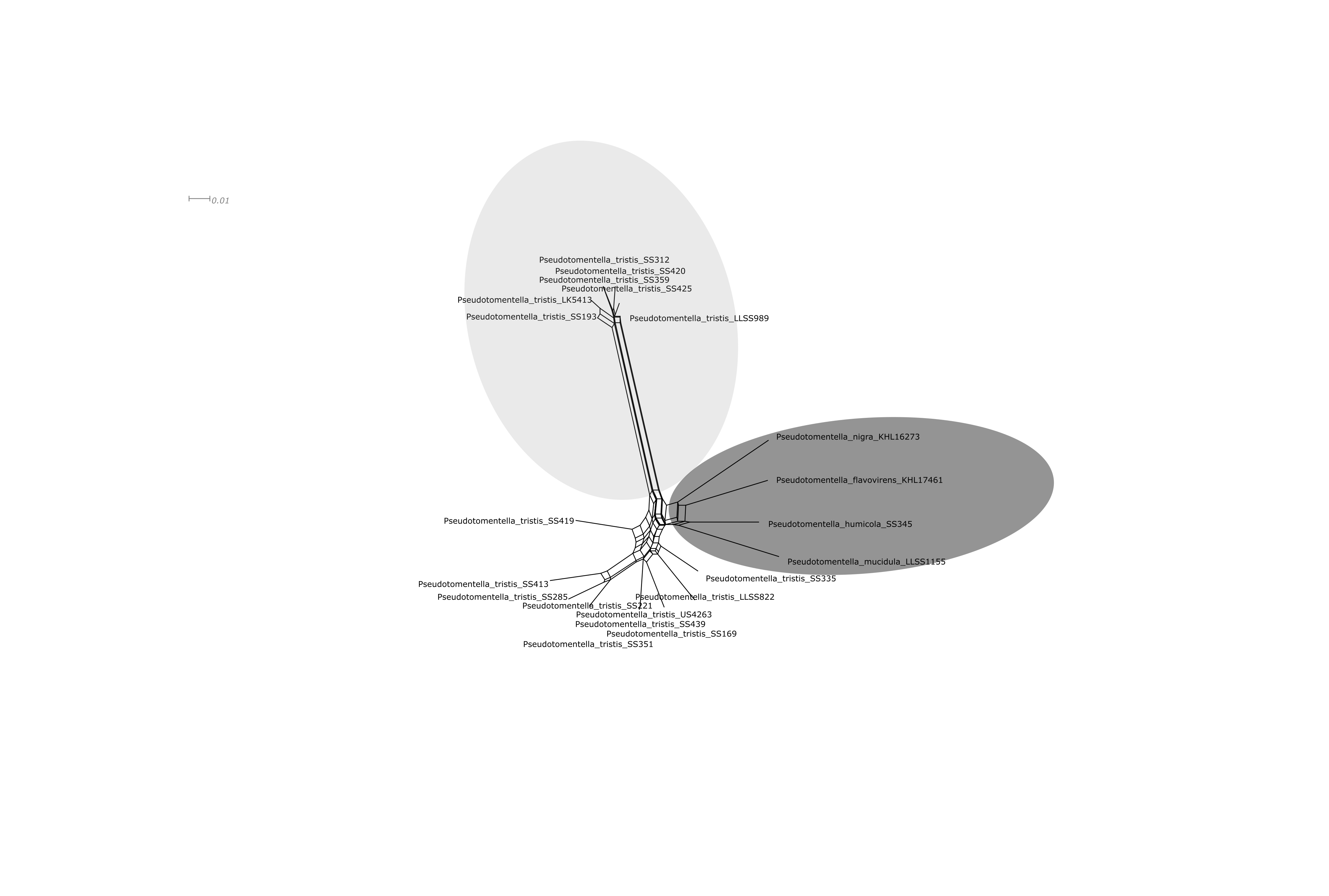

**Figure S6. Neighbour net of the Tef1α alignment.** The branch carrying the species *P. sciastra*, *P. tristoides*, *P. alobata* and *P. tristis* is shown in pale grey and the outgroup is highlighted in darker grey. The network is scaled in uncorrected pairwise distances.


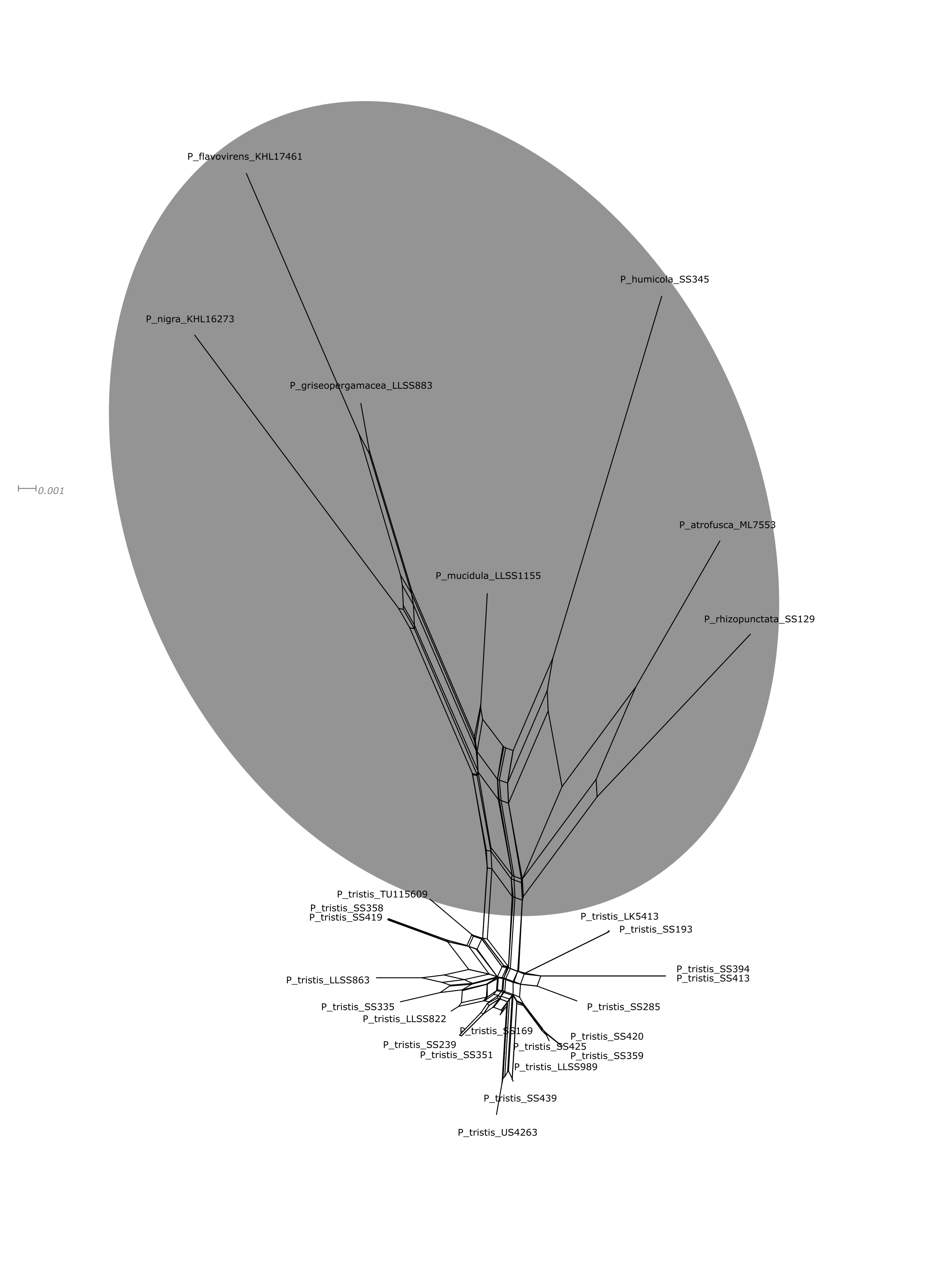


**Figure S7. Neighbour net of the mtSSU alignment**. The outgroup is highlighted in grey. The network is scaled in uncorrected pairwise distances.
